# Systematic chemoenzymatic synthesis of *O*-sulfated sialyl Lewis *x* antigens†
†Electronic supplementary information (ESI) available: Materials, experimental details of the synthesis, analytical data of **4–10**, **1a–3a**, and **1b–3b**, and NMR spectra of synthesized compounds. See DOI: 10.1039/c5sc04104j


**DOI:** 10.1039/c5sc04104j

**Published:** 2015-12-17

**Authors:** Abhishek Santra, Hai Yu, Nova Tasnima, Musleh M. Muthana, Yanhong Li, Jie Zeng, Nicholas J. Kenyon, Angelique Y. Louie, Xi Chen

**Affiliations:** a Department of Chemistry , University of California , Davis One Shields Avenue , Davis , CA 95616 , USA . Email: xiichen@ucdavis.edu ; Fax: +1-530-752-8995 ; Tel: +1-530-754-6037; b School of Food Science , Henan Institute of Science and Technology , Xinxiang , 453003 , China; c Division of Pulmonary, Critical Care and Sleep Medicine , Department of Internal Medicine , University of California , Davis , CA 95616 , USA; d Department of Biomedical Engineering , University of California , Davis , CA 95616 , USA

## Abstract

*O*-Sulfated sialyl Lewis *x* antigens play important roles in nature. However, due to their structural complexity, they are not readily accessible by either chemical or enzymatic synthetic processes. Taking advantage of a bacterial sialyltransferase mutant that can catalyze the transfer of different sialic acid forms from the corresponding sugar nucleotide donors to Lewis *x* antigens, which are fucosylated glycans, as well as an efficient one-pot multienzyme (OPME) sialylation system, *O*-sulfated sialyl Lewis *x* antigens containing different sialic acid forms and *O*-sulfation at different locations were systematically synthesized by chemoenzymatic methods.

## Abstract 2

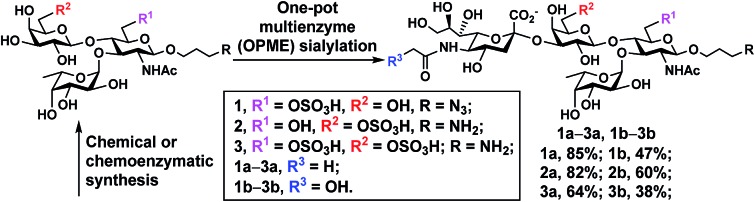
 *O*-Sulfated sialyl Lewis *x* antigens containing different sialic acid forms were chemoenzymatically synthesized using a bacterial sialyltransferase mutant.

## Introduction

**Fig. 1:**
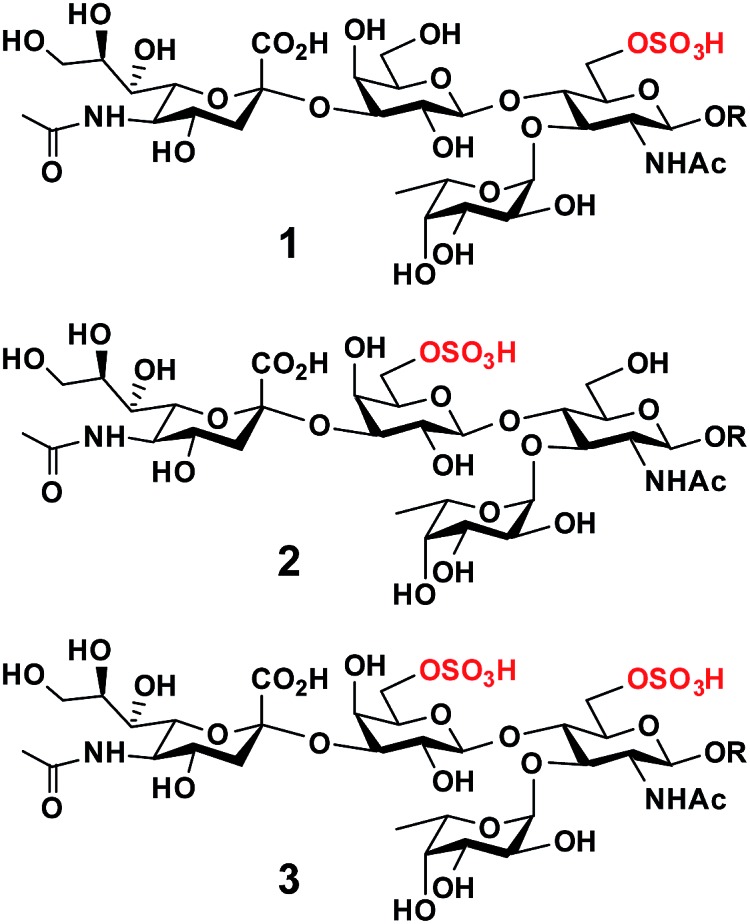
Structures of *O*-sulfated sialyl Lewis *x* including 6-*O*-sulfo-sLe^*x*^ (**1**), 6′-*O*-sulfo-sLe^*x*^ (**2**), and 6′, 6-di-*O*-sulfo-sLe^*x*^ (**3**).

*O*-Sulfated sialyl Lewis *x* structures play important roles in immune regulation, inflammation, and cancer metastasis.^1^ For example, 6-*O*-sulfo-sialyl Lewis *x* [6-*O*-sulfo-sLe^*x*^ (**1**), Neu5Acα2-3Galβ1-4(Fucα1-3)GlcNAc6SβOR] with an *O*-sulfate group at the carbon-6 of the *N*-acetylglucosamine (GlcNAc) residue (Fig. 1) is a well known ligand for L-selectin, a C-type (Ca^2+^-dependent) carbohydrate-binding protein (lectin) expressed broadly in most leukocytes in the blood.^1^,^2^ The interaction of 6-*O*-sulfo-sLe^*x*^ (**1**) and L-selectin plays a critical role in lymphocyte homing to the peripheral lymph nodes^2^ and in chronic inflammation.^3^ It has also been shown that human sialic acid-binding immunoglobulin-like lectin^4^ Siglec-9 binds strongly^5^,^6^ to 6-*O*-sulfo-sLe^*x*^, but the biological importance of this interaction is less well understood.

On the other hand, 6′-*O*-sulfo-sialyl Lewis *x* [6′-*O*-sulfo-sLe^*x*^ (**2**), Neu5Acα2-3Gal6Sβ1-4(Fucα1-3)GlcNAcβOR] with an *O*-sulfate group at the carbon-6 of the galactose (Gal) residue (Fig. 1),^7^ in addition to 6′-*O*-sulfo-sialyl-*N*-acetyllactosamine (6′-*O*-sulfo-sLacNAc, Neu5Acα2-3Gal6Sβ1-4GlcNAcβOR),^8^ was shown by glycan microarray studies to be a preferred glycan ligand for Siglec-8 and for its paralog mouse Siglec-F.^9^ Siglec-8 is expressed in human allergic inflammatory cells including eosinophils, mast cells, and basophils.^5^,^10^ Reducing the number of eosinophils, such as by soluble 6′-*O*-sulfo-sLe^*x*^ synthetic polymer induced apoptosis,^11^ has been suggested as an approach for asthma therapies.^12^ Furthermore, 6′-*O*-sulfo-sLe^*x*^ (**2**), in addition to 6′-*O*-sulfo-sLacNAc and 6′-*O*-sulfo-sialyl-lacto-*N*-neotetraose (6′-*O*-sulfo-sLNnT, Neu5Acα2-3Gal6Sβ1-4GlcNAcβ1-3Galβ1-4GlcβOR), was shown to bind to langerin,^13^ a C-type (Ca^2+^-dependent) lectin specific to Langerhans cells (immature antigen-presenting specific T cell immunity initiating dendritic cells of epidermis and mucosal tissues).^14^

**Scheme 1:**
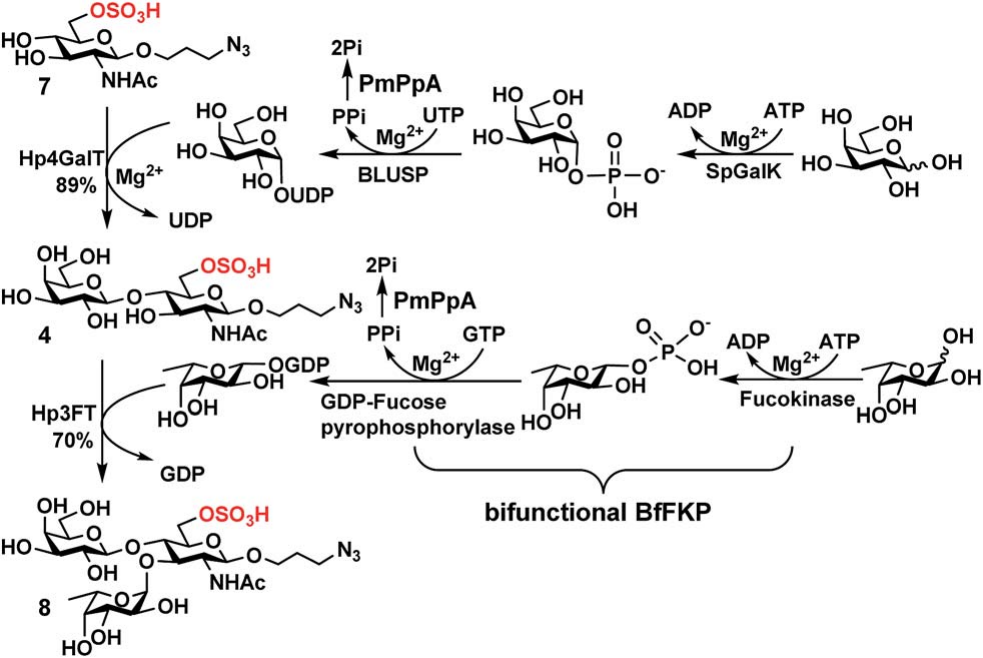
Sequential OPME synthesis of 6-*O*-sulfo-Le^*x*^βProN_3_ (**8**) from 6-*O*-sulfo-GlcNAcβProN_3_ (**7**) using an OPME β1-4-galactosyl activation and transfer system for the formation of 6-*O*-sulfo-LacNAcβProN_3_ (**4**) followed by an OPME α1-3-fucosyl activation and transfer system for the formation of 6-*O*-sulfo-Le^*x*^βProN_3_ (**8**). Enzymes and abbreviations: SpGalK, *Streptococcus pneumoniae* TIGR4 galactokinase;^44^ BLUSP, *Bifidobacterium longum* UDP-sugar pyrophosphorylase;^45^ PmPpA, *Pasteurella multocida* inorganic pyrophosphorylase;^43^ Hp4GalT, *Helicobacter pylori* β1-4-galactosyltransferase;^43^ BfFKP, *Bacteroides fragilis* bifunctional l-fucokinase/GDP-fucose pyrophosphorylase;^42^ and Hp3FT, *Helicobacter pylori* α1-3-fucosyltransferase.^39^,^41^

Although less efficient than Neu5Acα2-8Neu5Acα2-3LacNAc, both 6-*O*-sulfo-sLe^*x*^ (**1**) and 6′-*O*-sulfo-sLe^*x*^ (**2**) bound moderately to human Siglec-7.^5^ Both are present in glycosylation-dependent cell adhesion molecule 1 (GlyCAM-1), an L-selectin ligand,^15^ with 6′-*O*-sulfo-sLe^*x*^ (**2**) as the major sulfated form.^16^–^18^ Gal-6-*O*-sulfotransferase and GlcNAc-6-*O*-sulfotransferase have been found to synergistically produce L-selectin ligands. This indicates either the potential synergistic involvement of both 6-*O*-sulfo-sLe^*x*^ (**1**) and 6′-*O*-sulfo-sLe^*x*^ (**2**) or the involvement of 6′,6-di-*O*-sulfo-sLe^*x*^ (**3**) (Fig. 1) with *O*-sulfate groups at both the Gal and GlcNAc residues of sLe^*x*^ in L-selectin-binding.^19^ Human Siglec-7 and -8 have also been shown to bind more strongly to 6′,6-di-*O*-sulfo-sLe^*x*^ (**3**) than their mono-*O*-sulfated derivatives (**1** and **2**), while mouse Siglec-F has been shown to bind with similar strength to 6′,6-di-*O*-sulfo-sLe^*x*^ (**3**) and 6′-*O*-sulfo-sLe^*x*^ (**2**).^6^

The biological importance of *O*-sulfated sLe^*x*^ structures makes them attractive synthetic targets. However, the structures of these compounds are relatively complex and include synthetically challenging α2-3-linked sialic acid, which suffers from low stereoselectivity and a high 2,3-elimination rate in chemical synthesis,^20^–^22^ as well as the acid labile *O*-sulfate group.^23^,^24^ Chemically^20^,^25^,^26^ or chemoenzymatically^27^ synthesized Neu5Acα2-3Gal building blocks have been used as effective synthons for constructing more complex sialosides including sLe^*x*^ and 6-*O*-sulfo-sLe^*x*^ (**1**).^20^ Several examples of the chemical^22^,^28^ or chemoenzymatic^29^ synthesis of 6-*O*-sulfo-sLe^*x*^ (**1**) as well as the chemical synthesis of 6′-*O*-sulfo-sLe^*x*^ (**2**)^22^,^30^,^31^ and 6′,6-di-*O*-sulfo-sLe^*x*^ (**3**)^32^ have been reported. All these examples are, however, limited to compounds with the most abundant sialic acid form, *N*-acetylneuraminic acid (Neu5Ac). Despite the presence of more than 50 different sialic acid forms identified in nature,^33^,^34^ *O*-sulfated sLe^*x*^ containing a sialic acid form other than Neu5Ac has not been synthesized.

We report here the development of efficient chemoenzymatic methods for the systematic synthesis of *O*-sulfated sLe^*x*^ containing different sialic acid forms. The methods are demonstrated for representative examples of 6′-*O*-sulfo-sLe^*x*^ (**1**), 6-*O*-sulfo-sLe^*x*^ (**2**) and/or 6′,6-di-*O*-sulfo-sLe^*x*^ (**3**) containing the most abundant Neu5Ac form and *N*-glycolylneuraminic acid (Neu5Gc), a sialic acid form commonly found in mammals other than humans, but which can be incorporated into the human glycome from dietary sources.^35^

One efficient approach for the synthesis of *O*-sulfated sLe^*x*^ with different sialic acid forms would be by direct sialylation of *O*-sulfated Le^*x*^ using one-pot multienzyme (OPME) sialylation systems^36^ containing an α2-3-sialyltransferase and a CMP-sialic acid synthetase (CSS),^37^ with or without a sialic acid aldolase.^38^ Such an approach has been successfully demonstrated for direct sialylation of non-sulfated Le^*x*^ for the synthesis of sLe^*x*^ containing a diverse array of naturally occurring and non-natural sialic acid forms, using OPME systems containing a recombinant viral α2-3-sialyltransferase vST3Gal-I^39^ or a bacterial multifunctional sialyltransferase mutant, *Pasteurella multocida* α2-3-sialyltransferase 1 (PmST1) M144D.^40^ The latter, with a high expression level (98 mg L^-1^ culture, >1000-fold higher than that of vST3Gal-I) and high promiscuity in tolerating different modifications on the sialic acid in the substrates, is a superior choice for the synthesis.^40^ However, it is not clear whether *O*-sulfated Le^*x*^ structures could be used with PmST1 M144D as suitable acceptors in the OPME sialylation process to produce the desired *O*-sulfated sLe^*x*^ with different sialic acid forms.

## Results and discussion

### Synthesis of *O*-sulfated disaccharides and *O*-sulfated Le^*x*^

**Fig. 2:**
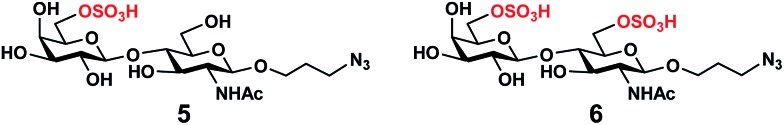
Structures of chemically synthesized 6′-*O*-sulfo-LacNAcβProN_3_ (**5**) and 6,6′-di-*O*-sulfo-LacNAcβProN_3_ (**6**).

In order to obtain *O*-sulfated Le^*x*^ as potential acceptor substrates for PmST1 M144D, enzyme-catalyzed α1-3-fucosylation of the corresponding *O*-sulfated disaccharides was tested as a potential strategy. A one-pot three-enzyme (OP3E) α1-3-fucosylation system (Scheme 1)^39^,^41^ containing *Bacteroides fragilis* bifunctional l-fucokinase/GDP-fucose pyrophosphorylase (BfFKP),^42^*Pasteurella multocida* inorganic pyrophosphorylase (PmPpA),^43^ and *Helicobacter pylori* α1-3-fucosyltransferase (Hp1-3FTΔ66 or Hp3FT) was used for this purpose. The *O*-sulfated disaccharides tested were 6-*O*-sulfo-LacNAcβProN_3_ (**4**) (Scheme 1), 6′-*O*-sulfo-LacNAcβProN_3_ (**5**), and 6,6′-di-*O*-sulfo-LacNAcβProN_3_ (**6**) (Fig. 2). LacNAcβProN_3_ (ref. 43) without any *O*-sulfate groups was used as a positive control.

6-*O*-Sulfo-LacNAcβProN_3_ (**4**) was synthesized from 6-*O*-sulfo-GlcNAcβProN_3_ (**7**)^43^ using an improved OPME galactosyl activation and transfer system (Scheme 1) containing *Streptococcus pneumoniae* TIGR4 galactokinase (SpGalK),^44^*Bifidobacterium longum* UDP-sugar pyrophosphorylase (BLUSP),^45^ PmPpA, and a *Helicobacter pylori* β1-4-galactosyltransferase (Hp1-4GalT or Hp4GalT).^43^ The EcGalK, BLUSP, and PmPpA allowed *in situ* formation of the donor substrate of Hp4GalT, uridine 5′-diphosphate-galactose (UDP-Gal), from monosaccharide galactose (Gal).^45^ It was previously shown that Hp4GalT, but not *Neisseria meningitidis* β1-4-galactosyltransferase (NmLgtB), was able to use 6-*O*-sulfated GlcNAc and derivatives as acceptor substrates for the synthesis of β1-4-linked galactosides.^43^ The activity of Hp4GalT in synthesizing 6-*O*-sulfo-LacNAcβProN_3_ (**4**) was confirmed again here using the improved OPME approach.^45^,^46^ An excellent 89% yield was obtained, comparing favourably to the previous Hp4GalT-dependent OPME β1-4-galactosylation approach (70% yield) which used *Escherichia coli* K-12 glucose-1-P uridylyltransferase (EcGalU), *Escherichia coli* UDP-galactose-4-epimerase (EcGalE), and PmPpA to produce UDP-Gal *in situ* from glucose-1-phosphate.^43^ 6′-*O*-Sulfo-LacNAcβProN_3_ (**5**) and 6,6′-di-*O*-sulfo-LacNAcβProN_3_ (**6**) (Fig. 2) were chemically synthesized (see ESI†).

**Scheme 2:**
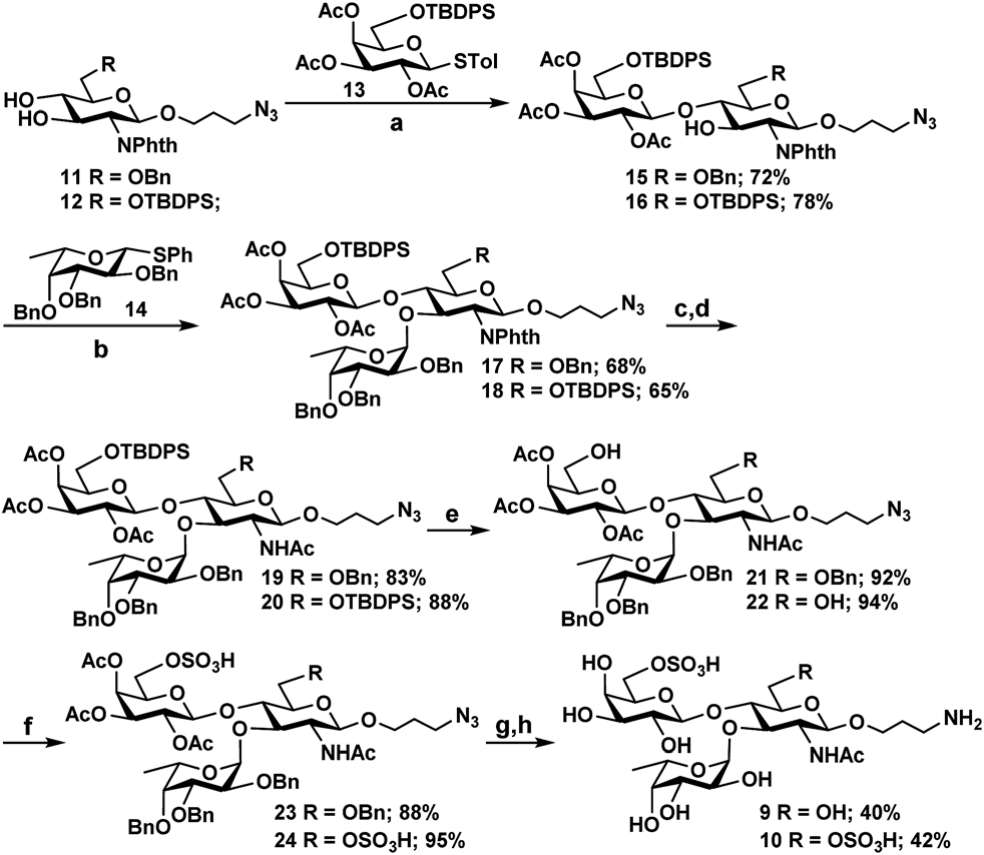
Chemical synthesis of 6′-*O*-sulfo-Le^*x*^βProNH_2_ (**9**) and 6,6′-di-*O*-sulfo-Le^*x*^βProNH_2_ (**10**). Reagents and conditions: (a) *N*-iodosuccinimide (NIS), TMSOTf, MS 4 Å, CH_2_Cl_2_, –40 °C, 30 min; (b) *N*-iodosuccinimide (NIS), TMSOTf, MS 4 Å, CH_2_Cl_2_–Et_2_O (1 : 1), –18 °C, 45 min; (c) H_2_N(CH_2_)_2_NH_2_, *n*-BuOH, 90 °C, 8 h; (d) pyridine, Ac_2_O, r.t., 10 h; (e) HF pyridine, 0 °C to r.t., overnight; (f) SO_3_ pyridine, pyridine, 0 °C to r.t.; (g) 0.1 M NaOMe, MeOH, r.t., 3 h; (h) Pd(OH)_2_/C, H_2_, CH_3_OH, 48 h.

Among the three *O*-sulfated disaccharides tested, only 6-*O*-sulfo-LacNAcβProN_3_ (**4**) was a suitable acceptor for Hp3FT to produce the desired 6-*O*-sulfo-Le^*x*^βProN_3_ (**8**). In contrast, 6′-*O*-sulfo-LacNAcβProN_3_ (**5**) and 6,6′-di-*O*-sulfo-LacNAcβProN_3_ (**6**) were not used efficiently by Hp3FT for the synthesis of the corresponding *O*-sulfated Le^*x*^ derivatives. With the positive outcome in small scale reactions for fucosylation of 6-*O*-sulfo-LacNAcβProN_3_ (**4**), the preparative-scale synthesis of 6-*O*-sulfo-Le^*x*^βProN_3_ (**8**) was carried out using the OP3E α1-3-fucosyl activation and transfer system (Scheme 1). A yield of 70% was obtained. The combined sequential OPME β1-4-galactosylation and OPME α1-3-fucosylation (Scheme 1) was an effective approach for obtaining 6-*O*-sulfo-Le^*x*^βProN_3_ (**8**) from a simple monosaccharide derivative 6-*O*-sulfo-GlcNAcβProN_3_ (**7**) in an overall yield of 62%.

As Hp3FT was not able to use 6′-*O*-sulfo-LacNAcβProN_3_ (**5**) or 6,6′-di-*O*-sulfo-LacNAcβProN_3_ (**6**) efficiently as acceptors for fucosylation to obtain the desired Le^*x*^ trisaccharides, the target trisaccharides 6′-*O*-sulfo-Le^*x*^βProNH_2_ (**9**) and 6,6′-di-*O*-sulfo-Le^*x*^βProNH_2_ (**10**) were chemically synthesized (Scheme 2) from monosaccharide synthons **11**, **12**,^27^**13**, and **14**.^27^ Notable features of the synthetic strategy include: (a) application of an efficient general protection strategy^47^ for the synthesis of the two trisaccharides (*i.e.* similar protecting groups were used in the syntheses and the same reagents were used for their removal); (b) use of similar thioglycoside derivatives as glycosyl donors in all glycosylations; (c) high regio- and stereoselectivity in product formation; (d) one step removal of benzyl ethers and reduction of the azido group using 20% Pd(OH)_2_/C (Pearlman's catalyst) and H_2_.^48^ More specifically, for the synthesis of **9** and **10**, two *N*-phthalimide glucosamine derivatives **11** and **12** selectively protected at C6 with benzyl and *tert*-butyldiphenylsilyl ether (TBDPS), respectively, were coupled stereoselectively with thioglycoside donor **13**, which was selectively protected with TBDPS at C6, in the presence of *N*-iodosuccinimide (NIS) and trimethylsilyl trifluoromethanesulfonate (TMSOTf)^49^ in dichloromethane. Disaccharide derivatives **15** and **16** were obtained in 72% and 78% yields, respectively. The bulky *N*-phthalimido protecting group in acceptors **11** and **12** provides steric hindrance to the neighboring C-3 hydroxyl group and decreases the reactivity of the C-3 hydroxyl group. Therefore, glycosylation occurs regioselectively at the C-4 hydroxyl group.^27^ Initial attempts to glycosylate acceptors **15** and **16** in dichloromethane with 1.2 equivalents of thiophenyl fucoside **14** produced trisaccharides in alpha and beta mixtures. In contrast, stereospecific formation of trisaccharides was achieved when a mixed solvent of diethylether and dichloromethane (1 : 1)^50^,^51^ was employed. The reaction of acceptors **15** and **16** with 1.2 equivalents of fucosyl donor **14** produced compounds **17** and **18** in 68% and 65% yields, respectively. Compounds **17** and **18** were then subjected to a series of synthetic transformations: (a) conversion of the *N*-phthaloyl group to an acetamido group by removing the phthaloyl group using ethylenediamine, followed by *N*- and *O*-acetylation using acetic anhydride and pyridine; (b) HF-pyridine-mediated selective removal of the TBDPS group;^52^ (c) *O*-sulfation of the primary hydroxyl group by SO_3_ pyridine complex;^52^,^53^ (d) deacetylation by NaOMe in MeOH;^54^ and (e) hydrogenation using Pd(OH)_2_/C and H_2_ (ref. 55) to obtain the desired 6′-*O*-sulfo-Le^*x*^βProNH_2_ (**9**) and 6,6′-di-*O*-sulfo-Le^*x*^βProNH_2_ (**10**).

### Enzymatic synthesis of *O*-sulfated sLe^*x*^

**Scheme 3:**
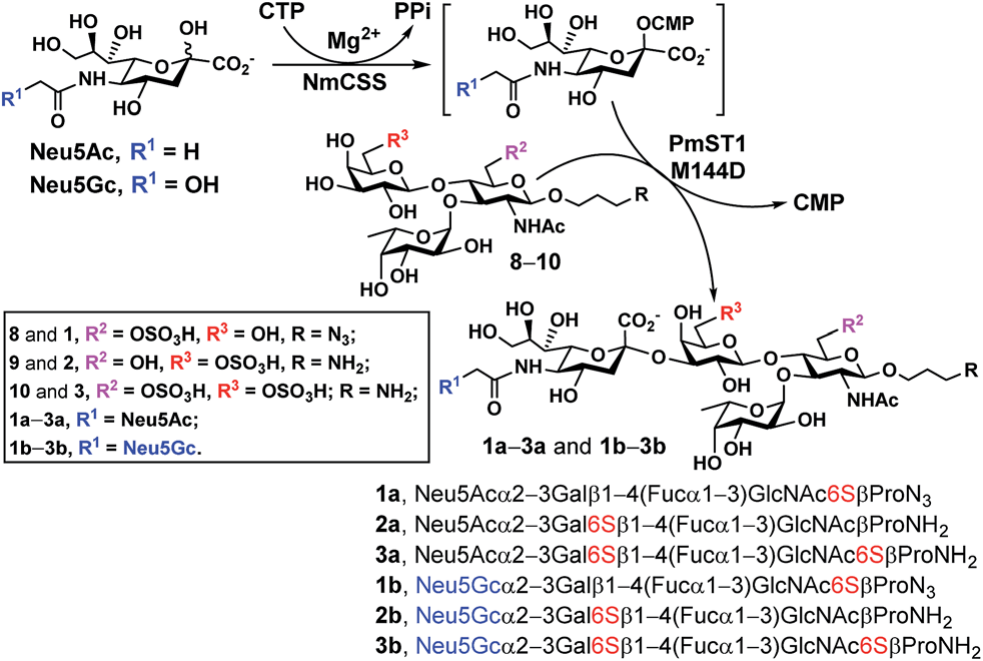
PmST1 M144D-mediated one-pot two-enzyme (OP2E) sialylation of *O*-sulfo analogues of Lewis^*x*^. Yields obtained for *O*-sulfated sLe^*x*^ tetrasaccharides: **1a**, 85%; **1b**, 47%; **2a**, 82%; **2b**, 60%; **3a**, 64%; **3b**, 38%. Enzymes and abbreviations: NmCSS, *Neisseria meningitidis* CMP-sialic acid;^37^ PmST1 M144D, *Pasteurella multocida* α2-3-sialyltransferase 1 (PmST1) M144D mutant.^40^

With chemoenzymatically synthesized 6-*O*-sulfo-Le^*x*^βProN_3_ (**8**) as well as chemically synthesized 6′-*O*-sulfo-Le^*x*^βProNH_2_ (**9**) and 6,6′-di-*O*-sulfo-Le^*x*^βProNH_2_ (**10**) in hand, a one-pot two-enzyme (OP2E) sialylation system (Scheme 3) was used to test the tolerance of PmST1 M144D^40^ for using these *O*-sulfated Le^*x*^ compounds as potential acceptor substrates. PmST1 M144D was previously engineered by protein crystal structure-assisted design. It has 20-fold reduced CMP-sialic acid (donor) hydrolysis activity and significantly (5588-fold) decreased α2-3-sialidase activity compared to the wild-type enzyme. It was used efficiently in a one-pot three-enzyme (OP3E) sialylation system for the synthesis of non-sulfated sLe^*x*^ tetrasaccharides containing diverse sialic acid forms from Le^*x*^.^40^ To our delight, PmST1 M144D also tolerated *O*-sulfated Le^*x*^ containing *O*-sulfate at C-6, C-6′, or both. In addition to *N*-acetylneuraminic acid (Neu5Ac), *N*-acetylneuraminic acid (Neu5Gc) was also successfully introduced to compounds **8–10**. *O*-Sulfated sLe^*x*^ tetrasaccharides 6-*O*-sulfo-Neu5Acα2-3Le^*x*^βProN_3_ (**1a**, 80 mg, 85%), 6-*O*-sulfo-Neu5Gcα2-3Le^*x*^βProN_3_ (**1b**, 22 mg, 47%), 6′-*O*-sulfo-Neu5Acα2-3Le^*x*^βProNH_2_ (**2a**, 75 mg, 82%), 6′-*O*-sulfo-Neu5Acα2-3Le^*x*^βProNH_2_ (**2b**, 45 mg, 60%), 6,6′-di-*O*-sulfo-Neu5Acα2-3Le^*x*^βProNH_2_ (**3a**, 42 mg, 64%), and 6,6′-di-*O*-sulfo-Neu5Gcα2-3Le^*x*^βProNH_2_ (**3b**, 40 mg, 38%) were successfully obtained using this highly efficient one-pot two-enzyme system containing *Neisseria meningitidis* CMP-sialic acid (NmCSS)^37^ and PmST1 M144D^40^ from the corresponding acceptors **8–10** and Neu5Ac or Neu5Gc, respectively. In general, Neu5Gc was used less efficiently by the OPME sialylation system, leading to lower yields for **1b–3b** (38–60%) compared to their Neu5Ac-counterparts **1a–3a** (64–85%). *O*-Sulfated sLe^*x*^ glycans with a propyl amine aglycone (compounds **2a**, **3a**, **2b** and **3b**) were found to be more challenging for column purification compared to the ones with a propyl azide aglycone (compounds **1a** and **1b**). When a desired sialic acid is readily available such as in the case presented here, a one-pot two-enzyme (OP2E) system is sufficient. When only the 6-carbon precursors of the desired sialic acid forms are available, the one-pot three-enzyme (OP3E) sialylation system including an aldolase in addition to NmCSS and PmST1 M144D^40^ should be used.

## Conclusions

In conclusion, we have successfully developed an efficient chemoenzymatic method for the systematic synthesis of synthetically challenging *O*-sulfated sLe^*x*^ (**1a–3a** and **1b–3b**) containing different sialic acid forms (Neu5Ac or Neu5Gc) by direct sialylation of the corresponding *O*-sulfated Le^*x*^ structures **8–10** using an efficient one-pot two-enzyme (OP2E) system containing NmCSS and PmST1 M144D. The method can be extended to the synthesis of *O*-sulfated sLe^*x*^ structures containing other sialic acid forms. We have also shown here that a relatively complex trisaccharide 6-*O*-sulfo-Le^*x*^βProN_3_ (**8**) can be efficiently produced from a simple monosaccharide derivative 6-*O*-sulfo-GlcNAcβProN_3_ (**7**) by a sequential OPME β1-4-galactosylation and OPME α1-3-fucosylation process. PmST1 M144D has been demonstrated to be a powerful catalyst not only for synthesizing non-sulfated sLe^*x*^ structures as shown previously,^40^ but also for producing biologically important but difficult-to-obtain *O*-sulfated sLe^*x*^.

## Supplementary Material

Supplementary information

## References

[cit1] Rosen S. D. (2004). Annu. Rev. Immunol..

[cit2] Kawashima H., Fukuda M. (2012). Ann. N. Y. Acad. Sci..

[cit3] Kobayashi M., Lee H., Nakayama J., Fukuda M. (2009). Curr. Drug Metab..

[cit4] Macauley M. S., Crocker P. R., Paulson J. C. (2014). Nat. Rev. Immunol..

[cit5] Kiwamoto T., Kawasaki N., Paulson J. C., Bochner B. S. (2012). Pharmacol. Ther..

[cit6] Campanero-Rhodes M. A., Childs R. A., Kiso M., Komba S., le Narvor C., Warren J., Otto D., Crocker P. R., Feizi T. (2006). Biochem. Biophys. Res. Commun..

[cit7] Bochner B. S., Alvarez R. A., Mehta P., Bovin N. V., Blixt O., White J. R., Schnaar R. L. (2005). J. Biol. Chem..

[cit8] Kiwamoto T., Brummet M. E., Wu F., Motari M. G., Smith D. F., Schnaar R. L., Zhu Z., Bochner B. S. (2014). J. Allergy Clin. Immunol..

[cit9] Tateno H., Crocker P. R., Paulson J. C. (2005). Glycobiology.

[cit10] Bochner B. S. (2009). Clin. Exp. Allergy.

[cit11] Hudson S. A., Bovin N. V., Schnaar R. L., Crocker P. R., Bochner B. S. (2009). J. Pharmacol. Exp. Ther..

[cit12] Kiwamoto T., Katoh T., Evans C. M., Janssen W. J., Brummet M. E., Hudson S. A., Zhu Z., Tiemeyer M., Bochner B. S. (2015). J. Allergy Clin. Immunol..

[cit13] Galustian C., Park C. G., Chai W., Kiso M., Bruening S. A., Kang Y. S., Steinman R. M., Feizi T. (2004). Int. Immunol..

[cit14] Valladeau J., Ravel O., Dezutter-Dambuyant C., Moore K., Kleijmeer M., Liu Y., Duvert-Frances V., Vincent C., Schmitt D., Davoust J., Caux C., Lebecque S., Saeland S. (2000). Immunity.

[cit15] Lasky L. A., Singer M. S., Dowbenko D., Imai Y., Henzel W. J., Grimley C., Fennie C., Gillett N., Watson S. R., Rosen S. D. (1992). Cell.

[cit16] Hemmerich S., Rosen S. D. (1994). Biochemistry.

[cit17] Hemmerich S., Leffler H., Rosen S. D. (1995). J. Biol. Chem..

[cit18] Hemmerich S., Bertozzi C. R., Leffler H., Rosen S. D. (1994). Biochemistry.

[cit19] Bistrup A., Bhakta S., Lee J. K., Belov Y. Y., Gunn M. D., Zuo F. R., Huang C. C., Kannagi R., Rosen S. D., Hemmerich S. (1999). J. Cell Biol..

[cit20] Pazynina G. V., Sablina M. A., Tuzikov A. B., Chinarev A. A., Bovin N. V. (2003). Mendeleev Commun..

[cit21] Lai C. H., Hahm H. S., Liang C. F., Seeberger P. H. (2015). Beilstein J. Org. Chem..

[cit22] Misra A. K., Ding Y., Lowe J. B., Hindsgaul O. (2000). Bioorg. Med. Chem. Lett..

[cit23] Liang A., Thakkar J. N., Desai U. R. (2010). J. Pharm. Sci..

[cit24] Al-Horani R. A., Desai U. R. (2010). Tetrahedron.

[cit25] Krock L., Esposito D., Castagner B., Wang C.-C., Bindschadler P., Seeberger P. H. (2012). Chem. Sci..

[cit26] Esposito D., Hurevich M., Castagner B., Wang C. C., Seeberger P. H. (2012). Beilstein J. Org. Chem..

[cit27] Cao H., Huang S., Cheng J., Li Y., Muthana S., Son B., Chen X. (2008). Carbohydr. Res..

[cit28] Komba S., Galustian C., Ishida H., Feizi T., Kannagi R., Kiso M. (1999). Angew. Chem., Int. Ed..

[cit29] Pratt M. R., Bertozzi C. R. (2004). Org. Lett..

[cit30] Komba S., Ishida H., Kiso M., Hasegawa A. (1996). Bioorg. Med. Chem..

[cit31] Jain R. K., Vig R., Rampal R., Chandrasekaran E. V., Matta K. L. (1994). J. Am. Chem. Soc..

[cit32] Komba S., Ishida H., Kiso M., Hasegawa A. (1996). Carbohydr. Res..

[cit33] Chen X., Varki A. (2010). ACS Chem. Biol..

[cit34] Angata T., Varki A. (2002). Chem. Rev..

[cit35] Varki A. (2001). Am. J. Phys. Anthropol..

[cit36] Yu H., Chokhawala H. A., Huang S., Chen X. (2006). Nat. Protoc..

[cit37] Yu H., Yu H., Karpel R., Chen X. (2004). Bioorg. Med. Chem..

[cit38] Li Y., Yu H., Cao H., Lau K., Muthana S., Tiwari V. K., Son B., Chen X. (2008). Appl. Microbiol. Biotechnol..

[cit39] Sugiarto G., Lau K., Yu H., Vuong S., Thon V., Li Y., Huang S., Chen X. (2011). Glycobiology.

[cit40] Sugiarto G., Lau K., Qu J., Li Y., Lim S., Mu S., Ames J. B., Fisher A. J., Chen X. (2012). ACS Chem. Biol..

[cit41] Yu H., Lau K., Li Y., Sugiarto G., Chen X. (2012). Current Protocols in Chemical Biology.

[cit42] Yi W., Liu X., Li Y., Li J., Xia C., Zhou G., Zhang W., Zhao W., Chen X., Wang P. G. (2009). Proc. Natl. Acad. Sci. U. S. A..

[cit43] Lau K., Thon V., Yu H., Ding L., Chen Y., Muthana M. M., Wong D., Huang R., Chen X. (2010). Chem. Commun..

[cit44] Chen M., Chen L. L., Zou Y., Xue M., Liang M., Jin L., Guan W. Y., Shen J., Wang W., Wang L., Liu J., Wang P. G. (2011). Carbohydr. Res..

[cit45] Chen X., Fang J. W., Zhang J. B., Liu Z. Y., Shao J., Kowal P., Andreana P., Wang P. G. (2001). J. Am. Chem. Soc..

[cit46] Yu H., Lau K., Thon V., Autran C. A., Jantscher-Krenn E., Xue M., Li Y., Sugiarto G., Qu J., Mu S., Ding L., Bode L., Chen X. (2014). Angew. Chem., Int. Ed..

[cit47] Miranda L. P., Meldal M. (2001). Angew. Chem..

[cit48] Corey E. J., Link J. O. (1992). J. Am. Chem. Soc..

[cit49] Veeneman G. H., van Leeuwen S. H., van Boom J. H. (1990). Tetrahedron Lett..

[cit50] Ghosh T., Santra A., Misra A. K. (2014). RSC Adv..

[cit51] Si A., Misra A. K. (2015). ChemistryOpen.

[cit52] Yang B., Yoshida K., Yin Z., Dai H., Kavunja H., El-Dakdouki M. H., Sungsuwan S., Dulaney S. B., Huang X. (2012). Angew. Chem., Int. Ed..

[cit53] Santra A., Guchhait G., Misra A. K. (2011). Green Chem..

[cit54] Wang Z., in Comprehensive Organic Name Reactions and Reagents, John Wiley & Sons, Inc., 2010.

[cit55] Pearlman W. M. (1967). Tetrahedron Lett..

