# Supplementary material for: Systematic chemoenzymatic synthesis of O-sulfated sialyl Lewis x antigens
Source: Chem Sci. 2015 Dec 17;7(4):2827–31. doi: 10.1039/c5sc04104j (PMC5269574; doi:10.1039/c5sc04104j)

Electronic Supplementary Material (ESI) for Chemical Science.
This journal is © The Royal Society of Chemistry 2015

Supporting Information

for

Systematic Chemoenzymatic Synthesis of *O*-Sulfated Sialyl Lewis x Antigens

Abhishek Santra[a], Hai Yu[a], Nova Tasnima[a], Musleh M. Muthana[a,b], Yanhong Li[a], Jie Zeng[a,c], Nicholas J. Kenyon[d], Angelique Y. Louie[e], and Xi Chen[a,]*

[a.] *Department of Chemistry, University of California, Davis One Shields Avenue, Davis, CA 95616 (USA) E-mail: xiichen@ucdavis.edu*
[b.] *Current address: Children's National Medical Center, Washington DC, 20010 (USA)*
[c.] *School of Food Science, Henan Institute of Science and Technology, Xinxiang, 453003 (China)*
[d.] *Division of Pulmonary, Critical Care and Sleep Medicine, Department of Internal Medicine, University of California, Davis, CA 95616 (USA)*
[e.] *Department of Biomedical Engineering, University of California, Davis, CA 95616 (USA)*
**Corresponding author. Mailing address: Department of Chemistry, One Shields Avenue, Davis, CA 95616, USA. Phone: 530-754-6037. Fax: 530-752-8995. E-mail: xiichen@ucdavis.edu*

Table of Contents

General methods for compound purification and characterization ...……......…..…............…......S2
Chemical synthesis of *O*-sulfated LacNAc (**5**–**6**), and *O*-sulfated Lewis x glycans (**9**–**10**).....S2–S16
Sequential one-pot multienzyme (OPME) synthesis of 6-*O*-sulfo-LacNAcβProN_3 (**4**) and 6-*O*-sulfo-Le^{x}βProN_3 (**8**)..S17–S18
One-pot two-enzyme synthesis of *O*-sulfated sLe^x tetrasaccharides **1a**–**3a** and **1b**–**3b**.........S18–S20
References……...…………………………………………………………………………….....S20
^{1}H and ^{13}C NMR spectra of 6-*O*-sulfo-LacNAcβProN_3 (**4**)..S21
^{1}H and ^{13}C NMR spectra of 6'-*O*-sulfo-LacNAcβProN_3 (**5**)..S22
^{1}H and ^{13}C NMR spectra of 6',6-di-*O*-sulfo-LacNAcβProN_3 (**6**)...S23
^{1}H and ^{13}CNMR spectra of 6-*O*-sulfo-Le^{x}βProN_3 (**8**)..S24
^{1}H and ^{13}C NMR spectra of 6'-*O*-sulfo-Le^{x}βProNH_2 (**9**)……………………………………........S25
^{1}H and ^{13}C NMR spectra of 6, 6'-di-*O*-sulfo-Le^{x}βProNH_2 (**10**)..S26
^{1}H and ^{13}C NMR spectra of 6-*O*-sulfo-Neu5Acα2–3Le^{x}βProN_3 (**1a**) S27
^{1}H and ^{13}C NMR spectra of 6-*O*-sulfo-Neu5Gcα2–3Le^{x}βProN_3 (**1b**)..S28
^{1}H and ^{13}C NMR spectra of 6'-*O*-sulfo-Neu5Acα2–3Le^{x}βProNH_2 (**2a**).......................................S29
^{1}H and ^{13}C NMR spectra of 6'-*O*-sulfo-Neu5Gcα2–3Le^{x}βProNH_2 (**2b**).......................................S30
^{1}H and ^{13}C NMR spectra of 6,6'-di-*O*-sulfo-Neu5Acα2–3Le^{x}βProNH_2 (**3a**)................................S31
^{1}H and ^{13}C NMR spectra of 6,6'-di-*O*-sulfo-Neu5Gcα2–3Le^{x}βProNH_2 (**3b**)...............................S32

General methods for compound purification and characterization

Chemicals were purchased and used without further purification. ^{1}H NMR and ^{13}C NMR spectra were recorded on 300 MHz Mercury, 400 MHz Varian, 600 MHz VNMRS, or 800 MHz Bruker Avance III spectrometers. High resolution electrospray ionization (ESI) mass spectra were obtained using Thermo Electron LTQ-Orbitrap Hybrid MS at the Mass Spectrometry Facility in the University of California, Davis. Silica gel 60 Å (230–400 mesh, Sorbent Technologies) was used for flash column chromatography. Thin-layer chromatography (TLC, Sorbent Technologies) was performed on silica gel plates using anisaldehyde sugar staining or 5% sulfuric acid in ethanol staining for detection. Gel filtration chromatography was performed with a column (100 cm × 2.5 cm) packed with Bio-Gel P-2 Fine resins (Bio-Rad). Neu5Ac was bought from Atomole Scientific and CTP was from Chemfun Medical Technology Co. D-Galactose and D-glucosamine were purchased from Fisher Scientific (Pittsburgh, Pennsylvania, USA). L-Fucose was bought from V-LABS (Covington, Louisiana, USA). Guanidine 5'-triphosphate (GTP) was bought from Hangzhou Meiya Pharmacy (Hangzhou, China). Adenosine 5'-triphosphate (ATP) was bought from Beta Pharma Scientific, Inc (Branford, CT). Neu5Gc was chemoenzymatically synthesized as described previously.[1] Recombinant enzymes *Bacteroides fragilis* strain NCTC9343 bifunctional fucokinase/GDP-fucose pyrophosphorylase (FKP),[2] *Pasteurella multocida* inorganic pyrophosphatase (PmPpA),[3] α1–3-Fucosyltransferase from *Helicobacter pylori* (Hp3FT),[4] *Neisseria meningitidis* CMP-sialic acid synthetase (NmCSS),[1] *Pasteurella multocida* multifunctional α2–3-sialyltransferase 1 M144D mutant (PmST1 M144D)[5] were expressed and purified as described previously.

Chemical synthesis of *O*-sulfated LacNAc (5–6), and *O*-sulfated Lewis x glycans (9–10)

3-Chloropropyl 3,4,6-tri-*O*-acetyl-2-deoxy-2-phthalimido-β-D-glucopyranoside (S2)[6]

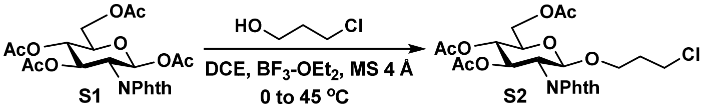

A mixture of 1,3,4,6-tetra-*O*-acetyl-2-deoxy-2-phthalimido-β-D-glucopyranoside **S1** (9.0 g, 18.86 mmol), 3-chloropropanol[6] (3.1 mL, 37.72 mmol), and 4 Å molecular sieves (5.0 g) in dry dichloroethane (DCE, 125 mL) was stirred at room temperature for 30 min under argon. The reaction mixture was cooled down to 0 °C and $BF_3{\cdot}OEt_2$ (4.6 mL, 37.72 mmol) was added. After 1 h, the reaction mixture was warmed to 45 °C and stirred for another 12 h. The reaction mixture was then diluted with CH_2Cl_2 (100 mL), washed sequentially with H_2O and a saturated aqueous solution of $NaHCO_3$, dried over Na_2SO_4, evaporated to dryness, and purified by silica gel chromatography using hexane:EtOAc = 1:1 (by volume) as an eluent to obtain glycoside **S2** as a solid foam (8.2 g, 86%). ^{1}H NMR (300 MHz, $CDCl_3$) δ 7.85 (dd, *J* = 5.7, 3.0 Hz, 2H), 7.74 (dd, *J* = 5.7, 2.7 Hz, 2H), 5.80 (dd, *J* = 10.5, 9.0 Hz, 1H), 5.37 (d, *J* = 8.7 Hz, 1H), 5.16 (dd, *J* = 9.9, 9.0 Hz, 1H), 4.36–4.27 (m, 2H), 4.17 (dd, *J* = 12.3, 2.4 Hz, 1H), 3.98–3.85 (m, 2H), 3.67–3.59 (m, 1H), 3.44–3.30 (m, 2H), 2.11, 2.03, 1.86 (s, 3H for each), 1.96–1.78 (m, 2H). ^{13}C NMR (75 MHz, $CDCl_3$) δ 170.89, 170.31, 169.64, 134.50 (2C), 131.50 (2C), 123.77 (2C), 98.50, 72.03, 70.84, 69.10, 66.63, 62.16, 54.74, 41.35, 32.19, 20.93, 20.79, 20.62.

3-Azidopropyl 3,4,6-tri-*O*-acetyl-2-deoxy-2-phthalimido-β-D-glucopyranoside (S3)[6]

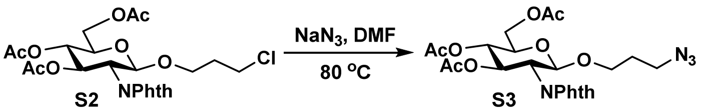

To a solution of **S2** (8 g, 15.65 mmol) in dry DMF (80 mL), NaN_3 (10.17 g, 156.52 mmol) was added. After stirring at 80 °C for overnight, the reaction mixture was concentrated to one-third of its original volume and diluted with EtOAc (150 mL), washed sequentially with H_2O and brine, dried over Na_2SO_4, evaporated to dryness, and purified by silica gel chromatography using hexane:EtOAc = 1:1 (by volume) as an eluent to obtain 3-azidopropyl 3,4,6-tri-*O*-acetyl-2-deoxy-2-phthalimido-β-D-glucopyranoside **S3** (7.5 g, 92%) as a white solid. ^{1}H NMR (300 MHz, $CDCl_3$) δ 7.86 (dd, *J* = 6.0, 3.3 Hz, 2H), 7.74 (dd, *J* = 5.1, 2.7 Hz, 2H), 5.78 (dd, *J* = 10.8, 9.0 Hz, 1H), 5.37 (d, *J* = 8.4 Hz, 1H), 5.17 (dd, *J* = 10.2, 9.0 Hz, 1H), 4.35–4.28 (m, 2H), 4.18 (dd, *J* = 12.3, 2.1 Hz, 1H), 3.94–3.84 (m, 2H), 3.58–3.51 (m, 1H), 3.24–3.10 (m, 2H), 2.11, 2.03, 1.86 (s, 3H for each), 1.81–1.63 (m, 2H). ^{13}C NMR (75 MHz, $CDCl_3$) δ 170.98, 170.42, 169.73, 134.64, 123.89, 98.41, 72.15, 70.96, 69.17, 66.83, 62.22, 54.80, 48.16, 29.01, 21.01, 20.88, 20.70.

3-Azidopropyl 4,6-*O*-benzilidine-2-deoxy-2-phthalimido-β-D-glucopyranoside (S5)

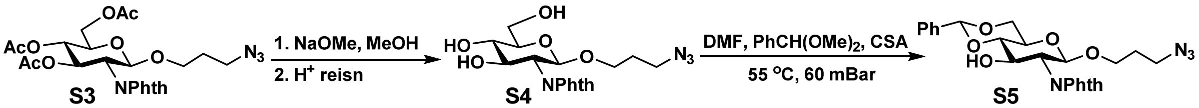

To a solution of **S3** (4.8 g, 9.2 mmol) in dry MeOH (100 mL), NaOMe (200 mg) was added. After stirring for 2 h at room temperature, the reaction mixture was neutralized with Dowex 50W (H^+), filtered and concentrated under reduced pressure to obtain glycoside **S4** as white solid. This intermediate was used in the next step without further purification.

To a solution of glycoside **S4** (3.6 g, 9.2 mmol) in dry DMF (10 mL), benzaldehyde dimethyl acetal (1.8 mL, 12.00 mmol) and camphor sulfonic acid (375 mg) were added. The reaction mixture was stirred at 55 °C and 60 mBar for 2 h. It was then quenched with Et_3N (1.5 mL) and the solvent was removed under reduced pressure to obtain a crude product. The crude product was diluted with CH_2Cl_2 (100 mL). The organic layer was washed with a saturated aqueous solution of $NaHCO_3$, dried over Na_2SO_4, concentrated and purified by silica gel chromatography using hexane:EtOAc = 2:1 (by volume) as an eluent to obtain compound **S5** (4.0 g, 91% in two steps) as a yellowish foam. ^{1}H NMR (800 MHz, $CDCl_3$) δ 7.87 (d, *J* = 2.4 Hz, 2H), 7.77–7.72 (m, 2H), 7.52–7.48 (m, 2H), 7.38 (m, 3H), 5.58 (s, 1H), 5.28 (d, *J* = 8.5 Hz, 1H), 4.64 (dd, *J* = 16.9, 6.8 Hz, 1H), 4.40 (dd, *J* = 10.6, 4.8 Hz, 1H), 4.27–4.22 (m, 1H), 3.93–3.87 (m, 1H), 3.86–3.82 (m, 1H), 3.66 (td, *J* = 9.5, 4.9 Hz, 1H), 3.62 (t, *J* = 9.1 Hz each, 1H), 3.57–3.50 (m, 1H), 3.24–3.13 (m, 2H), 1.78–1.64 (m, 2H). ^{13}C NMR (200 MHz, $CDCl_3$) δ 169.42, 169.35, 136.94, 134.29 (2C), 131.60 (2C), 129.44, 128.45 (3C), 126.33 (3C), 102.03, 98.94, 82.26, 68.71, 68.63, 66.46, 66.19, 56.50, 47.93, 28.87. HRMS (ESI) *m/z* calculated for $C_{24}H_{24}N_4O_7Na$ (M+Na) 503.1537, found 503.1530.

3-Azidopropyl 6-*O*-benzyl-2-deoxy-2-phthalimido-β-D-glucopyranoside (11)

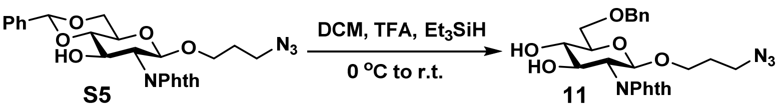

A solution of compound **S5** (3.5 g, 7.28 mmol) and Et_3SiH (7 mL, 43.68 mmol) in dry CH_2Cl_2 (30 mL) was cooled in an ice-water bath. TFA (3.3 mL, 43.68 mmol) was then added drop-wisely.[7] After stirring at room temperature for 3 h under argon, the mixture was concentrated and co-evaporated with toluene at room temperature. The residue was purified by silica gel chromatography using hexane:EtOAc = 1:1.5 (by volume) as an eluent to obtain compound **11** (2.9 g, 83%). ^{1}H NMR (800 MHz, $CDCl_3$) δ 7.81–7.68 (m, 4H), 7.37–7.32 (m, 4H), 7.31–7.27 (m, 1H), 5.18 (d, *J* = 8.4 Hz, 1H), 4.63 (d, *J* = 12.0 Hz, 1H), 4.58 (d, *J* = 12.0 Hz, 1H), 4.33–4.28 (m, 1H),

4.11 (dd, J = 10.9, 8.5 Hz, 1H), 3.89–3.82 (m, 1H), 3.82–3.73 (m, 2H), 3.65–3.56 (m, 2H), 3.54–3.45 (m, 1H), 3.19–3.09 (m, 2H), 1.77–1.60 (m, 2H). ^{13}C NMR (200 MHz, $CDCl_3$) δ 168.44 (2C), 137.66, 134.22, 134.10 (2C), 131.61 (2C), 128.56, 127.95 (2C), 127.92, 127.84, 123.49, 98.35, 73.90, 73.76, 73.75, 71.64, 70.30, 66.18, 56.26, 48.03, 28.86. HRMS (ESI) *m/z* calculated for $C_{24}H_{26}N_4O_7Na$ (M+Na) 505.1693, found 505.1687.

3-Azidopropyl 6-*tert*-*O*-butyldiphenylsilyl-2-deoxy-2-phthalimido-β-D-glucopyranoside (12)[8]

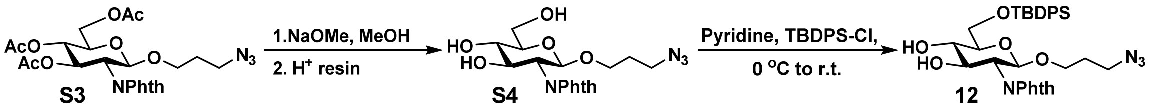

To a solution of **S3** (2.5 g, 4.8 mmol) in dry MeOH (30 mL), NaOMe (80 mg) was added. After stirring for 2 h at room temperature, the reaction mixture was neutralized with Dowex 50W (H^+), filtered and concentrated under reduced pressure to obtain glycoside **S4** as white solid. This intermediate was used in the next step without further purification.

To an ice cold solution of glycoside **S4** (1.8 g, 4.6 mmol) in dry pyridine (30 mL), TBDPSCl (1.4 mL, 5.52 mmol) was added drop-wisely.[8] After the addition was completed, the reaction mixture was stirred for overnight at room temperature before it was concentrated and purified by silica gel chromatography using hexane:EtOAc = 2:1 (by volume) as an eluent to obtain compound **12** as a syrup (2.5 g, 87% in two steps). ^{1}H NMR (600 MHz, $CDCl_3$) δ 7.81 (dd, 2H, J = 6.0, 3.6 Hz), 7.70–7.68 (m, 6H), 7.45–7.38 (m, 6H), 5.18 (d, J = 8.4 Hz, 1H), 4.34 (dd, J = 10.8, 8.4 Hz, 1H), 4.09 (dd, J = 10.8, 9.0 Hz, 1H), 3.94 (dd, J = 4.8, 1.8 Hz, 2H), 3.81–3.78 (m, 1H), 3.66 (t, J = 9.0 Hz each, 1H), 3.59–3.56 (m, 1H), 3.47–3.44 (m, 1H), 3.12 (dt, J = 6.0, 1.8 Hz, 2H), 1.73–1.60 (m, 2H), 1.06 (s, 9H). ^{13}C NMR (150 MHz, $CDCl_3$) δ 168.67, 168.59, 135.88 (2C), 135.82 (2C), 134.42 (3C), 131.84 (2C), 130.21 (2C), 128.12 (3C), 128.09, 123.70 (3C), 98.38, 74.64, 74.50, 71.93, 66.17, 65.24, 56.46, 48.27, 29.08 (2C), 27.03, 19.44.

***p*-Methylphenyl 2,3,4,6-tetra-*O*-acetyl-1-thio-β-D-galactopyranoside (S6)**[8]

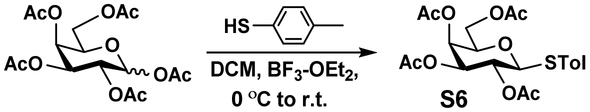

BF_3·OEt_2 (7.8 mL, 61.8 mmol) was added to a mixture of 1,2,3,4,6-penta-*O*-acetyl-D-galactopyranose (9.0 g, 23.1 mmol) and *p*-thiocresol (3.8 g, 31.0 mmol) in anhydrous CH_2Cl_2 (100 mL) at 0 °C. The mixture was stirred at 0 °C for 4 h before being warmed up to room temperature followed by continuous stirring for another 4 h. The reaction mixture was then diluted with CH_2Cl_2 (100 mL), washed sequentially with H_2O, a saturated aqueous solution of $NaHCO_3$, and brine. It was then dried over Na_2SO_4, filtered, and concentrated under reduced pressure. The residue was purified by silica gel chromatography using hexane:EtOAc = 3:1 (by volume) as an eluent to obtain *p*-methylphenyl 2,3,4,6-tetra-*O*-acetyl-1-thio-β-D-galactopyranoside **S6** (9.6 g, 92%) as a white solid. ^{1}H NMR (600 MHz, $CDCl_3$) δ 7.36 (d, J = 8.4 Hz, 2H), 7.07 (d, J = 8.4 Hz, 2H), 5.35 (d, J = 3.0 Hz, 1H), 5.16 (t, J = 10.2 Hz each, 1H), 4.99 (dd, J = 10.2, 3.6 Hz, 1H), 4.61 (d, J = 10.2 Hz, 1H), 4.13 (dd, , J = 11.4, 6.6 Hz, 1H), 4.06 (dd, J = 11.4, 6.6 Hz, 1H), 3.87 (t, J = 7.2 Hz, 1H), 2.29, 2.06, 2.04, 1.99, 1.92 (s, 3H for each). ^{13}C NMR (150 MHz, $CDCl_3$) δ 170.54, 170.38, 170.22, 169.60, 138.60, 133.30(2C), 129.83 (2C), 128.82, 87.03, 74.52, 72.20, 67.46, 67.43, 61.80, 21.35, 21.05, 20.86, 20.82, 20.77.

***p*-Methylphenyl 6-*tert-O*-butyldiphenylsilyl-2,3,4-tri-*O*-acetyl-1-thio-β-D-galactopyranoside (13)**

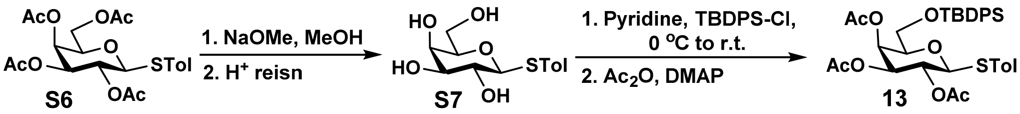

To a solution of **S6** (9.6 g, 21.12 mmol) in dry MeOH (100 mL), NaOMe (400 mg) was added. After being stirred for 2 h, the reaction mixture was neutralized with Dowex 50W (H^+), filtered, and concentrated to obtain *p*-methylphenyl 1-thio-β-D-galactopyranoside **S7** as a white solid (6.0 g, quantitative yield). The compound was used directly in the next step without further purification. *p*-Methylphenyl 1-thio-β-D-galactopyranoside **S7** (6.0 g, 19.97 mmol) dissolved in dry pyridine (40 mL) was cooled to 0 °C and TBDPSCl (6.2 mL, 23.97 mmol) was added drop-wisely. Reaction mixture was stirred at the same temperature for a period of 1 h and then at room temperature for overnight. When TLC (hexane:EtOAc = 2:1 by volume and detected with *p*-anisaldehyde sugar stain) showed complete consumption of the starting material, an excess amount of acetic anhydride (25 mL) and a catalytic amount of DMAP were added and the mixture was stirred for another 10 h. The reaction mixture was concentrated under reduced pressure and was diluted with CH_2Cl_2 (125 mL). The organic layer was successively washed sequentially with H_2O and a saturated aqueous solution of $NaHCO_3$, dried over Na_2SO_4, concentrated, and purified by silica gel chromatography using hexane:EtOAc = 5:1 (by volume) as an eluent to obtain compound **13** (12.9 g, 95% in two steps) as a white solid. ^{1}H NMR (800 MHz, $CDCl_3$) δ 7.61 (dd, *J* = 19.6, 6.8 Hz, 5H), 7.43 (ddd, *J* = 7.5, 5.2, 2.4 Hz, 3H), 7.40–7.35 (m, 4H), 7.06 (d, *J* = 7.9 Hz, 2H), 5.55 (d, *J* = 3.2 Hz, 1H), 5.18 (t, *J* = 10.0 Hz, 1H), 5.05 (dd, *J* = 9.9, 3.3 Hz, 1H), 4.64 (d, *J* = 10.0 Hz, 1H), 3.80–3.75 (m, 2H), 3.64–3.61 (m, 1H), 2.31 (s, 3H), 2.08 (d, *J* = 8.2 Hz, 3H), 1.98 (s, 3H), 1.97 (s, 3H), 1.02 (s, 9H). ^{13}C NMR (200 MHz, $CDCl_3$) δ 170.17, 170.10, 169.53, 138.18, 135.65, 135.62, 132.89 (2C), 132.79, 132.73 (3C), 129.91, 129.85, 129.69 (3C), 129.12, 127.81 (2C), 127.80, 87.25, 72.39, 67.62, 67.25, 61.43, 26.72 (3C), 21.17, 20.93, 20.70, 20.66, 19.05. HRMS (ESI) *m/z* calculated for $C_{35}H_{42}O_8SSiNa$ (M+Na) 673.2262, found 673.2257.

Phenyl 2,3,4-tri-*O*-acetyl-1-thio-β-L-fucopyranoside (S9)[9]

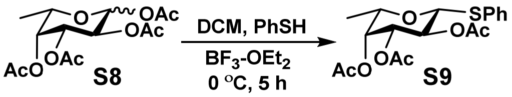

To an ice-cold solution of **S8** (6 g, 18 mmol) in dry dichloromethane (50 mL), thiophenol (2.0 mL, 23.5 mmol) and $BF_3{\cdot}OEt_2$ (5.1 mL, 36 mmol) were added and the reaction mixture was stirred at 5 °C for 5 h. The reaction mixture was diluted with CH_2Cl_2 (100 mL) and the organic layer was washed sequentially with H_2O and a saturated aqueous solution of $NaHCO_3$. The organic layer was dried over Na_2SO_4 and concentrated to dryness under reduced pressure. The product was purified by silica gel chromatography using hexane:EtOAc = 4:1 (by volume) as an eluent to obtain compound **S9** (6.2 g, 90%) as a white solid. ^{1}H NMR (600 MHz, $CDCl_3$) δ 7.51–7.33 (m, 2H), 7.32–7.13 (m, 3H), 5.21 (d, *J* = 3.0 Hz, 1H), 5.18 (t, *J* = 10.2 Hz, 1H), 5.01 (dd, *J* = 10.2, 3.6 Hz, 1H), 4.67 (d, *J* = 10.2 Hz, 1H), 3.79 (dd, *J* = 12.0, 6.6 Hz, 1H), 2.09 (s, 3H), 2.03 (s, 3H), 1.92 (s, 3H), 1.19 (d, *J* = 6.4 Hz, 3H). ^{13}C NMR (150 MHz, $CDCl_3$) δ 170.53, 170.04, 169.41, 132.84, 132.22 (2C), 128.82 (2C), 127.86, 86.35, 73.07, 72.36, 70.26, 67.28, 20.82, 20.62, 20.59, 16.43.

Phenyl 2,3,4-tri-*O*-benzyl-1-thio-β-L-fucopyranoside (14)[10]

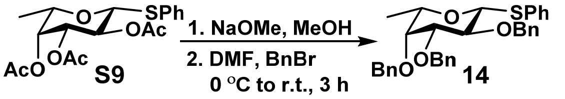

To a solution of the compound **S9** (6 g, 15.9 mmol) in MeOH, solid NaOMe (250 mg) was added. After being stirred at r.t. for 4 h, the reaction mixture was neutralized with Dowex 50W (H^+), filtered, and concentrated under reduced pressure to deacetylated intermediate as a white solid. This intermediate was used in the next step without further purification. To an ice-cold solution of the deacetylated compound in DMF (30 mL), powder NaH (60%) (3.5 g) and benzyl bromide (2 mL) were added and the reaction mixture was stirred at room temperature for 3 h. The reaction was quenched by adding cold MeOH, concentrated under reduced pressure and diluted with CH_2Cl_2 (75 mL). The organic layer was washed with H_2O, dried over Na_2SO_4, and concentrated under reduced pressure. The product was purified by silica gel chromatography using hexane:EtOAc =10:1 (by volume) as an eluent to obtain compound **14** (7 g, 86%) as a white solid. ^{1}H NMR (600 MHz, $CDCl_3$) δ 7.61–7.59 (m, 3H), 7.42–7.27 (m, 14H), 7.22 (m, 3H), 5.03 (d, *J* = 12.0 Hz, 1H), 4.81 (d, *J* = 10.2 Hz, 1H), 4.76 (s, 2.5H), 4.74 (s, .5H), 4.69 (d, *J* = 11.6 Hz, 1H), 4.62 (d, *J* = 9.6 Hz , 1H), 3.95 (t, *J* = 9.6 Hz each, 1H), 3.65 (d, *J* = 2.4 Hz, 1H), 3.61 (dd, *J* = 11.7, 2.8 Hz, 1H), 3.54 (q, *J* = 6.4 Hz, 1H), 1.28 (d, *J* = 6.4 Hz, 3H). ^{13}C NMR (150 MHz, $CDCl_3$) δ 138.69, 138.36, 138.32, 134.31, 131.49 (2C), 128.72 (2C), 128.42 (2C), 128.33 (2C), 128.30 (2C), 128.15 (2C), 127.92 (2C), 127.69, 127.67, 127.55 (2C), 127.45, 126.94, 87.51, 84.50, 77.09, 76.58, 75.56, 74.60, 74.57, 72.83, 17.30.

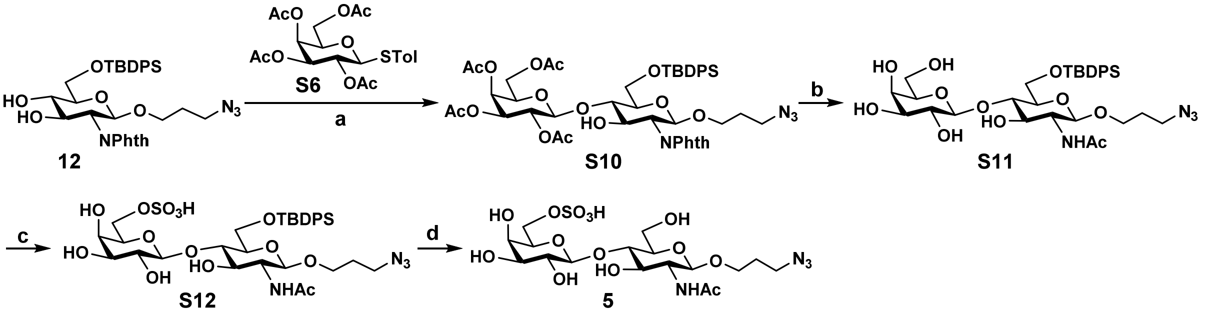

Scheme S1. Chemical synthesis of 6'-*O*-sulfo-LacNAcβN_3 (**5**). Reagents and conditions: a) *N*-iodosuccinimide (NIS), TMSOTf, MS 4 Å, CH_2Cl_2, -40 °C, 79%; b) (i) $H_2N(CH_2)_2NH_2$, *n*-BuOH, 90 °C, 8 h; (ii) THF, triethylamine, AcCl, 0 °C to r.t., 2 h, 69%; c) SO_3·Pyridine, DMF, 0 °C to r.t., 45%; d) THF, TBAF, r.t.

3-Azidopropyl 2,3,4,6-tetra-*O*-acetyl-β-D-galactopyranosyl-(1→4)-6-*O*-*t*-butyldiphenysilyl-2-deoxy-2-phthalimido-β-D-glucopyranoside (S10)

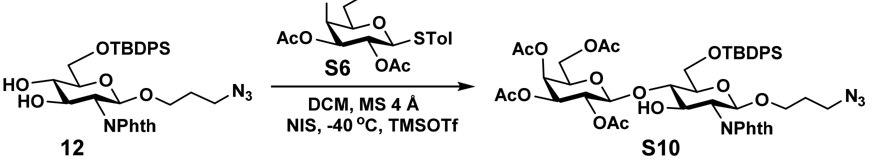

To a solution of 3-azidopropyl 6-*O*-*t*-butyldiphenylsilyl-2-deoxy-2-phthalimido-β-D-glucopyranoside **12** (500 mg, 0.79 mmol) and 2,3,4,6-tetra-*O*-acetyl-β-D-1-thiotolyl-β-D-galactopyranoside **S6** (435 mg, 1.15 mmol) in dry CH_2Cl_2 (15 mL), molecular sieve (MS) 4 Å (300 mg) was added and the reaction mixture was stirred at room temperature under N_2 for 1 h. *N*-Iodosuccinimide (NIS, 260 mg, 5.81 mmol) was added and the reaction mixture was cooled to -40

°C before adding TMSOTf (15 μL).[8] The mixture was stirred at the same temperature for 30 min. When TLC (hexane:EtOAc = 1.5:1 by volume and detected with *p*-anisaldehyde sugar stain) showed complete consumption of the acceptor **12**, the reaction mixture was diluted with CH_2Cl_2 (25 mL) and filtered through a Celite® bed. The organic layer was washed sequentially with 5% aqueous $Na_2S_2O_3$ and a saturated aqueous solution of $NaHCO_3$, dried over Na_2SO_4 and evaporated to dryness. The product was purified by silica gel chromatography using hexane:EtOAc = 3:1 (by volume) as an eluent to obtain disaccharide **S10** (595 mg, 79%) as a white solid. ^1H NMR (600 MHz, $CDCl_3$) δ 7.77–7.66 (m, 7H), 7.53–7.30 (m, 7H), 5.37–5.30 (m, 1H), 5.25–5.15 (m, 2H), 4.96 (dd, *J* =10.4, 3.5 Hz, 1H), 4.69 (d, *J* = 8.1 Hz, 1H), 4.48–4.40 (m, 1H), 4.19-3.81 (m, 8H), 3.60–3.53 (m, 1H), 3.52–3.44 (m, 1H), 3.17 (t, *J* = 6.7 Hz each, 2H), 2.12 (s, 3H), 1.97 (m, 6 H), 1.81–1.65 (m, 5H), 1.10 (s, 9H). ^{13}C NMR (150 MHz, $CDCl_3$) δ 170.57, 170.21, 170.03, 169.23, 136.09–123.71 (Ar-C), 101.39, 98.04, 81.29, 74.86, 71.39, 70.96, 69.59, 68.89, 66.98, 65.87, 62.06, 61.40, 56.33, 48.28, 29.05, 27.01 (3C), 20.71, 20.64, 20.52, 20.44, 19.54. HRMS (ESI) *m/z* calculated for $C_{47}H_{56}N_4O_{16}SiNa$ (M+Na) 983.3353, found 983.3356.

3-Azidopropyl β-D-galactopyranosyl-(1→4)-6-*O*-*t*-butyldiphenysilyl-2-deoxy-2-acetamido-β-D-glucopyranoside (S11)

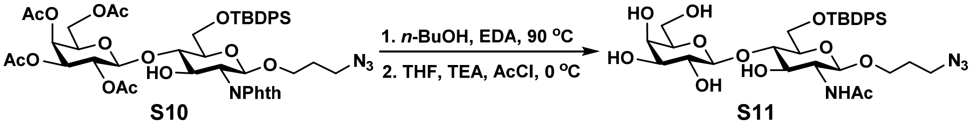

$H_2N(CH_2)_2NH_2$ (0.8 mL, 12 mmol) was added to a solution of compound **S10** (500 g, 0.52 mmol) in BuOH (10 mL), and the mixture was stirred at 90 °C for 8 h.[11] The reaction mixture was concentrated, co-evaporated with toluene (3 × 7 mL), and dried *in vacuo* for overnight. The solid residue was dissolved in methanol (20 mL), triethylamine (25 μL), and acetyl chloride (50 μL). The mixture was incubated in an ice-H_2O bath for 2 h until the completion of the reaction was achieved as indicated by TLC (DCM:MeOH = 15:1 by volume and detected with *p*-anisaldehyde sugar stain). The product was purified by silica gel chromatography using DCM:MeOH = 20:1 (by volume) as an eluent to obtain compound **S11** (252 g, 69%) as a white solid. ^1H NMR (600 MHz, CD_3OD) δ 7.84–7.60 (m, 4H), 7.47–7.12 (m, 6H), 4.54 (d, *J* = 7.7 Hz, 1H), 4.33 (d, *J* = 8.4 Hz, 1H), 4.19 (dd, *J* = 11.4 Hz, 2.7, 1H), 3.97–3.81 (m, 3H), 3.79–3.68 (m, 3H), 3.66–3.56 (m, 2H), 3.56–3.49 (m, 1H), 3.49–3.42 (m, 2H), 3.41–3.35 (m, 2H), 3.34–3.26 (m, 2H), 1.93 (s, 3H), 1.82–1.67 (m, 2H), 0.98 (s, 9H).^{13}C NMR (150 MHz, CD_3OD) δ 172.14, 135.68–127.28 (Ar-C), 103.29, 101.26, 77.74, 75.94, 75.27, 73.60, 72.67, 71.12, 68.97, 65.43, 61.92, 61.28, 55.34, 47.89, 28.76, 26.06 (3C), 21.72, 18.93. HRMS (ESI) *m/z* calculated for $C_{33}H_{48}N_4O_{11}SiNa$ (M+Na) 727.2981, found 727.2974.

3-Azidopropyl 6-*O*-sulfo-β-D-galactopyranosyl-(1→4)-6-*O*-*t*-butyldiphenysilyl-2-deoxy-2-acetamido-β-D-glucopyranoside (S12)

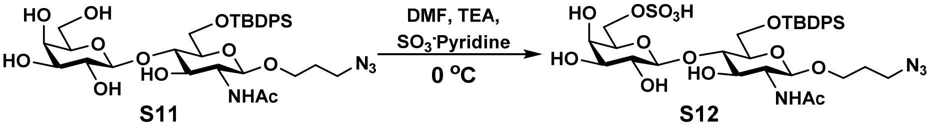

To an ice-cold solution of compound **S11** (193 mg, 0.274 mmol) in DMF (7.5 mL) and triethylamine (1.5 mL), sulphur trioxide-pyridine complex (262 mg, 1.66 mmol) was added. The resulting mixture was stirred at 0 °C for 1 h and then at room temperature for 5 h. When the reaction was completed as indicated by TLC (DCM:MeOH = 10:1 by volume and detected with *p*-

anisaldehyde sugar stain), the solvents were removed under reduced pressure, and the product was purified by silica gel chromatography using DCM:MeOH = 15:1 (by volume) to obtain compound **S12** as a white solid (97 mg, Yield 45%). ^{1}H NMR (600 MHz, CD_3OD) δ 7.90–7.67 (m, 4H), 7.52–7.16 (m, 6H), 4.51 (d, *J* = 7.7 Hz, 1H), 4.42 (d, *J* = 8.4 Hz, 1H), 4.26–4.10 (m, 3H), 3.99 (d, *J* = 11.3 Hz, 1H), 3.91–3.45 (m, 9H), 3.44 (dd, *J* = 9.7 Hz, 3.2, 1H), 3.35–3.33 (m, 2H), 1.98 (s, 3H), 1.88–1.72 (m, 2H), 1.02 (s, 9H). ^{13}C NMR (150 MHz, CD_3OD) δ 172.81, 135.57–127.27 (Ar-C), 103.60, 100.88, 78.96, 75.08, 73.54, 73.31, 73.23, 70.82, 68.52, 66.41, 65.70, 62.16, 55.24, 53.78, 28.71, 26.01 (3C), 21.67, 18.88. HRMS (ESI) *m/z* calculated for $C_{33}H_{47}N_4O_{14}SSi$ (M-H) 783.2584, found 783.2589.

3-Azidopropyl 6-*O*-sulfo-β-D-galactopyranosyl-(1→4)-2-deoxy-2-acetamido-β-D-glucopyranoside (5)

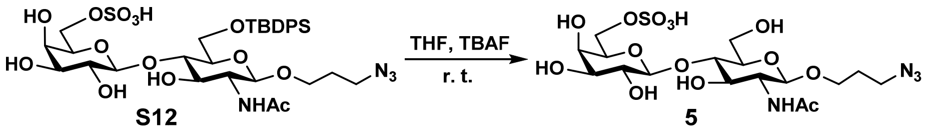

To a solution of compound **S12** (85 mg, 0.11 mmol) in THF (5 mL) at room temperature, TBAF (1 M) in THF (216 μL, 0.22 mmol) was added. When the reaction was completed as indicated by TLC (DCM:MeOH = 10:1 by volume and detected with *p*-anisaldehyde sugar stain), the solvents were removed under reduced pressure, and the product was purified by silica gel chromatography using DCM:MeOH = 8:1 (by volume) as an eluent to obtain compound **5** as a white solid (58 mg, quantitative). ^{1}H NMR (800 MHz, D_2O) δ 4.52–4.49 (m, 2H), 4.20–4.16 (m, 2H), 3.99–3.94 (m, 4H), 3.81 (dd, *J* = 12.3, 5.3 Hz, 1H), 3.75–3.64 (m, 5H), 3.61–3.59 (m, 1H), 3.54–3.52 (m, 1H), 3.40–3.32 (m, 2H), 2.03 (s, 3H), 1.86–1.79 (m, 2H). ^{13}C NMR (200 MHz, D_2O) δ 174.03, 102.28, 100.60, 78.67, 74.20, 72.29, 71.81, 71.62, 70.30, 67.72, 66.67, 66.60, 59.73, 54.65, 47.26, 27.60, 21.66. HRMS(ESI) *m/z* calculated for $C_{17}H_{29}N_4O_{14}S$ (M-H) 545.1406, found 545.1418.

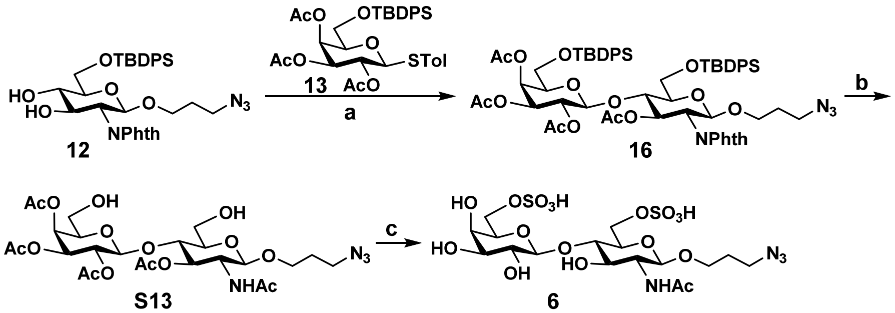

Scheme S2. Chemical synthesis of 6', 6-di-*O*-sulfo-LacNAcβN_3 (**6**). Reagents and conditions: a) *N*-iodosuccinimide (NIS), TMSOTf, MS 4 Å, CH_2Cl_2, -40 °C, 30 min, 78%; b) (i) $H_2N(CH_2)_2NH_2$, *n*-BuOH, 90 °C, 8 h; (ii) Pyridine, Ac_2O, r.t., 10 h; (iii) HF/Pyridine, 0 °C to r.t., overnight, 95% in three steps; c) (i) SO_3·Pyridine, Pyridine, 0 °C to r.t.; (ii) 0.1 M NaOMe, MeOH, r.t., 3 h, 85% in two steps.

3-Azidopropyl 2,3,4-tri-*O*-acetyl-6-*O*-*t*-butyldiphenylsilyl-β-D-galactopyranosyl-(1→4)-6-*O*-*t*-butyldiphenylsilyl-2-deoxy-2-phthalimido-β-D-glucopyranoside (16)

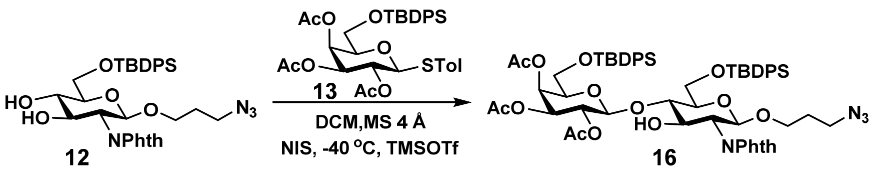

To a solution of 3-azidopropyl 6-*O*-*t*-butyldiphenylsilyl-2-deoxy-2-phthalimido-β-D-glucopyranoside **12** (1.6 g, 2.53 mmol) and 2,3,4-tri-*O*-acetyl-6-*O*-*t*-butyldiphenyl silyl-β-D-1-thiotolyl-β-D-galactopyranoside **13** (2.13 g, 3.29 mmol) in dry CH_2Cl_2 (70 mL), MS 4 Å (3.00 g) was added and the reaction mixture was stirred under N_2 at room temperature for 1 h. *N*-Iodosuccinimide (NIS, 886 mg, 3.94 mmol) was added and the reaction mixture was cooled to -40 °C before TMSOTf (70 mL) was added.[8] The reaction mixture was stirred at same temperature for 30 min. When TLC (hexane:EtOAc = 3:1 by volume and detected with *p*-anisaldehyde sugar stain) showed complete consumption of the acceptor **12**, the reaction mixture was diluted with CH_2Cl_2 (100 mL) and filtered through a Celite® bed. The organic layer was washed sequentially with 5% aqueous $Na_2S_2O_3$ and a saturated aqueous solution of $NaHCO_3$, dried over Na_2SO_4, and evaporated to dryness. The product was purified by silica gel chromatography using hexane:EtOAc = 3:1 (by volume) as an eluent to obtain disaccharide **16** (2.29 g, 78%) as a white solid. ^1H NMR (800 MHz, $CDCl_3$) δ 7.83 (d, *J* = 4.2 Hz, 1H), 7.79–7.72 (m, 5H), 7.69–7.66 (m, 2H), 7.53 (m, 4H), 7.45–7.31 (m, 11H), 5.41 (dd, *J* = 3.4, 0.7 Hz, 1H), 5.19–5.13 (m, 2H), 4.96 (dd, *J* = 10.5, 3.5 Hz, 1H), 4.69 (d, *J* = 8.0 Hz, 1H), 4.47 (dd, *J* = 10.8, 8.4 Hz, 1H), 4.15 (dd, *J* = 10.8, 8.5 Hz, 1H), 3.96 (dd, *J* = 11.1, 1.4 Hz, 1H), 3.87–3.82 (m, 3H), 3.79–3.75 (m, 1H), 3.71 (dd, *J* = 10.2, 6.7 Hz, 1H), 3.56 (ddd, *J* = 9.7, 3.6, 1.6 Hz, 1H), 3.50–3.44 (m, 2H), 3.15 (t, *J* = 6.7 Hz each, 2H), 1.95 (s, 3H), 1.94 (s, 3H), 1.77–1.66 (m, 2H), 1.65 (s, 3H), 1.09 (s, 9H), 0.90 (s, 9H). ^{13}C NMR (200 MHz, $CDCl_3$) δ 170.05, 169.95, 169.22, 168.41, 167.84, 136.05–123.16 (Ar-C), 101.22, 97.98, 80.92, 74.80, 73.83, 71.10, 69.59, 69.10, 66.83, 65.71, 61.98, 61.12, 56.07, 48.15, 28.91, 26.88 (3C), 26.58 (3C), 20.63, 20.56, 20.34, 19.42, 18.82. HRMS (ESI) *m/z* calculated for $C_{61}H_{72}N_4O_{15}Si_2Na$ (M+Na) 1179.4425, found 657.4434.

3-Azidopropyl 2,3,4-tri-*O*-acetyl-β-D-galactopyranosyl-(1→4)-3-*O*-acetyl-2-deoxy-2-phthalimido-β-D-glucopyranoside (S13)

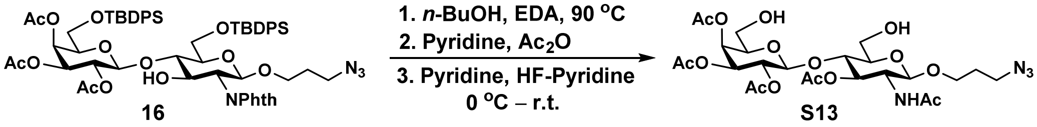

$H_2N(CH_2)_2NH_2$ (0.45 mL, 7 mmol) was added to a solution of compound **16** (550 mg, 0.45 mmol) in *n*-BuOH (10 mL), and the mixture was stirred at 90 °C for 8 h. The solvents were removed under reduced pressure and a solution of the crude product in Ac_2O·pyridine = 1:1 (by volume, 5 mL) was kept at r.t. for 10 h. The solvents were removed under reduced pressure and the remaining mixture was diluted with DCM. The organic layer was washed sequentially with HCl (1 N) and a saturated aqueous solution of $NaHCO_3$, and dried over Na_2SO_4. The solvents were removed under reduced pressure. The residue was dried *in vacuo* and used in the next step without further purification.

The dried crude product from above was dissolved in pyridine (6 mL) in a plastic flask followed by drop-wise addition of 65–70% HF pyridine solution (1.2 mL) at 0 °C. The solution was stirred for overnight until complete disappearance of the starting material as judged by TLC (tolene:EtOAc = 1:1 by volume and detected with *p*-anisaldehyde sugar stain) analysis. The reaction mixture was quenched by solid $NaHCO_3$, DCM (15 mL) and H_2O (15 mL) were added. The aqueous phase was extracted twice with dichloromethane. The combined organic phase was washed with a saturated

aqueous solution of $NaHCO_3$ and dried over Na_2SO_4. The solvents were removed under reduced pressure and the product was purified by silica gel chromatography using hexane:EtOAc = 1:1 (by volume) as an eluent to obtain **S13** (267 mg, 95%) as a white solid. ^1H NMR (600 MHz, $CDCl_3$) δ 5.72 (d, *J* = 9.3 Hz, 1H), 5.34 (d, *J* = 3.3 Hz, 1H), 5.21–4.96 (m, 3H), 4.67 (d, *J* = 7.9 Hz, 1H), 4.44 (d, *J* = 8.1 Hz, 2H), 4.10–3.84 (m, 2H), 3.79–3.63 (m, 3H), 3.57 (dd, *J* = 8.4, 4.0 Hz, 1H), 3.49 (dd, *J* = 11.7, 5.3 Hz, 2H), 3.39 (dd, *J* = 26.2, 10.2 Hz, 2H), 2.15 (s, 3H), 2.09 (s, 3H), 2.07 (s, 3H), 1.98 (s, 3H), 1.96 (s, 3H), 1.88–1.77 (m, 2H). ^{13}C NMR (150 MHz, $CDCl_3$) δ 171.48, 170.85, 170.32, 170.04, 169.54, 101.30, 100.81, 75.02, 74.42, 73.97, 73.48, 70.93, 69.61, 67.74, 65.93, 60.88, 60.61, 53.78, 47.89, 28.89, 23.27, 20.98, 20.73, 20.66, 20.58. HRMS (ESI) *m/z* calculated for $C_{25}H_{38}N_4O_{15}Na$ (M+Na) 657.2226, found 657.2229.

3-Azidopropyl 6-*O*-sulfo-β-D-galactopyranosyl-(1→4)-2-acetamido-2-deoxy-6-*O*-sulfo-β-D-glucopyranoside (6)

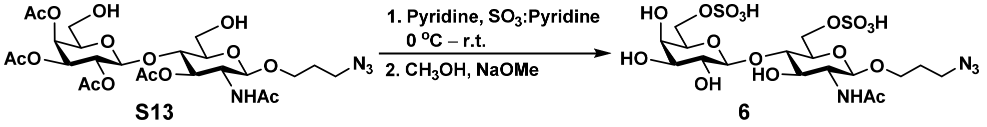

Compound **S13** (200 mg, 0.30 mmol) was dissolved in dry pyridine (5 mL) and cooled to 0 °C, followed by the addition of SO_3·Pyridine (1 g, 6.0 mmol). The resulting mixture was stirred at 0 °C for 30 min and then at room temperature for overnight until complete disappearance of the starting material as judged by TLC (DCM:MeOH = 7:1 by volume and detected with *p*-anisaldehyde sugar stain) analysis. The solvents were removed under reduced pressure. The residue was dried *in vacuo* and used in the next step without further purification. A solution of the crude product in CH_3ONa (0.1 M, 20 mL) was stirred at room temperature for 3 h and neutralized with Dowex 50W × 8 (H^+) resin. The reaction mixture was filtered and concentrated under reduced pressure. The product was purified by silica gel chromatography using DCM:MeOH= 6:1 (by volume) as an eluent to obtain **6** (167 mg, 85%) as a white solid. ^1H NMR (800 MHz, D_2O) δ 4.44 (d, *J* = 7.2 Hz, 1H), 4.35–4.30 (m, 1H), 4.20 (dd, *J* = 11.2, 5.6 Hz, 1H), 4.08 (m, 2H), 3.91–3.83 (m, 3H), 3.75–3.69 (m, 1H), 3.66–3.55 (m, 6H), 3.46–3.40 (m, 1H), 3.30–3.23 (m, 2H), 1.94 (s, 3H), 1.77–1.69 (m, 2H). ^{13}C NMR (200 MHz, D_2O) δ 174.03, 102.26, 100.64, 78.22, 72.22, 72.03, 71.77, 71.62, 70.32, 67.72, 66.73, 66.53, 66.06, 54.59, 47.27, 27.60, 21.66. HRMS(ESI) *m/z* calculated for $C_{17}H_{29}N_4O_{17}S_2$ (M-H) 625.0975, found 625.0941.

3-Azidopropyl 2,3,4-tri-*O*-acetyl-6-*O*-*t*-butyldiphenyl silyl-β-D-galactopyranosyl-(1→4)-6-*O*-benzyl-2-deoxy-2-phthalimido-β-D-glucopyranoside (15)

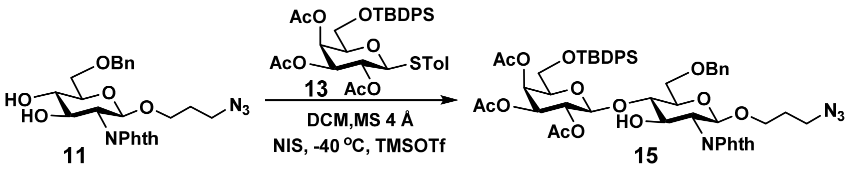

To a solution of 3-azidopropyl 6-*O*-benzyl-2-deoxy-2-phthalimido-β-D-glucopyranoside **11** (1.8 g, 3.73 mmol) and 2,3,4-tri-*O*-acetyl-6-*O*-*t*-butyldiphenylsilyl-β-D-1-thiotolyl-β-D-galactopyranoside **13** (3.15 g, 4.85 mmol) in dry CH_2Cl_2 (70 mL), MS 4 Å (3.00 g) was added and the reaction mixture was stirred under N_2 at room temperature for 1 h. *N*-Iodosuccinimide (NIS, 1.3 g, 5.81 mmol) was added and the reaction mixture was cooled to -40 °C before adding TMSOTf (70 μL).[8] The reaction mixture was stirred at the same temperature for 30 min. When TLC (hexane:EtOAc = 1:1 by volume and detected with *p*-anisaldehyde sugar stain) showed complete consumption of the

acceptor **11**, the reaction mixture was diluted with CH_2Cl_2 (100 mL) and filtered through a Celite® bed. The organic layer was washed sequentially with 5% aqueous $Na_2S_2O_3$ and a saturated aqueous solution of $NaHCO_3$, dried over Na_2SO_4, and evaporated to dryness. The product was purified by silica gel chromatography using hexane:EtOAc = 3:1 (by volume) as an eluent to obtain disaccharide **15** (2.71 g, 72%) as a white solid. ^{1}H NMR (800 MHz, $CDCl_3$) δ 8.22–7.17 (m, 19H), 5.37 (d, *J* = 3.2 Hz, 1H), 5.23–5.09 (m, 2H), 4.93 (dd, *J* = 10.5, 3.4 Hz, 1H), 4.75 (d, *J* = 12.1 Hz, 1H), 4.51 (ddd, *J* = 54.6, 11.5, 7.2 Hz, 3H), 4.48 (dd, *J* = 10.8, 8.3 Hz, 1H), 4.16 (dd, *J* = 10.8, 8.6 Hz, 1H), 3.94–3.87 (m, 1H), 3.74 (dt, *J* = 5.4, 5.0 Hz, 3H), 3.70–3.62 (m, 2H), 3.52 (ddd, *J* = 10.0, 7.6, 4.9 Hz, 1H), 3.46 (dd, *J* = 10.5, 6.1 Hz, 1H), 3.17 (ddd, *J* = 19.0, 12.2, 5.7 Hz, 2H), 1.99 (s, 3H), 1.96 (s, 3H), 1.95 (s, 3H), 1.71 (m, 2H), 0.84 (s, 9H). ^{13}C NMR (200 MHz, $CDCl_3$) δ 170.56, 170.46, 169.86, 138.71–128.26 (Ar-C), 101.97, 98.85, 82.21, 74.79, 74.33, 74.23, 71.52, 70.31, 69.53, 68.54, 67.36, 66.62, 61.94, 56.23, 48.54, 29.39, 26.98 (3C), 21.26, 21.10, 21.04, 19.21. HRMS (ESI) *m/z* calculated for $C_{52}H_{60}N_4O_{15}SiNa$ (M+Na) 1031.3716, found 1031.3711.

3-Azidopropyl 2,3,4-tri-*O*-acetyl-6-*O*-*t*-butyldiphenylsilyl-β-D-galactopyranosyl-(1→4)-[2,3,4-tri-*O*-benzyl-α-L-fucopyranosyl-(1→3)]-6-*O*-benzyl-2-deoxy-2-phthalimido-β-D-glucopyranoside (17)

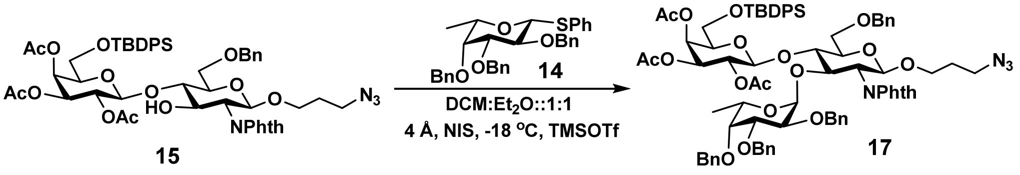

To a solution of disaccharide **15** (2.1 g, 2.08 mmol) and compound **14** (1.64 g, 3.12 mmol) in a mixture of anhydrous CH_2Cl_2 (15 mL) and Et_2O (15 mL), MS 4 Å (4.0 g) was added and the reaction mixture was stirred at room temperature under argon for 20 min. The reaction mixture was cooled to -18 °C and NIS (842 mg, 3.74 mmol) was added followed by the addition of TMSOTf (80 μL).[11] The reaction mixture was then stirred at the same temperature for 30 min. When TLC (toluene:EtOAc = 2:1 by volume and detected with *p*-anisaldehyde sugar stain) showed complete consumption of the acceptor **15**, the reaction mixture was diluted with CH_2Cl_2 (100 mL) and filtered through a Celite® bed. The organic layer was washed with sequentially with 5% aqueous $Na_2S_2O_3$ and a saturated aqueous solution of $NaHCO_3$, dried over Na_2SO_4, and evaporated to dryness. The product was purified by silica gel chromatography using hexane:EtOAc = 3:1 (by volume) as an eluent to obtain trisaccharide **17** (2.0 g, 68%) as a white solid. ^{1}H NMR (600 MHz, $CDCl_3$) δ 7.68 (s, 2H), 7.52 (d, *J* = 6.9 Hz, 1H), 7.48–7.32 (m, 15H), 7.31–7.07 (m, 13H), 7.00 (t, *J* = 7.5 Hz each, 1H), 6.87 (d, *J* = 7.4 Hz, 1H), 6.79 (d, *J* = 7.1 Hz, 1H), 5.50 (d, *J* = 2.9 Hz, 1H), 5.04–4.95 (m, 2H), 4.85 (d, *J* = 12.1 Hz, 1H), 4.82–4.69 (m, 2H), 4.64 (dt, *J* = 17.0, 9.3 Hz, 1H), 4.46 (t, *J* = 12.6 Hz each, 1H), 4.41–4.35 (m, 2H), 4.32 (d, *J* = 12.2 Hz, 3H), 4.25 (d, *J* = 10.9 Hz, 3H), 4.16–4.09 (m, 3H), 3.95–3.89 (m, 1H), 3.84 (ddd, *J* = 20.7, 12.9, 8.4 Hz, 2H), 3.68 (s, 1H), 3.49 (dd, *J* = 19.1, 9.6 Hz, 2H), 3.45–3.39 (m, 2H), 3.38 (s, 1H), 3.32 (dd, *J* = 9.2, 5.0 Hz, 1H), 3.11 (dt, *J* = 12.4, 6.3 Hz, 1H), 3.07–3.00 (m, 2H), 2.02 (s, 3H), 1.97 (s, 3H), 1.74 (s, 3H), 1.71–1.63 (m, 1H), 1.63–1.55 (m, 2H), 1.12 (d, *J* = 6.4 Hz, 3H), 0.99 (s, 9H). ^{13}C NMR (150 MHz, $CDCl_3$) δ 170.03, 169.59, 168.74, 138.73–123.55 (Ar-C), 99.66, 98.34, 97.24, 79.48, 77.50, 76.82, 75.37, 75.03, 74.22, 73.48, 72.77, 72.66, 72.50, 72.05, 71.28, 69.25, 67.60, 66.68, 66.20, 65.98, 59.84, 56.23, 47.92, 28.75, 26.63(3C), 20.75, 20.64, 20.60, 18.86, 16.51. HRMS (ESI) *m/z* calculated for $C_{79}H_{88}N_4O_{19}SiNa$ (M+Na) 1447.5704, found 1447.5721.

3-Azidopropyl 2,3,4-tri-*O*-acetyl-6-*O*-*t*-butyldiphenylsilyl-β-D-galactopyranosyl-(1→4)-[2,3,4-tri-*O*-benzyl-α-L-fucopyranosyl-(1→3)]-2-acetamido-2-deoxy-6-*O*-benzyl-β-D-glucopyranoside (19)

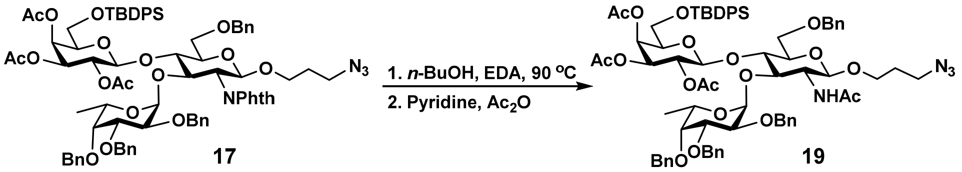

$H_2N(CH_2)_2NH_2$ (0.8 mL, 12 mmol) was added to a solution of compound **17** (1.6 g, 1.12 mmol) in BuOH (25 mL), and the mixture was stirred at 90 °C for 8 h.[11] The solvents were removed under reduced pressure and a solution of the crude product in Ac_2O·pyridine (10 mL, 1:1) was kept at r.t. for 10 h. The solvents were removed under reduced pressure and diluted with DCM (100 mL). The organic layer was sequentially washed with HCl (1 N) and a saturated aqueous solution of $NaHCO_3$, and dried over Na_2SO_4. The solvents were removed under reduced pressure. The residue was dried *in vacuo* and purified by silica gel chromatography using hexane:EtOAc = 2:1 (by volume) as an eluent to obtain trisaccharide **19** (1.25 g, 83%) as a yellow solid. ^{1}H NMR (800 MHz, $CDCl_3$) δ 7.66 (d, *J* = 7.0 Hz, 1H), 7.60 (d, *J* = 7.1 Hz, 1H), 7.55–7.33 (m, 26H), 7.23 (d, *J* = 6.9 Hz, 1H), 6.05 (d, *J* = 7.7 Hz, 1H), 5.68 (d, *J* = 3.1 Hz, 1H), 5.16–5.11 (m, 1H), 5.09 (d, *J* = 3.6 Hz, 1H), 5.06–4.96 (m, 1H), 4.71 (dd, *J* = 26.3, 11.8 Hz, 2H), 4.60 (t, *J* = 8.9 Hz each, 2H), 4.55 (d, *J* = 12.1 Hz, 1H), 4.50 (d, *J* = 11.3 Hz, 1H), 4.29 (d, *J* = 5.6 Hz, 1H), 4.18–4.08 (m, 2H), 3.99 (t, *J* = 6.4 Hz each, 1H), 3.90 (dddd, *J* = 27.4, 23.7, 9.8, 5.0 Hz, 5H), 3.79 (dd, *J* = 10.1, 2.3 Hz, 2H), 3.74–3.60 (m, 2H), 3.62–3.56 (m, 2H), 3.57–3.49 (m, 3H), 3.36 (m,2H), 2.13 (s, 3H), 2.09 (s, 3H), 2.00 (s, 3H), 1.87 (s, 3H), 1.81 (m, 2H), 1.10 (d, *J* = 10.1 Hz, 12H). ^{13}C NMR (200 MHz, $CDCl_3$) δ 170.43, 170.34, 170.07, 169.91, 138.97–127.55 (Ar-C), 100.02, 99.79, 97.68, 80.07, 77.54, 76.62, 74.76, 74.51, 73.95, 73.84, 73.68, 73.65, 73.15, 73.04, 71.15, 69.43, 68.98, 66.94, 66.74, 66.12, 60.37, 48.40 (2C), 29.14, 26.93 (3C), 23.41, 21.11, 20.98, 20.94, 19.23, 16.83. HRMS (ESI) *m/z* calculated for $C_{73}H_{88}N_4O_{18}SiNa$ (M+Na) 1359.5755, found 1359.5742.

3-Azidopropyl 2,3,4-tri-*O*-acetyl-β-D-galactopyranosyl-(1→4)-[2,3,4-tri-*O*-benzyl-α-L-fucopyranosyl-(1→3)]-2-acetamido-2-deoxy-6-*O*-benzyl-β-D-glucopyranoside (21)

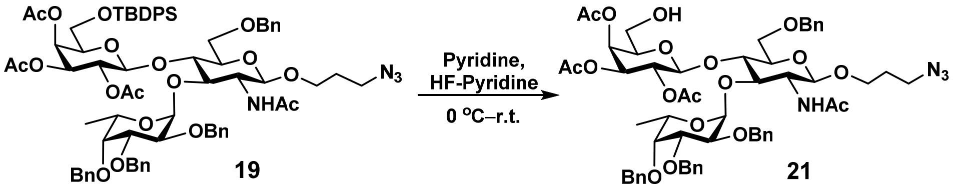

Compound **19** (1.2 g, 0.89 mmol) was dissolved in pyridine (10 mL) in a plastic flask followed by drop-wise addition of 65–70% HF·pyridine solution (1 mL) at 0 °C.[12] The solution was stirred for overnight until complete disappearance of the starting material as judged by TLC (toluene:acetone = 1:1 by volume and detected with *p*-anisaldehyde sugar stain) analysis. The reaction mixture was quenched by solid $NaHCO_3$, DCM (15 mL) and H_2O (15 mL) were added. The aqueous phase was extracted twice with DCM. The combined organic phase was washed with a saturated aqueous solution of $NaHCO_3$ and dried over Na_2SO_4. The solvents were removed under reduced pressure and the product was purified by silica gel chromatography using hexane:EtOAc = 1:1 (by volume) as an eluent to obtain compound **21** (907 mg, 92%) as a white solid. ^{1}H NMR (800 MHz, $CDCl_3$) δ 7.45–7.25 (m, 20H), 5.95 (d, *J* = 8.3 Hz, 1H), 5.46 (d, *J* = 3.5 Hz, 1H), 5.26 (dd, *J* = 10.2, 8.0 Hz, 1H), 4.93 (d, *J* = 11.5 Hz, 1H), 4.87 (d, *J* = 11.0 Hz, 1H), 4.82–4.75 (m, 2H), 4.73 (d, *J* = 11.0 Hz, 2H), 4.69 (d, *J* = 12.1 Hz, 1H), 4.65 (d, *J* = 11.6 Hz, 1H), 4.57 (d, *J* = 8.0 Hz, 1H), 4.49 (d, *J* = 12.1 Hz, 1H), 4.24–4.21 (m, 2H), 4.15 (s, 1H), 4.13 (d, *J* = 3.6 Hz, 1H), 4.11 (d, *J* = 3.6 Hz, 1H), 4.03 (dt, *J* = 24.3, 7.8 Hz, 2H), 3.92 (d, *J* = 2.7 Hz, 1H), 3.91 (d, *J* = 2.7 Hz, 1H), 3.89–3.84 (m, 2H), 3.78 (dd, *J* = 9.3, 5.7 Hz, 2H), 3.74 (d, *J* = 2.2 Hz, 1H), 3.44 (ddd, *J* = 9.6, 8.0, 4.8 Hz, 1H), 3.39–

3.33 (m, 1H), 3.30 (m, 2H), 2.13 (s, 3H), 2.04 (s, 3H), 1.83 (s, 3H), 1.78 (s, 3H), 1.76–1.70 (m, 2H), 1.18 (d, *J* = 6.4 Hz, 3H). ^{13}C NMR (200 MHz, $CDCl_3$) δ 170.52, 170.31, 170.12, 169.38, 138.64–127.11 (Ar-C), 101.42, 99.84, 96.36, 79.43, 77.35, 77.30, 75.13, 74.91 (2C), 73.96, 73.50, 73.26, 73.06, 71.92, 69.56, 69.45, 68.06, 67.43, 65.78, 63.09, 48.04 (2C), 28.88, 23.48, 20.94, 20.81, 16.67. HRMS (ESI) *m/z* calculated for $C_{57}H_{70}N_4O_{18}Na$ (M+Na) 1121.4577, found 1121.4563.

3-Azidopropyl 2,3,4-tri-*O*-acetyl-6-*O*-sulfo-β-D-galactopyranosyl-(1→4)-[2,3,4-tri-*O*-benzyl-α-L-fucopyranosyl-(1→3)]-2-acetamido-2-deoxy-6-*O*-benzyl-β-D-glucopyranoside (23)

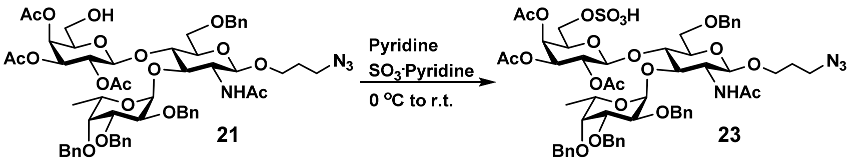

Compound **21** (800 mg, 0.73 mmol) was dissolved in dry pyridine (4 mL) and the mixture was cooled to 0 °C, followed by the addition of SO_3·Pyridine (1.16 g, 7.3 mmol).[13] The resulting mixture was stirred at 0 °C for 30 min and then at room temperature for overnight. The solvents were removed under reduced pressure, and the product was purified by silica gel chromatography using DCM:MeOH = 15:1 (by volume) as an eluent to obtain compound **23** (755 mg, 88%) as a white solid. ^{1}H NMR (800 MHz, $(CD_3)_2CO$) δ 7.55 (d, *J* = 7.5 Hz, 2H), 7.43 (d, *J* = 4.4 Hz, 6H), 7.35–7.20 (m, 12H), 5.40 (d, *J* = 3.5 Hz, 1H), 5.17 (t, *J* = 7.1 Hz, 2H), 5.06 (dd, *J* = 10.4, 8.2 Hz, 1H), 4.99–4.90 (m, 4H), 4.80 (ddd, *J* = 24.6, 14.4, 9.2 Hz, 2H), 4.68 (dd, *J* = 25.8, 11.5 Hz, 1H), 4.61 (d, *J* = 7.9 Hz, 1H), 4.54 (d, *J* = 12.0 Hz, 1H), 4.31 (dd, *J* = 10.2, 2.3 Hz, 1H), 4.23 (dd, *J* = 10.1, 7.1 Hz, 1H), 4.12 (t, *J* = 9.1 Hz each, 1H), 4.03–3.90 (m, 4H), 3.89–3.80 (m, 3H), 3.77 (dd, *J* = 10.8, 1.9 Hz, 1H), 3.56 (dt, *J* = 10.1, 6.2 Hz, 1H), 3.49 (d, *J* = 9.2 Hz, 1H), 3.43–3.36 (m, 2H), 2.90 (d, *J* = 25.1 Hz, 3H), 2.07 (s, 3H), 1.98 (s, 3H), 1.98 (s, 3H), 1.89 (s, 3H), 1.82–1.76 (m, 2H), 1.26 (d, *J* = 6.4 Hz, 3H). ^{13}C NMR (200 MHz, $(CD_3)_2CO$) δ 170.88, 169.73, 169.22, 168.66, 139.79–126.80 (Ar-C), 100.58, 100.05, 97.52, 78.98, 78.00, 74.91, 74.70, 74.55, 74.44, 74.37, 73.07, 72.81, 72.04, 71.41, 71.13, 69.01, 68.20, 67.95, 66.28, 65.55, 64.94, 55.45, 49.64, 48.02, 22.65, 20.25, 19.85, 19.67, 16.42. HRMS (ESI) *m/z* calculated for $C_{57}H_{69}N_4O_{21}S$ (M-H) 1177.4180, found 1177.4165.

3-Aminopropyl 6-*O*-sulfo-β-D-galactopyranosyl-(1→4)[α-L-fucopyranosyl-(1→3)]-2-acetamido-2-deoxy-β-D-glucopyranoside (9)

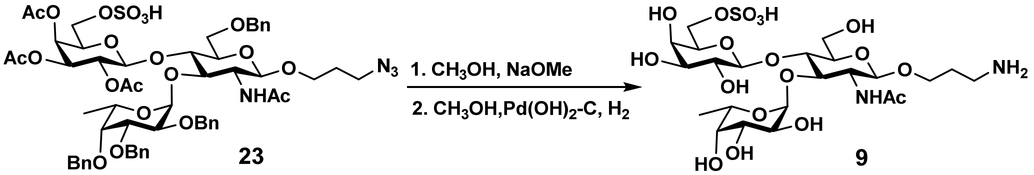

A solution of compound **23** (600 mg, 0.51 mmol) in CH_3ONa (0.1 M, 20 mL) was stirred at room temperature for 3 h and neutralized with Dowex 50W × 8 (H^+) resin. The reaction mixture was filtered and concentrated under reduced pressure. The residue was used in the next step without further purification. To a solution of the crude product in CH_3OH (30 mL), 20% $Pd(OH)_2$-C (200 mg)[14] was added and the reaction mixture was stirred at room temperature under a positive pressure of hydrogen for 48 h. The reaction mixture was filtered through a Celite® bed and evaporated to dryness. The product was purified by silica gel chromatography using EtOAc:MeOH:H_2O =2:2:1 (by volume) as an eluent to obtain **9** (135 mg, 40% in two steps^{1}H NMR (800 MHz, D_2O) δ 5.11 (d, *J* = 4.0 Hz, 1H), 4.52 (d, *J* = 8.8 Hz, 1H), 4.47 (d, *J* = 8.0 Hz, 1H), 4.18–4.11 (m, 2H), 4.04–

3.99 (m, 2H), 3.97–3.82 (m, 8H), 3.80 (d, *J* = 3.2 Hz, 1H), 3.73–3.64 (m, 3H), 3.63–3.60 (m 1H), 3.51 (dd, *J* = 9.6, 8.0 Hz, 1H), 3.07 (t, *J* = 7.2 Hz each, 2H), 2.03 (s, 3H), 1.97–1.88 (m, 2H), 1.17 (d, *J* = 6.4 Hz, 3H).^{13}C NMR (200 MHz, D_2O) δ 173.90, 101.17, 100.51, 98.05, 74.81, 74.59, 73.12, 71.80, 71.74, 71.49, 70.44, 68.64, 67.52, 67.39, 67.34, 66.69, 66.24, 59.33, 55.28, 37.22, 26.23, 21.71, 14.86. HRMS(ESI) *m/z* calculated for $C_{23}H_{41}N_2O_{18}S$ (M-H) 665.2081, found 665.2073.

3-Azidopropyl 2,3,4-tri-*O*-acetyl-6-*O*-*t*-butyldiphenyl silyl-β-D-galactopyranosyl-(1→4)-[2,3,4-tri-*O*-benzyl-α-L-fucopyranosyl-(1→3)]-6-*O*-*t*-butyldiphenylsilyl-2-deoxy-2-phthalimido-β-D-glucopyranoside (18)

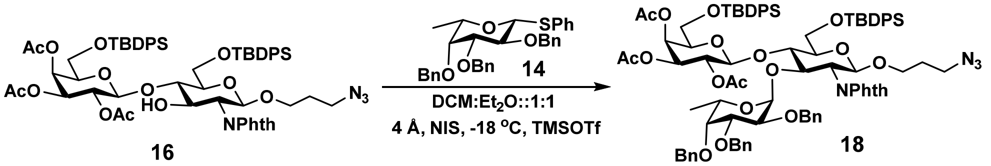

To a solution of disaccharide **16** (2.0 g, 1.73 mmol) and compound **14** (1.36 g, 2.59 mmol) in a mixture of anhydrous CH_2Cl_2 (15 mL) and Et_2O (15 mL), MS 4 Å (3.0 g) was added and the reaction mixture was stirred at room temperature under argon for 20 min. The reaction mixture was cooled to -18 °C and NIS (700 mg, 3.11 mmol) was added followed by the addition of TMSOTf (60 μL).[11] The reaction mixture was stirred at the same temperature for 45 min. When TLC (hexane:EtOAc = 3:1 by volume and detected with *p*-anisaldehyde sugar stain) showed complete consumption of the acceptor **16**, the reaction mixture was diluted with CH_2Cl_2 (100 mL) and filtered through a Celite® bed. The organic layer was washed sequentially with 5% aqueous $Na_2S_2O_3$ and a saturated aqueous solution of $NaHCO_3$, dried over Na_2SO_4, and evaporated to dryness. The product was purified by silica gel chromatography using hexane:EtOAc = 3:1 (by volume) as an eluent to obtain compound **18** (1.76 g, 65%) as a white solid. ^{1}H NMR (800 MHz, $CDCl_3$) δ 7.82–7.64 (m, 8H), 7.53–7.08 (m, 25 H), 7.01–6.96 (m, 2H), 6.84 (dt, *J* = 13.1, 6.7 Hz, 4H), 5.61 (d, *J* = 3.3 Hz, 1H), 5.05 (dt, *J* = 13.0, 8.9 Hz, 1H), 4.93 (dt, *J* = 7.0, 4.3 Hz, 1H), 4.74 (t, *J*= 7.5 Hz each, 1H), 4.72–4.67 (m, 2H), 4.47 (d, *J* = 11.7 Hz, 1H), 4.45–4.41 (m, 1H), 4.37 (d, *J* = 12.2 Hz, 1H), 4.28 (d, *J* = 10.9 Hz, 1H), 4.24 (dd, *J* = 13.0, 5.6 Hz, 1H), 4.14 (dd, *J* = 16.8, 11.6 Hz, 1H), 4.09 (dd, *J* = 15.0, 5.4 Hz, 1H), 4.00 (d, *J* = 11.6 Hz, 1H), 3.92–3.88 (m, 1H), 3.85–3.80 (m, 1H), 3.76 (dd, *J* = 10.3, 2.3 Hz, 1H), 3.74–3.70 (m, 1H), 3.67 (dt, *J* = 10.7, 5.4 Hz, 1H), 3.60–3.56 (m, 1H), 3.45–3.40 (m, 2H), 3.36 (dd, *J* = 13.5, 4.0 Hz, 1H), 3.13–3.05 (m, 2H), 1.99 (s, 3H), 1.86 (s, 3H), 1.79 (s, 3H), 1.75–1.69 (m, 1H), 1.67–1.60 (m, 1H), 1.17 (d, *J* = 6.4 Hz, 3H), 1.15 (s, 9H), 0.98 (s, 9H). ^{13}C NMR (200 MHz, $CDCl_3$) δ 169.77, 169.49, 168.65, 138.86–123.58 (Ar-C), 99.87, 98.07, 97.47, 79.62, 77.74, 75.86, 74.82, 74.28, 74.26, 72.96, 72.87, 72.53, 72.19, 71.43, 69.59, 66.83, 66.31, 65.40, 61.49, 59.98, 56.53, 48.11, 28.88, 26.89 (3C), 26.66 (3C), 26.62, 20.59, 20.49, 19.50, 18.92, 16.57. HRMS (ESI) *m/z* calculated for $C_{88}H_{101}N_4O_{19}Si_2$ (M+H) 1573.6592, found 1507.6581.

3-Azidopropyl 2,3,4-tri-*O*-acetyl-6-*O*-*t*-butyldiphenylsilyl-β-D-galactopyranosyl-(1→4)-[2,3,4-tri-*O*-benzyl-α-L-fucopyranosyl-(1→3)]-6-*O*-*t*-butyldiphenylsilyl-2-deoxy-2-acetamido-2-deoxy-β-D-glucopyranoside (20)

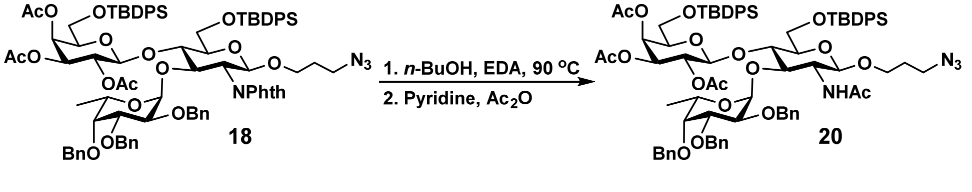

$H_2N(CH_2)_2NH_2$ (0.8 mL, 12 mmol) was added to a solution of compound **18** (1.6 g, 1.36 mmol) in *n*-BuOH (25 mL) and the mixture was stirred at 90 °C for 8 h. The solvent was removed under reduced pressure and a solution of the crude product in Ac_2O·pyridine = 1:1 (by volume, 10 mL) was kept at r.t. for 10 h. The solvents were removed under reduced pressure and the residue was diluted with DCM. The organic layer was washed sequentially with HCl (1 N) and a saturated aqueous solution of $NaHCO_3$, and dried over Na_2SO_4. The solvents were removed under reduced pressure. The product was purified by silica gel chromatography using hexane:EtOAc = 2:1 (by volume) as an eluent to obtain **20** (1.33 g, 88%) as a yellow solid. ^{1}H NMR (800 MHz, $CDCl_3$) δ 7.69 (d, *J* = 6.8 Hz, 2H), 7.55–7.52 (m, 3H), 7.50–7.23 (m, 28H), 7.12 (d, *J* = 6.7 Hz, 2H), 5.63 (d, *J* = 3.0 Hz, 1H), 5.05 (dd, *J* = 10.4, 8.0 Hz, 2H), 5.01–4.97 (m, 2H), 4.91 (d, *J* = 11.7 Hz, 1H), 4.74 (d, *J* = 11.8 Hz, 1H), 4.68 (d, *J* = 5.4 Hz, 1H), 4.62 (dd, *J* = 26.6, 11.8 Hz, 2H), 4.49 (d, *J* = 11.1 Hz, 1H), 4.35 (d, *J* = 11.1 Hz, 1H), 4.10–4.00 (m, 3H), 3.97 (dd, *J* = 11.2, 3.3 Hz, 2H), 3.79–3.73 (m, 2H), 3.73 (dd, *J* = 10.2, 2.5 Hz, 2H), 3.65 (dd, *J* = 9.6, 4.8 Hz, 2H), 3.60 (d, *J* = 9.4 Hz, 1H), 3.41 (dt, *J* = 10.1, 6.0 Hz, 2H), 3.34–3.29 (m, 1H), 3.25 (t, *J* = 6.8 Hz each, 2H), 2.01 (s, 3H), 1.91 (s, 3H), 1.89 (s, 3H), 1.76 (s, 3H), 1.74–1.69 (m, 2H), 1.07 (s, 9H), 1.05 (d, *J* = 6.5 Hz, 3H), 0.99 (s, 9H). ^{13}C NMR (200 MHz, $CDCl_3$) δ 170.29, 169.97, 169.77, 169.57, 138.70–127.20 (Ar-C), 99.44, 97.76, 80.13, 76.91, 76.32, 75.12, 74.49, 73.79, 73.61, 73.26, 72.89, 70.93, 69.29, 66.64, 66.50, 65.48, 62.14, 60.00, 48.22, 28.87, 26.81, 26.73 (3C), 26.65 (3C), 23.15, 20.73, 20.70, 19.32, 18.98, 16.66, 15.09. HRMS (ESI) *m/z* calculated for $C_{82}H_{100}N_4O_{18}Si_2Na$ (M+Na) 1507.6463, found 1507.6454.

3-Azidopropyl 2,3,4-tri-*O*-acetyl-β-D-galactopyranosyl-(1→4)-[2,3,4-tri-*O*-benzyl-α-L-fucopyranosyl-(1→3)]-2-acetamido-2-deoxy-β-D-glucopyranoside (22)

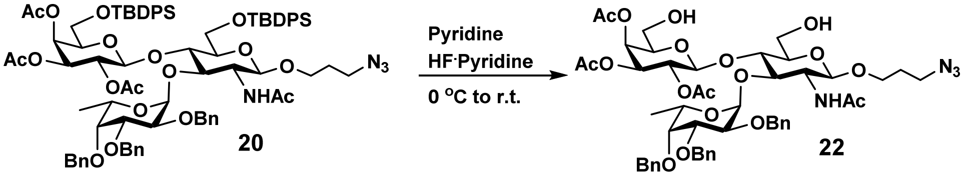

Compound **20** (1.25 g, 0.84 mmol) was dissolved in pyridine (10 mL) in a plastic flask followed by drop-wise addition of 65–70% HF·pyridine solution (3 mL) at 0 °C. The solution was stirred for overnight and the reaction mixture was quenched by solid $NaHCO_3$, DCM (15 mL) and H_2O (15 mL) were added. The aqueous phase was extracted twice with DCM. The combined organic phase was washed with a saturated aqueous solution of $NaHCO_3$ and dried over Na_2SO_4. The solvent was removed under reduced pressure and the product was purified by silica gel chromatography using hexane:EtOAc = 1:1 (by volume) as an eluent to obtain **22** (800 mg, 94%) as a white solid. ^{1}H NMR (800 MHz, $CDCl_3$) δ 7.41–7.23 (m, 15H), 5.32 (d, *J* = 3.3 Hz, 1H), 5.20 (d, *J* = 3.7 Hz, 1H), 5.12 (dd, *J* = 10.4, 3.4 Hz, 1H), 5.06 (dd, *J* = 10.3, 8.2 Hz, 1H), 4.99–4.95 (m, 1H), 4.92 (d, *J* = 11.8 Hz, 1H), 4.86 (dd, *J* = 12.7, 10.2 Hz, 3H), 4.76 (dd, *J* = 25.7, 11.9 Hz, 2H), 4.72–4.69 (m, 1H), 4.65–4.61 (m, 1H), 4.48 (t, *J* = 9.0 Hz each, 1H), 4.16 (dd, *J* = 10.1, 3.9 Hz, 1H), 3.99–3.96 (m, 3H), 3.92 (d, *J* = 12.5 Hz, 1H), 3.88 (d, *J*= 8.2 Hz, 1H), 3.83–3.76 (m, 3H), 3.68 (s, 1H), 3.58 (dd, *J* = 12.1, 8.7 Hz, 1H), 3.49–3.45 (m, 2H), 3.35–3.26 (m, 4H), 2.04 (s, 1H), 1.95 (s, 3H), 1.86 (s, 3H), 1.83 (s, 3H), 1.74–1.68 (m, 2H), 1.24 (d, *J* = 6.5 Hz, 3H). ^{13}C NMR (200 MHz, CDCl3) δ 170.73, 170.14, 170.04, 169.33, 138.67–127.16 (Ar-C), 99.90, 99.66, 96.92, 80.14, 77.16, 76.13, 76.00, 74.66, 74.33, 73.87, 73.56, 72.89, 72.80, 71.09, 69.50, 68.47, 66.93, 66.88, 61.64, 59.97, 47.88 (2C), 28.90, 23.56, 20.91, 20.74, 20.71, 16.91. HRMS (ESI) *m/z* calculated for $C_{50}H_{64}N_4O_{18}Na$ (M+Na) 1131.4113, found 1131.4108.

3-Azidopropyl 2,3,4-tri-*O*-acetyl-6-*O*-sulfo-β-D-galactopyranosyl-(1→4)-[2,3,4-tri-*O*-benzyl-α-L-fucopyranosyl-(1→3)]-2-acetamido-2-deoxy-6-*O*-sulfo-β-D-glucopyranoside (24)

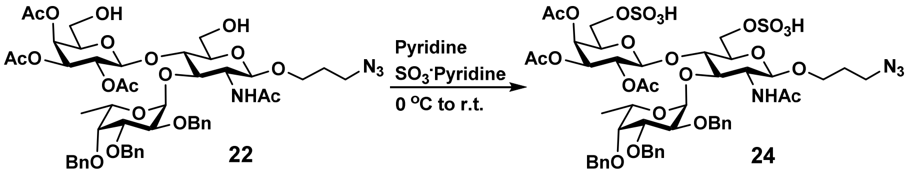

Compound **22** (750 mg, 0.74 mmol) was dissolved in dry pyridine (4 mL) and the solution cooled down to 0 °C, followed by the addition of SO_3·Pyridine (2.36 g, 14.8 mmol).[12] The resulting mixture was stirred at 0 °C for 30 min and then at room temperature for overnight. The solvents were removed under reduced pressure and the product was purified by silica gel chromatography using DCM:MeOH = 8:1 (by volume) as an eluent to obtain compound **24** (825 mg, 95%) as a white solid. ^{1}H NMR (800 MHz, DMSO-d$_6$) δ 7.44 (d, *J* = 7.4 Hz, 2H), 7.35–7.31 (m, 2H), 7.31–7.21 (m, 11H), 5.24 (d, *J* = 6.7 Hz, 1H), 5.22 (d, *J* = 3.5 Hz, 1H), 5.10 (d, *J* = 7.3 Hz, 1H), 4.97–4.93 (m, 2H), 4.87 (d, *J* = 11.8 Hz, 1H), 4.77 (q, *J* = 8.7 Hz, 2H), 4.69 (dd, *J* = 24.5, 12.0 Hz, 2H), 4.50 (d, *J* = 11.2 Hz, 1H), 4.42 (d, *J* = 12.4 Hz, 1H), 4.37 (d, *J* = 7.9 Hz, 1H), 4.06 (dd, *J* = 11.1, 3.6 Hz, 1H), 3.98 (d, *J* = 9.7 Hz, 1H), 3.89 (m, 5H), 3.81–3.73 (m, 2H), 3.71 (s, 1H), 3.63 (t, *J* = 9.3 Hz, 1H), 3.43 (m, 2H), 3.34 (m, 3H), 2.04 (s, 3H), 1.98 (s, 3H), 1.88 (s, 3H), 1.85 (s, 3H), 1.73–1.69 (m, 2H), 1.14 (d, *J* = 6.5 Hz, 3H). ^{13}C NMR (200 MHz, DMSO-d$_6$) δ 170.03, 169.91, 169.71, 169.63, 139.87–127.22 (Ar-C), 101.04, 99.24, 95.97, 78.40, 78.34, 75.65, 74.64, 74.62, 73.90, 73.56, 71.99, 71.17, 70.95, 70.76, 68.93, 67.51, 65.88, 65.70, 64.29, 63.00, 48.02, 46.27, 28.89, 23.65, 20.98, 20.97, 20.89, 16.81. HRMS (ESI) *m/z* calculated for $C_{50}H_{63}N_4O_{24}S_2$ (M-H) 1167.3279, found 1167.3269.

3-Aminopropyl 6-*O*-sulfo-β-D-galactopyranosyl-(1→4)[α-L-fucopyranosyl-(1→3)]-2-acetamido-2-deoxy-6-*O*-sulfo-β-D-glucopyranoside (10)

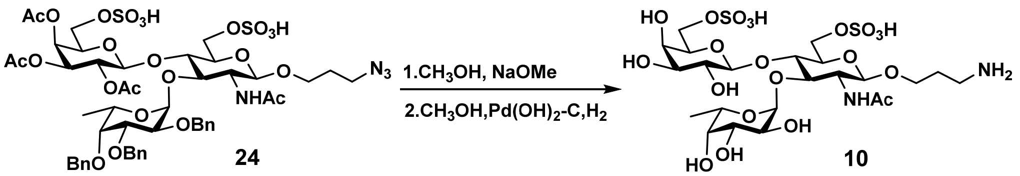

A solution of compound **24** (750 mg, 0.64 mmol) in 0.1 M CH_3ONa (20 mL) was stirred at room temperature for 3 h and neutralized with Dowex 50W × 8 (H^+) resin. The reaction mixture was filtered and concentrated under reduced pressure. The residue was used in the next step without further purification. To a solution of the crude product in CH_3OH (30 mL), 20% $Pd(OH)_2$/C (400 mg)[14] was added and the reaction mixture was stirred at room temperature under a positive pressure of hydrogen for 48 h. The reaction mixture was filtered through a Celite® bed and evaporated to dryness. The product was purified by silica gel chromatography using EtOAc:MeOH:H_2O = 2:2:1 (by volume) as an eluent to obtain **10** (184 mg, 42% in two steps). ^{1}H NMR (800 MHz, D_2O) δ 5.10 (d, *J* = 3.2 Hz, 1H), 4.56 (d, *J* = 8.0 Hz, 1H), 4.52 (d, *J* = 8.8 Hz, 1H), 4.42–4.34 (m, 2H), 4.17–4.11 (m, 2H), 4.02–3.92 (m, 4H), 3.91–3.79 (m, 6H), 3.76–3.73 (m, 1H), 3.69–3.64 (m, 2H), 3.50 (dd, *J* = 9.6, 8.0 Hz, 1H), 3.09 (t, *J* = 6.4 Hz, 2H), 2.02 (s, 3H), 1.97–1.91 (m, 2H), 1.17 (d, *J* = 6.4 Hz, 3H).^{13}C NMR (200 MHz, D_2O) δ 173.89, 101.18, 100.49, 98.10, 74.52, 72.83, 72.45, 71.74, 71.70, 71.50, 70.36, 68.62, 67.80, 67.47, 67.34, 66.56, 66.24, 65.59, 55.06, 37.38, 26.23, 21.69, 14.86. HRMS (ESI) *m/z* calculated for $C_{23}H_{41}N_2O_{21}S_2$ (M-H) 745.1649, found 745.1638.

Sequential one-pot multienzyme (OPME) synthesis of 6-*O*-sulfo-LacNAcβProN$_3$ (4) and 6-*O*-sulfo-Le^{x}βProN$_3$ (8)

3-Azidopropyl β-D-galactopyranosyl-(1→4)-2-acetamido-2-deoxy-6-*O*-sulfo-β-D-glucopyranoside (4)[3]

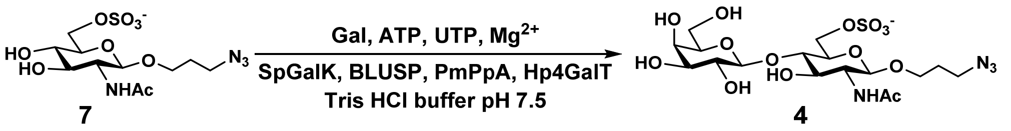

6-*O*-Sulfo-GlcNAcβProN$_3$ (**7**)[3] (200 mg, 0.52 mmol), Gal (112 mg, 0.62 mmol), UTP (549 mg, 1.04 mmol), and ATP (573 mg, 1.04 mmol) were dissolved in water in a 50 mL centrifuge tube containing Tris-HCl buffer (100 mM, pH 7.5) and $MgCl_2$ (20 mM). After adding SpGalK (2 mg), BLUSP (2 mg), PmPpA (0.3 mg), and Hp4GalT (2 mg), water was added to bring the final volume of the reaction mixture to 20 mL. The reaction was incubated at 37 °C for 48 h in a water bath. Product formation was monitored by TLC (EtOAc:MeOH:H_2O = 5:2:1 by volume with *p*-anisaldehyde sugar stain). The reaction was stopped by adding the same volume of ice-cold ethanol and incubating at 4 °C for 30 min. The mixture was then centrifuged. The supernatant was concentrated and passed through a BioGel P-2 gel filtration column followed by silica gel column purification using EtOAc:MeOH:H_2O = 5:2:0.5 (by volume) as an eluent to obtain the desired product (**4**) in 89% yield as a white solid. ^{1}H NMR (800 MHz, D_2O) δ 4.56–4.50 (m, 2H), 4.40 (d, *J* = 10.4 Hz, 1H), 4.35–4.29 (m, 1H), 3.98–3.94 (m, 1H), 3.91 (d, *J* = 3.2 Hz, 1H), 3.83–3.63 (m, 9H), 3.52 (m, 1H), 3.40–3.33 (m, 2H), 2.04 (s, 3H), 1.87–1.79 (m, 2H).^{13}C NMR (200 MHz, D_2O) δ 174.01, 102.02, 100.68, 76.89, 74.85, 72.04, 71.97, 71.78, 70.49, 68.11, 66.76, 65.78, 60.55, 54.60, 47.28, 27.61, 21.67. HRMS(ESI) *m/z* calculated for $C_{17}H_{29}N_4O_{14}S$ (M-H) 545.1406, found 545.1409.

3-Azidopropyl β-D-galactopyranosyl-(1→4)[α-L-fucopyranosyl-(1→3)]-2-acetamido-2-deoxy-6-*O*-sulfo-β-D-glucopyranoside (8)

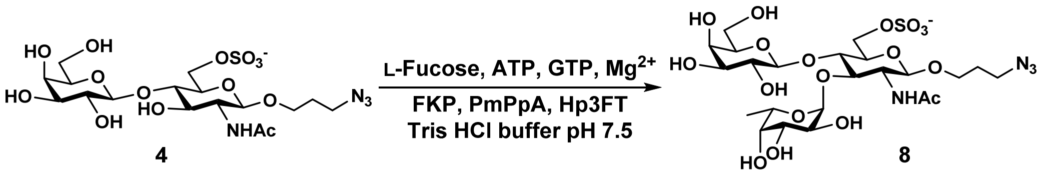

In a 15 mL centrifugal tube, 0.11 mmol (62 mg) of 6-*O*-sulfo-LacNAcβProN$_3$ (**4**), L-Fucose (1.5 eq., 0.165 mmol, 28 mg), ATP (1.5 eq., 0.165 mmol, 94 mg), GTP (1.5 eq., 0.165 mmol, 97 mg) were dissolved in a few mL of deionized water. The pH of the mixture was adjusted to neutral using NaOH (4 M). Mg^{2+} (20 mM) and Tris-HCl buffer (pH 7.5, 100 mM) were added. After mixing, Hp3FT (0.85 mg), PmPpA (1 mg), and FKP (1 mg) were added and the total volume of the reaction mixture was brought to 9 mL using deionized water.[15] The reaction mixture was incubated at 37 °C in a water bath for 2 h and then at room temperature for 36 h. The reaction progress was monitored using mass spectrometry and TLC. The reaction was stopped by adding the same volume of ice-cold ethanol and incubating at 4 °C for 30 min. The mixture was then centrifuged. The supernatant was concentrated and passed through a BioGel P-2 gel filtration column followed by silica gel column using EtOAc:MeOH:H_2O = 7.5:2:1 (by volume) as an eluent and the polarity of the solvent was increased to 7:2:1. The desired product 6-*O*-sulfo-Le^{x}βProN$_3$ (**8**) was obtained in 70% yield. ^{1}H NMR (800 MHz, D_2O) δ 5.10 (d, *J* = 4.0 Hz, 1H), 4.56 (d, *J* = 8.0Hz, 1H), 4.53 (d, *J* = 8.0 Hz, 1H), 4.39–4.32 (m, 2H), 3.99 (t, *J* = 9.2 Hz each, 1H), 3.97–3.92 (m, 1H), 3.92–3.85 (m,

4H), 3.81–3.76 (m, 2H), 3.74–3.63 (m, 6H), 3.61–3.58 (m, 1H), 3.46 (t, *J* = 8.0 Hz each, 1H), 3.40–3.32 (m, 2H), 2.03 (s, 3H), 1.87–1.79 (m, 2H), 1.16 (d, *J* = 6.4 Hz, 3H). ^{13}C NMR (200 MHz, D_2O) δ 173.76, 101.02, 100.45, 98.11, 74.47, 74.29, 72.46, 72.26, 71.92, 71.41, 70.48, 68.69, 67.93, 67.26, 66.77, 66.23, 65.45, 60.96, 55.19, 47.27, 27.61, 21.72, 14.81. HRMS (ESI) *m/z* calculated for $C_{23}H_{39}N_4O_{18}S$ (M-H) 691.1980, found 691.2010.

One-pot two-enzyme synthesis of *O*-sulfated sLe^x tetrasaccharides 1a–3a and 1b–3b

General procedures for one-pot two-enzyme preparative synthesis of *O*-sulfated sialyl Lewis x glycans

O-Sulfated Lewis x (6'-*O*-sulfo-Le^{x}βProNH$_2$, 6,6'-di-*O*-sulfo-Le^{x}βProNH$_2$, or 6-*O*-sulfo-Le^{x}βProN$_3$) oligosaccharide (30–80 mg), a sialic acid (Neu5Ac or Neu5Gc, 1.5 equiv.), and CTP (1.5 equiv.) were dissolved in Tris-HCl buffer (6 mL, pH 8.5) containing $MgCl_2$ (20 mM), NmCSS (2–3 mg) and PmST1 M144D (5–8 mg). The reaction mixture was incubated at 37 °C for 30 h in an incubator shaker.[4, 16] The reaction was monitored by TLC (*n*-PrOH:H_2O:NH_4OH = 4:2:1 by volume and detected by *p*-anisaldehyde sugar stain) and mass spectrometry. Additional CTP (0.5–1 equiv.) was added during the reaction process. When an optimal yield was achieved, 6 mL of EtOH was added to the reaction mixture and the resulting mixture was incubated at 4 °C for 30 min. The precipitates were removed by centrifugation. The supernatant was concentrated and purified by a Bio-Gel P-2 gel column (water was used as an eluent). Then the product was further purified by preparative HPLC using C18 column (CH_3CN in H_2O gradient was used as running solvents).

3-Azidopropyl 5-acetamido-3,5-dideoxy-D-glycero-α-D-galacto-nonulopyranosylonic acid-(2→3)-β-D-galactopyranosyl-(1→4)[α-L-fucopyranosyl-(1→3)]-2-acetamido-2-deoxy-6-*O*-sulfo-β-D-glucopyranoside (1a)

6-*O*-Sulfo-Neu5Acα2–3Le^{x}βProN$_3$ tetrasaccharide (80 mg, 85%) was obtained as a white foam. ^{1}H NMR (800 MHz, D_2O) δ 5.08 (d, *J* = 4.0 Hz,1H), 4.60 (d, *J* = 8.0 Hz, 1H), 4.54 (d, *J* = 7.2 Hz, 1H), 4.38–4.33 (m, 2H), 4.07 (dd, *J* = 9.6 Hz and 4.0 Hz, 1H), 4.00 (t, *J* = 9.6 Hz each, 1H), 3.96–3.64 (m, 18H), 3.57 (d, *J* = 8.8 Hz, 2H), 3.49 (dd, *J* = 9.6 Hz and 8.0 Hz, 1H), 3.38–3.32 (m, 2H), 2.73 (dd, *J* = 12.8 Hz and 4.8 Hz, 1H), 2.02 (s, 3H), 2.01 (s, 3H), 1.84–1.80 (m, 2H), 1.78 (t, *J* = 12.6 Hz each, 1H), 1.15 (d, *J* = 6.4 Hz, 3H). ^{13}C NMR (200 MHz, D_2O) δ 174.79, 174.11, 171.88, 100.92, 100.84, 98.45, 98.22, 75.41, 74.64, 74.59, 73.00, 72.67, 72.40, 71.77, 70.48, 69.19, 69.08, 67.93, 67.64, 67.51, 67.14, 67.03, 66.55, 65.68, 62.77, 61.17, 55.58, 51.54, 47.64, 39.19, 27.98, 22.08, 21.94, 15.15. HRMS (ESI) *m/z* calculated for $C_{34}H_{56}N_5O_{26}S$ (M-H) 982.2934, found 982.2937.

3-Azidopropyl 5-glycolyl-3,5-dideoxy-D-glycero-α-D-galacto-nonulopyranosylonic acid-(2→3)-β-D-galactopyranosyl-(1→4)[α-L-fucopyranosyl-(1→3)]-2-acetamido-2-deoxy-6-*O*-sulfo-β-D-glucopyranoside (1b)

6-*O*-Sulfo-Neu5Gcα2–3Le^{x}βProN$_3$ tetrasaccharide (22 mg, 47%) was obtained as a white foam. ^{1}H NMR (800 MHz, D_2O) δ 5.09 (d, *J* = 4.0 Hz, 1H), 4.60 (d, *J* = 8.0 Hz, 1H), 4.54 (d, *J* = 8.4 Hz, 1H), 4.39-4.32 (m, 2H), 4.10 (s, 2H), 4.08 (dd, *J* = 10.4 Hz and 4.0 Hz, 1H), 4.00 (t, *J* = 9.6 Hz each, 1H), 3.96–3.83 (m, 8H), 3.78–3.74 (m, 4H), 3.68–3.62 (m, 6H), 3.59–3.56 (m, 2H), 3.49 (dd, *J* = 9.6 Hz and 8.0 Hz, 1H), 3.38–3.32 (m, 2H), 2.75 (dd, *J* = 12.8 Hz and 4.8 Hz, 1H), 2.02 (s, 3H), 1.83–1.80 (m, 2H), 1.78 (t, *J* = 12.6 Hz each, 1H), 1.15 (d, *J* = 6.4 Hz, 3H). ^{13}C NMR (200 MHz, D_2O) δ 175.88, 174.40, 174.26, 101.33, 101.13, 99.70, 98.74, 75.51, 74.98, 74.86, 73.02, 72.76, 72.06, 71.53, 69.54, 69.33, 68.31, 68.14, 67.89, 67.42, 66.86, 66.02, 62.62, 61.64, 61.13, 55.83, 51.58, 47.92, 39.95, 28.25, 22.36, 15.44. HRMS (ESI) *m/z* calculated for $C_{34}H_{56}N_5O_{27}S$ (M-H) 998.2883, found 998.2916.

3-Aminopropyl 5-acetamido-3,5-dideoxy-D-glycero-α-D-galacto-nonulopyranosylonic acid-(2→3)-6-*O*-sulfo-β-D-galactopyranosyl-(1→4)[α-L-fucopyranosyl-(1→3)]-2-acetamido-2-deoxy-β-D-glucopyranoside (2a)

6'-*O*-Sulfo-Neu5Acα2–3Le^{x}βProNH$_2$ tetrasaccharide (75 mg, 82%) was obtained as a white foam. ^{1}H NMR (800 MHz, D_2O) δ 5.09 (d, *J* = 4.0 Hz, 1H), 4.53 (d, *J* = 8.0 Hz, 1H), 4.50 (d, *J* = 8.8 Hz, 1H), 4.14–4.06 (m, 3H), 4.03–3.98 (m, 2H), 3.98–3.90 (m, 3H), 3.90–3.80 (m, 7H), 3.77 (d, *J* = 2.4 Hz, 1H), 3.72–3.68 (m, 4H), 3.65–3.56 (m, 4H), 3.53 (t, 1H), 3.07 (t, *J* = 6.4 Hz each, 2H), 2.75 (dd, *J* = 12.8, 4.8 Hz, 1H), 2.02 (s, 6H), 1.97–1.88 (m, 2H), 1.81 (t, *J* = 12.6 Hz each, 1H), 1.15 (d, *J* = 6.4 Hz, 3H). ^{13}C NMR (200MHz, D_2O) δ 174.50, 173.88, 172.59, 100.90, 100.52, 98.75, 98.01, 74.93, 74.72, 74.51, 73.12, 72.51, 71.74, 71.48, 71.04, 68.66, 68.61, 67.63, 67.53, 67.35, 66.79, 66.43, 66.19, 62.23, 59.20, 58.79, 55.26, 51.14, 39.10, 37.24, 26.12, 21.68, 21.53, 14.83.HRMS (ESI) *m/z* calculated for$C_{34}H_{58}N_3O_{26}S$ (M-H) 956.3035, found 956.3037.

3-Aminopropyl 5-glycolyl-3,5-dideoxy-D-glycero-α-D-galacto-nonulopyranosylonic acid-(2→3)-6-*O*-sulfo-β-D-galactopyranosyl-(1→4)[α-L-fucopyranosyl-(1→3)]-2-acetamido-2-deoxy-β-D-glucopyranoside (2b).

6'-*O*-Sulfo-Neu5Gcα2–3Le^{x}βProNH$_2$ tetrasaccharide (45 mg, 60%) was obtained as a white foam. ^{1}H NMR (800 MHz, D_2O) δ 5.09 (d, *J* = 3.2 Hz, 1H), 4.53 (d, *J* = 8.0 Hz, 1H), 4.50 (d, *J* = 8.8 Hz, 1H), 4.14–4.07 (m, 5H), 4.03–3.90 (m, 6H), 3.90–3.78 (m, 8H), 3.77 (d, *J* = 2.6 Hz, 1H), 3.73–3.67 (m, 2H), 3.65–3.58 (m, 3H), 3.57 (d, *J* = 9.6 Hz, 1H), 3.54 (t, *J* = 8.8 Hz each, 1H), 3.07 (t, *J* = 6.4 Hz each, 2H), 2.77 (dd, *J* = 12.8, 4.8 Hz, 1H), 2.02 (s, 3H), 1.97–1.89 (m, 2H), 1.83 (t, *J* = 12.6 Hz each, 1H), 1.15 (d, *J* = 6.4 Hz, 3H).^{13}C NMR (200 MHz, D_2O) δ 175.59, 174.11, 173.97, 101.05, 100.85, 99.43, 98.45, 75.24, 74.71, 74.58, 72.76, 72.50, 72.46, 71.77, 71.26, 69.27, 69.04, 68.05, 67.85, 67.63, 67.14, 67.09, 66.59, 65.74, 62.35, 61.37, 60.85, 55.56, 51.31,47.65, 39.67, 34.03, 27.99, 22.10, 15.17. HRMS (ESI) *m/z* calculated for $C_{34}H_{58}N_3O_{27}S$ (M-H) 972.2984, found 972.2974.

3-Aminopropyl 5-acetamido-3,5-dideoxy-D-glycero-α-D-galacto-nonulopyranosylonic acid-(2→3)-6-*O*-sulfo-β-D-galactopyranosyl-(1→4)[α-L-fucopyranosyl-(1→3)]-2-acetamido-2-deoxy-6-*O*-sulfo-β-D-glucopyranoside (3a)

6,6'-Di-*O*-sulfo-Neu5Acα2–3Le^{x}βProNH$_2$ tetrasaccharide (42 mg, 64%) was obtained as a white foam. ^{1}H NMR (800 MHz, D_2O) δ 5.09 (d, *J* = 4.0 Hz, 1H), 4.58 (d, *J* = 8.0 Hz, 1H), 4.55 (d, *J* = 8.0 Hz, 1H), 4.41–4.35 (m, 2H), 4.15–4.05 (m, 3H), 4.02 (t, *J* = 9.6 Hz each, 1H), 4.00–3.92 (m, 3H), 3.92–3.80 (m, 8H), 3.76 (m, 2H), 3.68–3.60 (m, 4H), 3.58 (d, *J* = 9.6 Hz, 1H), 3.54–3.49 (m, 1H), 3.10 (t, *J* = 6.4 Hz each, 2H), 2.73 (dd, *J* = 12.8, 4.8 Hz, 1H), 2.01 (s, 6H), 1.98–1.89 (m, 2H), 1.78 (t, *J* = 12.6 Hz each, 1H), 1.15 (d, *J* = 6.4 Hz, 3H). ^{13}C NMR (200 MHz, D_2O) δ 174.44, 173.85, 173.44, 100.76, 100.48, 99.22, 98.02, 74.69, 74.50, 72.78, 72.39, 71.70, 71.50, 70.90, 68.78, 68.58, 67.94, 67.82, 67.57, 67.35, 66.93, 66.54, 66.22, 65.58, 62.21, 62.06, 55.01, 51.18, 39.02, 37.42, 26.22, 21.65, 21.53, 14.85. HRMS (ESI) *m/z* calculated for $C_{34}H_{58}N_3O_{29}S_2$(M-H) 1036.2603, found 1036.2609.

3-Aminopropyl 5-glycolyl-3,5-dideoxy-D-glycero-α-D-galacto-nonulopyranosylonic acid-(2→3)-6-*O*-sulfo-β-D-galactopyranosyl-(1→4)[α-L-fucopyranosyl-(1→3)]-2-acetamido-2-deoxy-6-*O*-sulfo-β-D-glucopyranoside (3b)

6,6'-Di-*O*-sulfo-Neu5Gcα2–3Le^{x}βProNH$_2$ tetrasaccharide (40 mg, 38%) was obtained as a white

foam. ^{1}H NMR (800 MHz, D_2O) δ 5.09 (d, J = 4.0 Hz, 1H), 4.59 (d, J = 8.0 Hz, 1H), 4.56 (d, J = 8.0 Hz, 1H), 4.40–4.37 (m, 2H), 4.14–4.07 (m, 5H), 4.03 (t, J = 9.6 Hz each, 1H), 3.98–3.82 (m, 11H), 3.78–3.74 (m, 4H), 3.64–3.62 (m, 2H), 3.58 (d, J = 9.3 Hz, 1H), 3.52 (t, J = 9.6 Hz each, 1H), 3.10 (t, J = 9.6 Hz each, 2H), 2.75 (dd, J = 12.8, 4.8 Hz, 1H), 2.01 (s, 3H), 1.98–1.90 (m, 2H), 1.80 (t, J = 12.6 Hz each, 1H), 1.16 (d, J = 6.4 Hz, 3H). ^{13}C NMR (200 MHz, D_2O) δ 175.22, 173.86, 173.48, 100.78, 100.50, 99.24, 98.02, 74.67, 74.50, 72.77, 72.42, 72.11, 71.72, 71.51, 70.94, 68.79, 68.59, 67.82, 67.68, 67.50, 67.36, 66.93, 66.54, 66.23, 65.59, 62.02, 60.47, 55.01, 50.90, 39.20, 37.44, 26.12, 21.67, 14.85. HRMS (ESI) *m/z* calculated for $C_{34}H_{58}N_3O_{30}S_2$ (M-H) 1052.2552, found 1052.1559.

References:

1. H. Yu, R. Karpel and X. Chen, *Bioorg. Med. Chem.*, 2004, **12**, 6427.
2. W. Yi, X. Liu, Y. Li, J. Li, C. Xia, G. Zhou, W. Zhang, W. Zhao, X. Chen and P. G. Wang, *Proc. Natl. Acad. Sci. U. S. A.*, 2009, **106**, 4207.
3. K. Lau, V. Thon, H. Yu, L. Ding, Y. Chen, M. M. Muthana, D. Wong, R. Huang and X. Chen, *Chem. Commun.*, 2010, **46**, 6066.
4. G. Sugiarto, K. Lau, Y. Li, Z. Khedri, H. Yu, D. T. Le and X. Chen, *Mol. Biosyst.*, 2011, **7**, 3021.
5. G. Sugiarto, K. Lau, J. Qu, Y. Li, S. Lim, S. Mu, J. B. Ames, A. J. Fisher and X. Chen, *ACS Chem. Biol.*, 2012, **7**, 1232.
6. P. K. Mandal and W. B. Turnbull, *Carbohydr. Res.*, 2011, **346**, 2113.
7. M. P. DeNinno, J. B. Etienne and K. C. Duplantier, *Tetrahedron Lett.*, 1995, **36**, 669.
8. H. Cao, S. Huang, J. Cheng, Y. Li, S. Muthana, B. Son and X. Chen, *Carbohydr. Res.*, 2008, **343**, 2863.
9. Z. B. Szabó, A. Borbás, I. Bajza and A. Lipták, *Tetrahedron Asymm.*, 2005, **16**, 83.
10. S. Komba, H. Ishida, M. Kiso and A. Hasegawa, *Bioorg. Med. Chem.*, 1996, **4**, 1833.
11. T. Ghosh, A. Santra and A. K. Misra, *RSC Adv.*, 2014, **4**, 54.
12. B. Yang, K. Yoshida, Z. Yin, H. Dai, H. Kavunja, M. H. El-Dakdouki, S. Sungsuwan, S. B. Dulaney and X. Huang, *Angew. Chem. Int. Ed. Engl.*, 2012, **51**, 10185.
13. S. B. Dulaney and X. Huang, *Adv. Carbohydr. Chem. Biochem.*, 2012, **67**, 95.
14. W. M. Pearlman, *Tetrahedron Lett.*, 1967, 1663.
15. H. Yu, K. Lau, Y. Li, G. Sugiarto and X. Chen, *Curr. Protoc. Chem. Biol.*, 2012, **4**, 233.
16. Y. Li, H. Yu, H. Cao, S. Muthana and X. Chen, *Appl. Microbiol. Biotechnol.*, 2012, **93**, 2411.

^{1}H and ^{13}C NMR spectra of 6-*O*-sulfo-LacNAcβProN$_3$ (**4**)

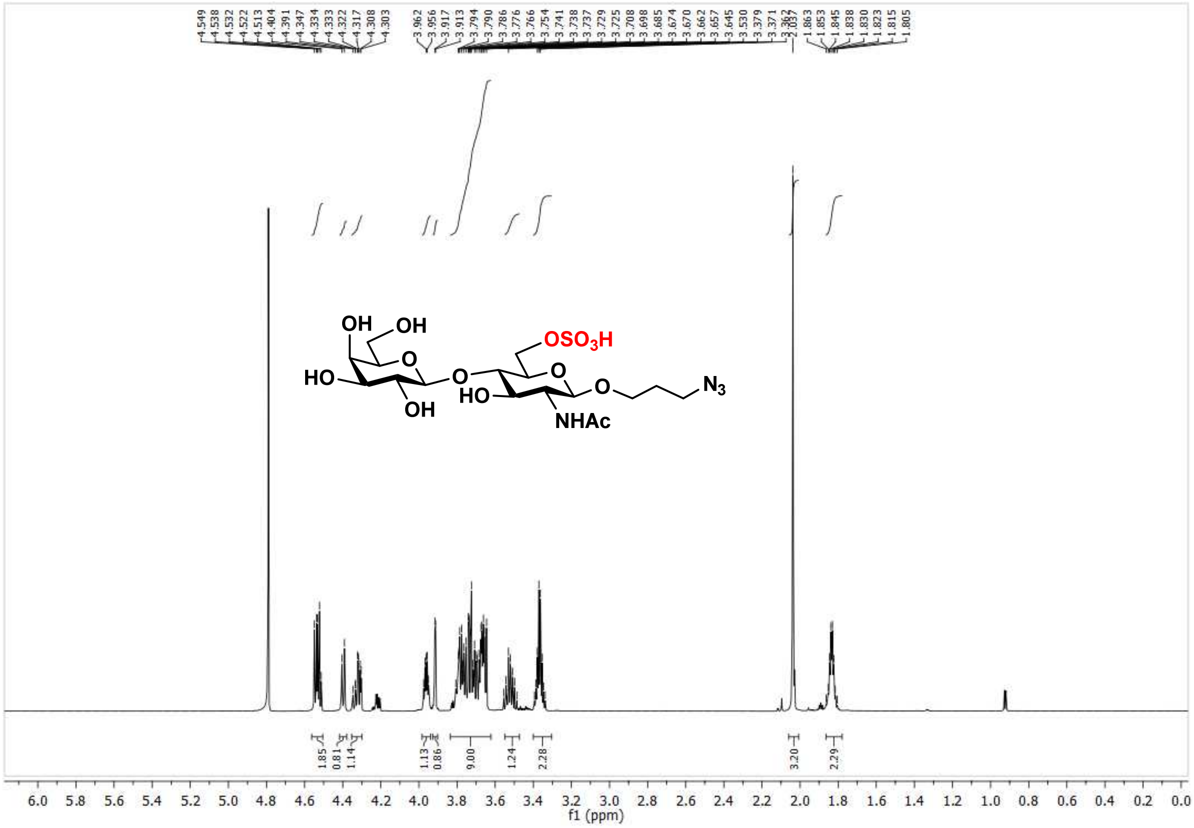

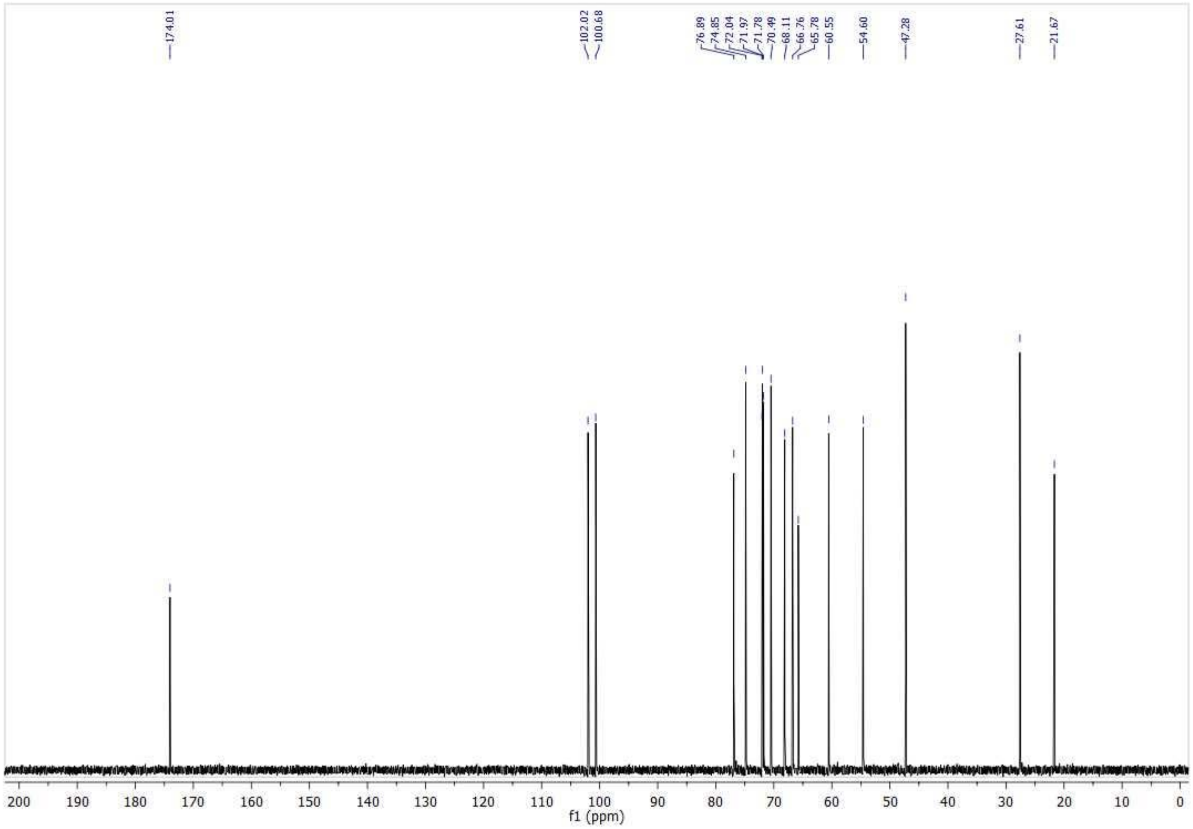

^{1}H and ^{13}C NMR spectra of 6'-*O*-sulfo-LacNAcβProN$_3$ (**5**)

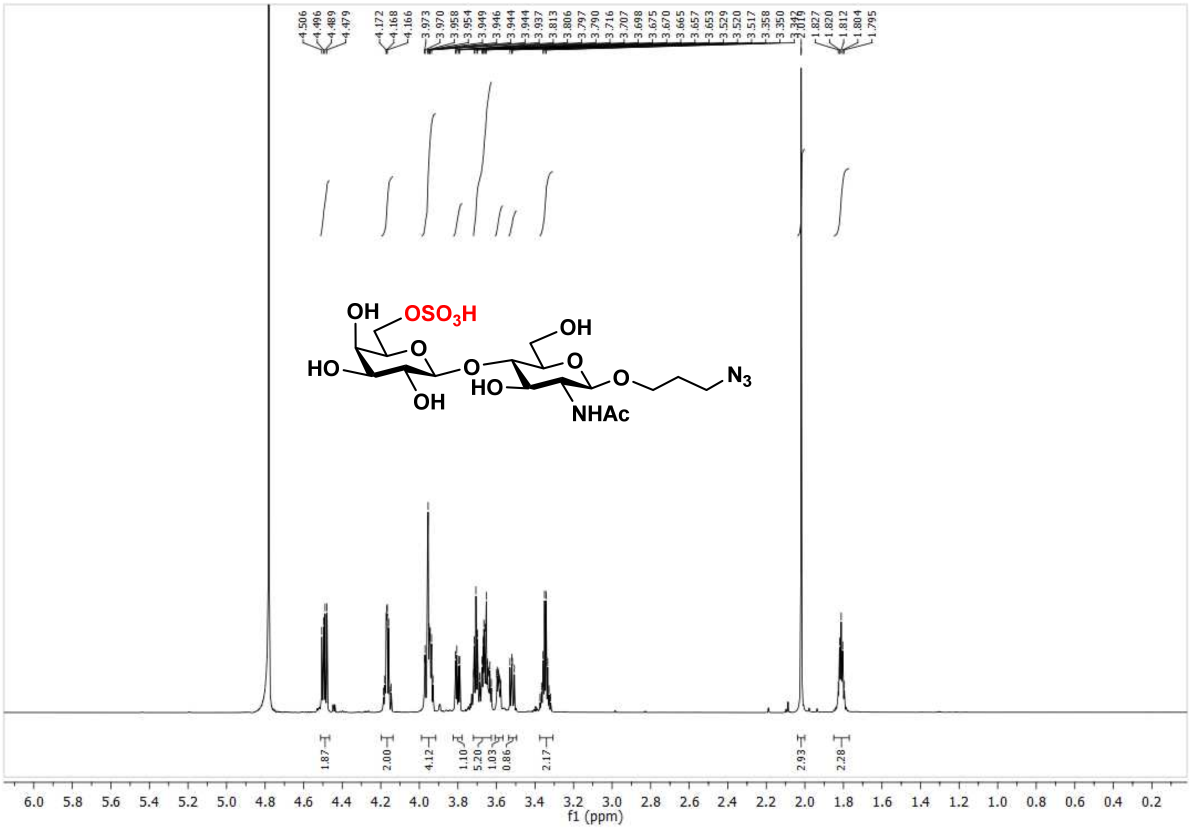

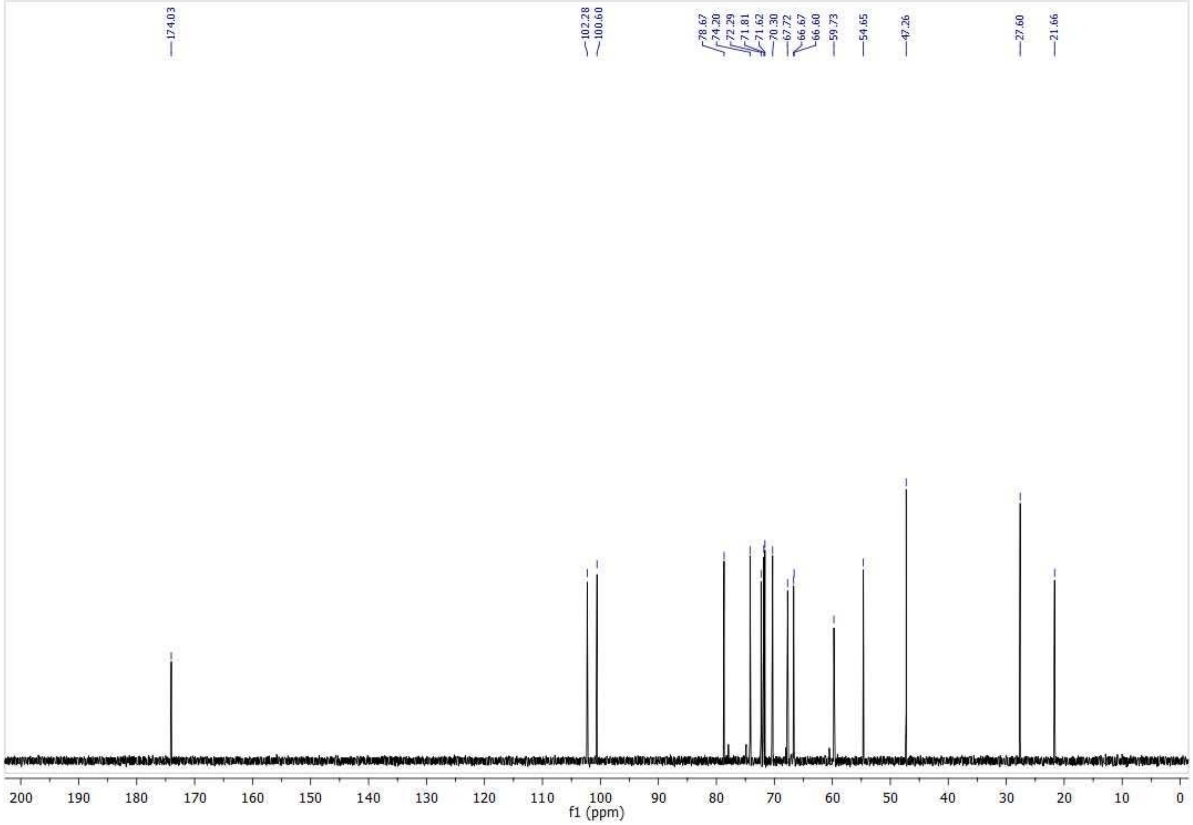

^{1}H and ^{13}C NMR spectra of 6,6'-di-*O*-sulfo-LacNAcβProN$_3$ (**6**)

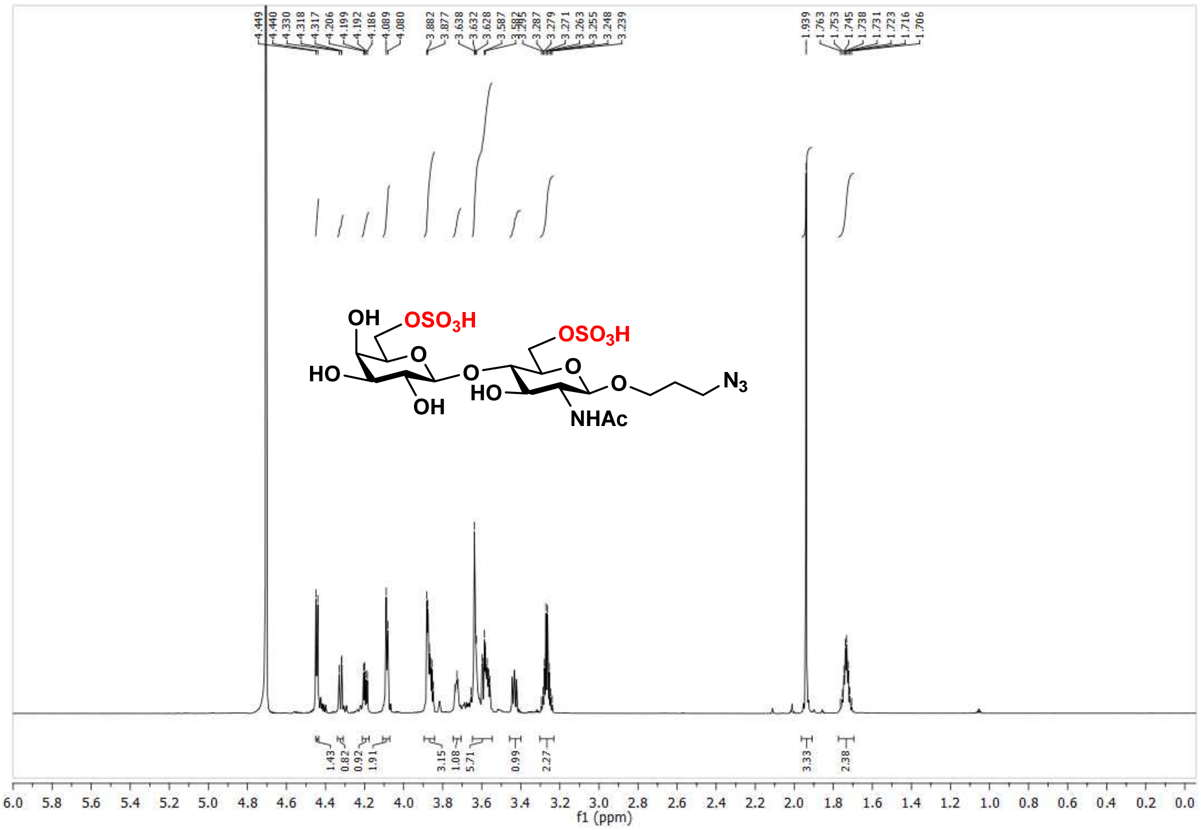

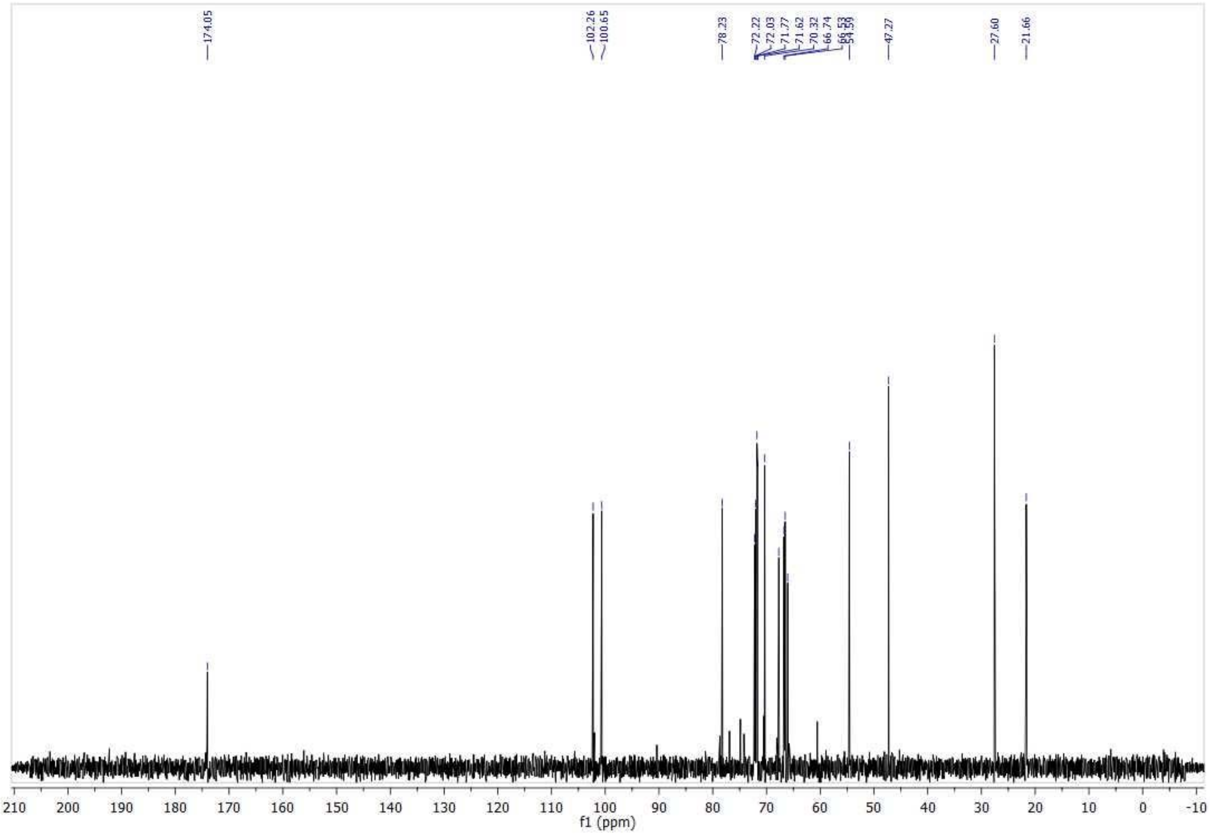

^{1}H and ^{13}CNMR spectra of 6-*O*-sulfo-Le[x]βProN$_3$ (**8**)

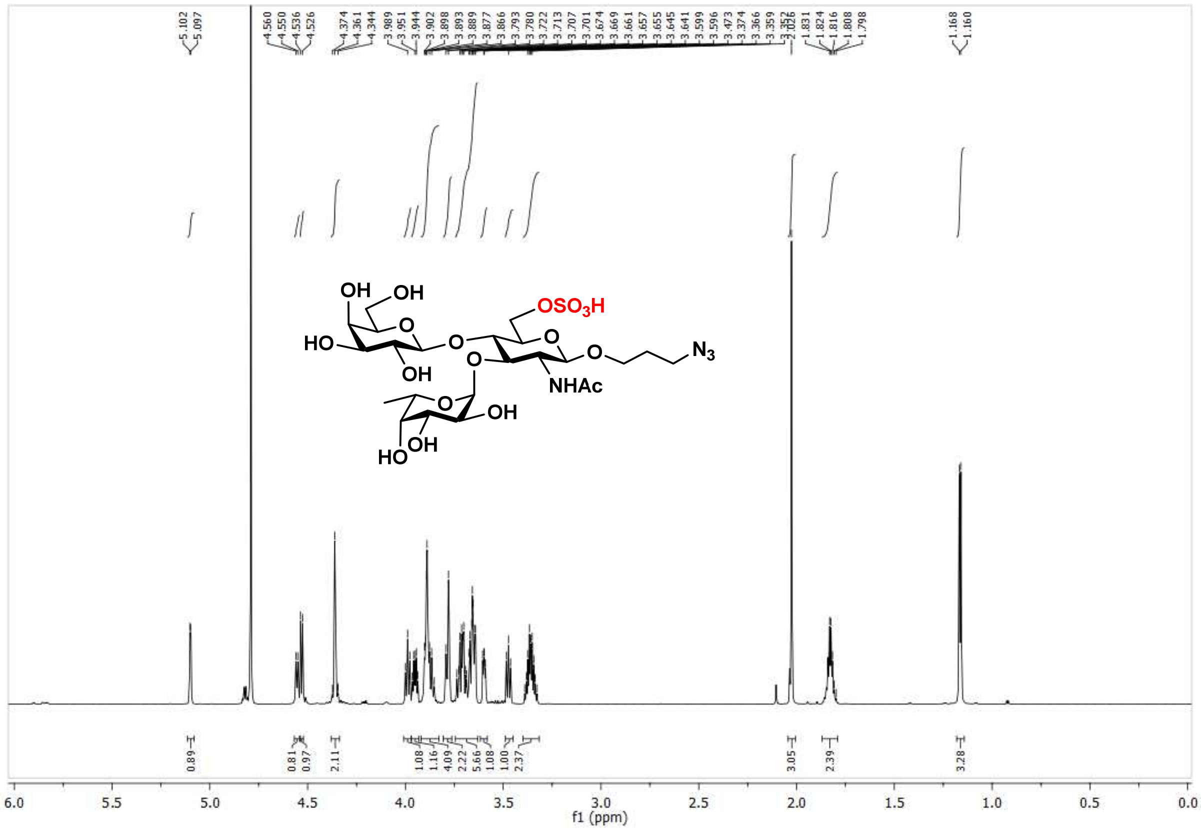

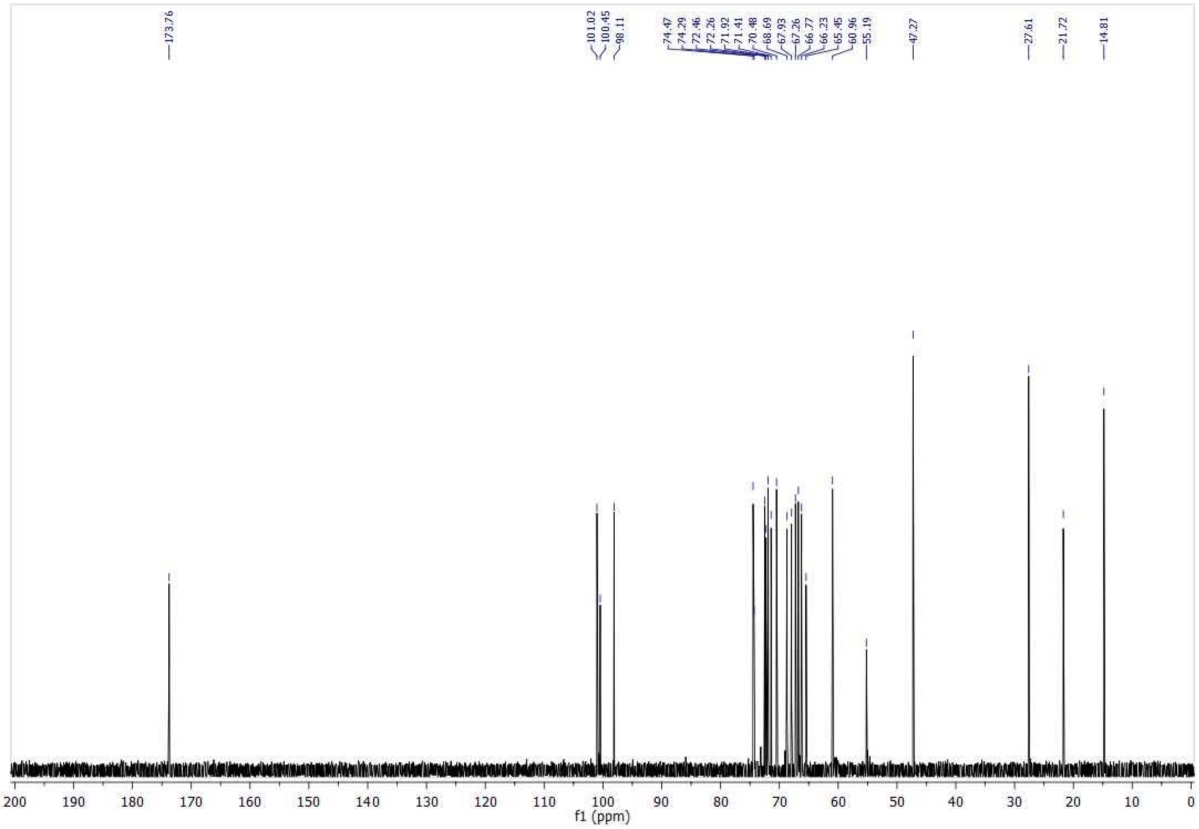

^{1}H and^{13}C NMR spectra of 6'-*O*-sulfo-Le^{x}βProNH$_2$(**9**)

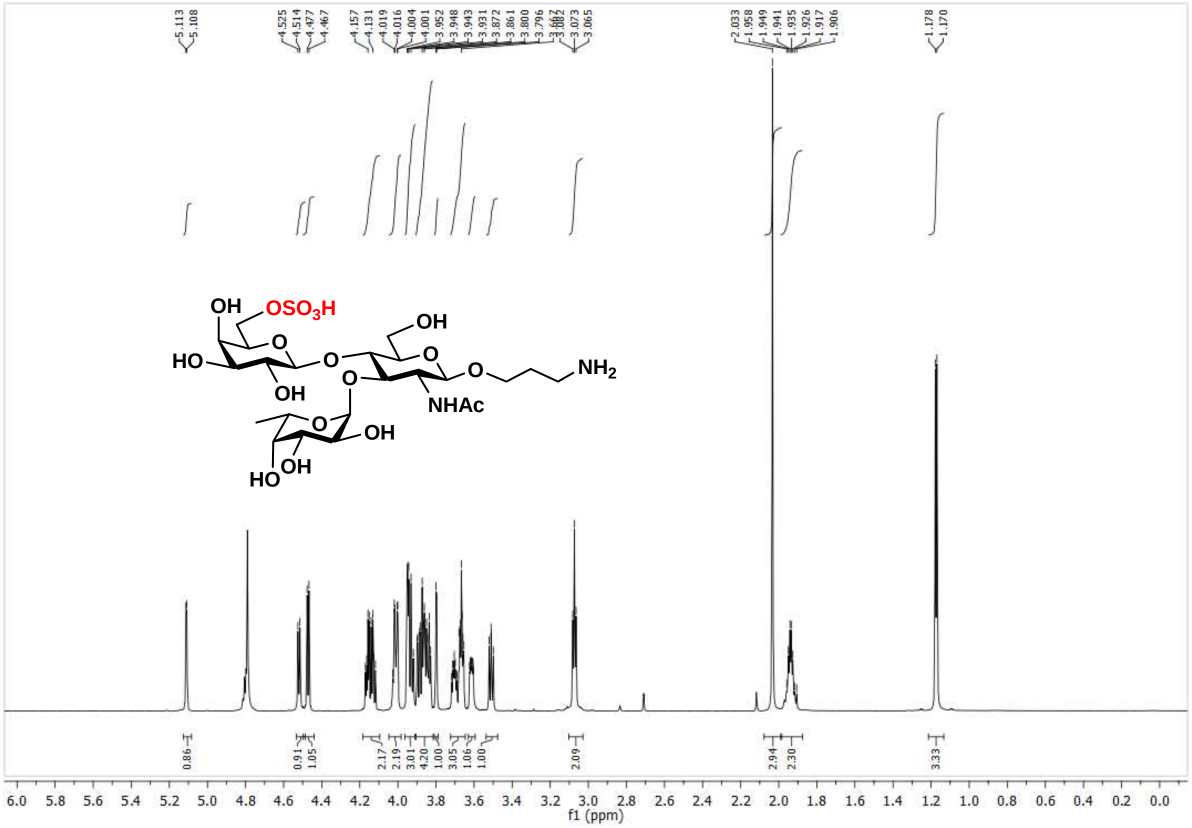

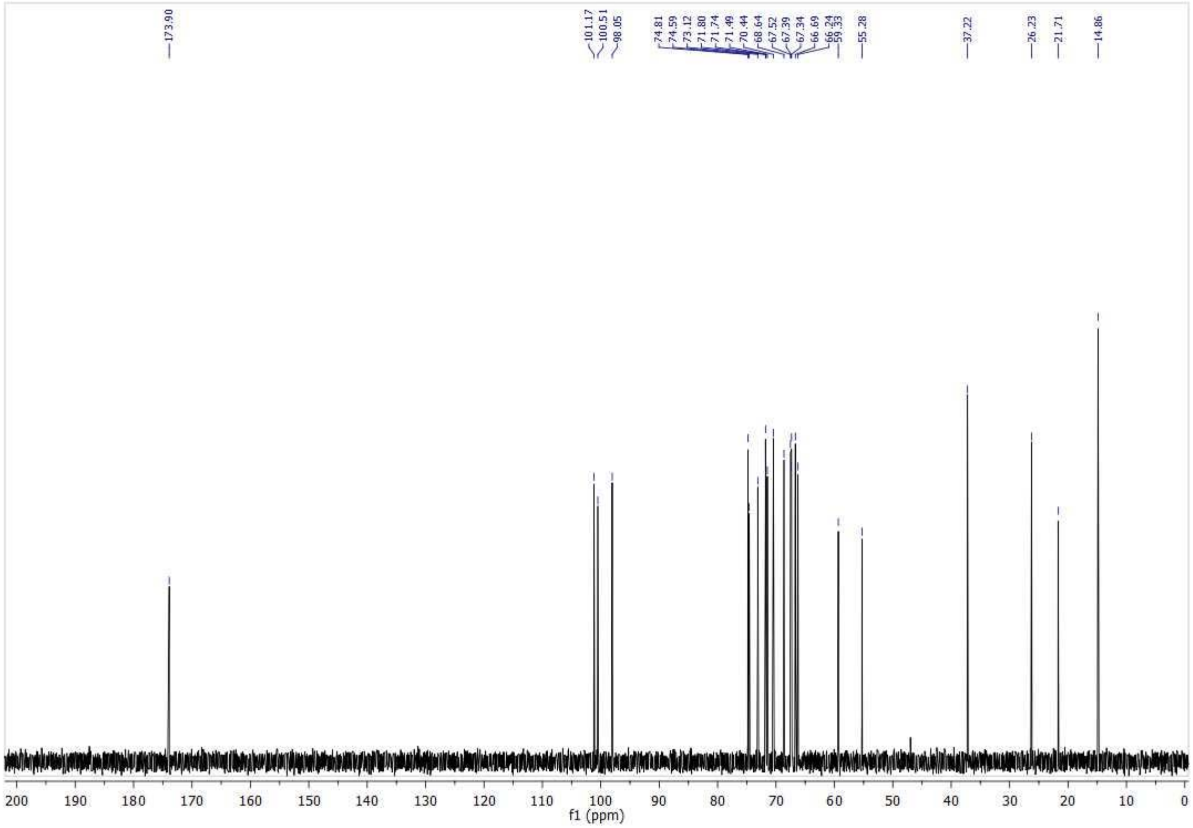

^{1}H and ^{13}C NMR spectra of 6',6-di-*O*-sulfo-Le^{x}βProNH$_2$(**10**)

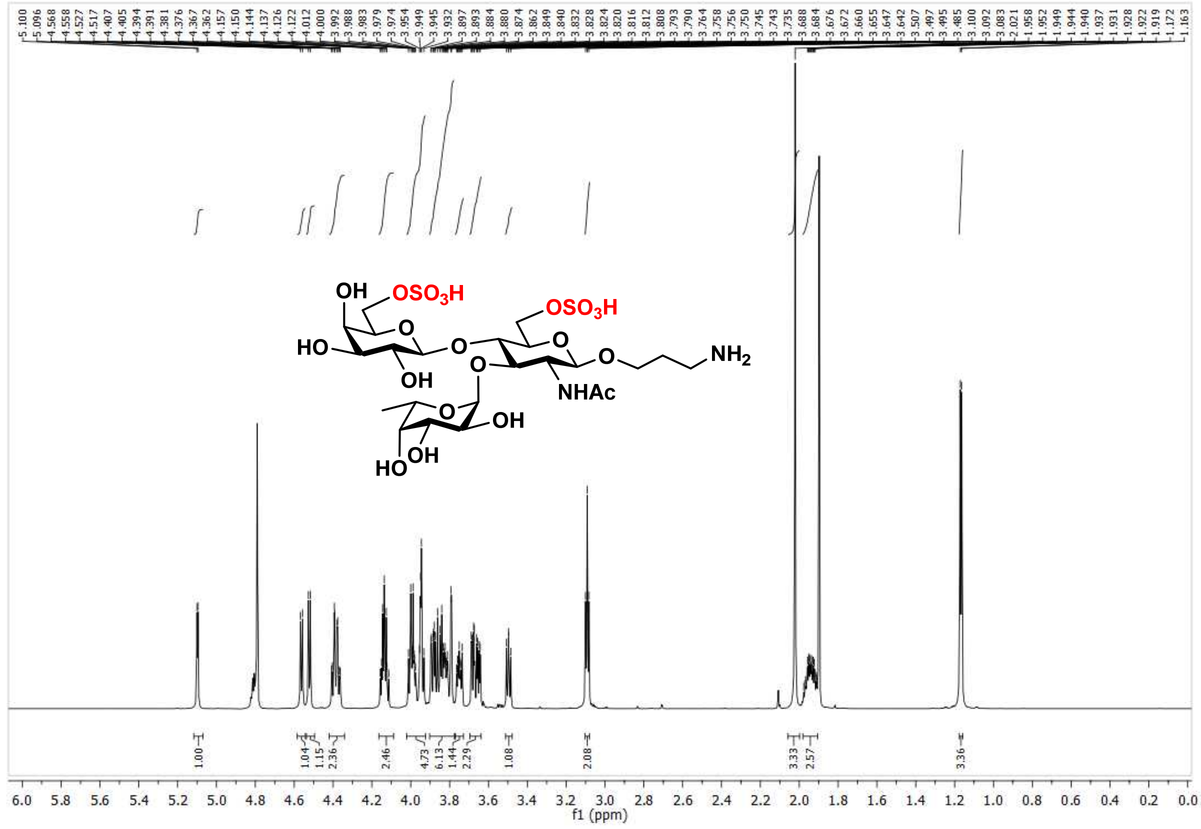

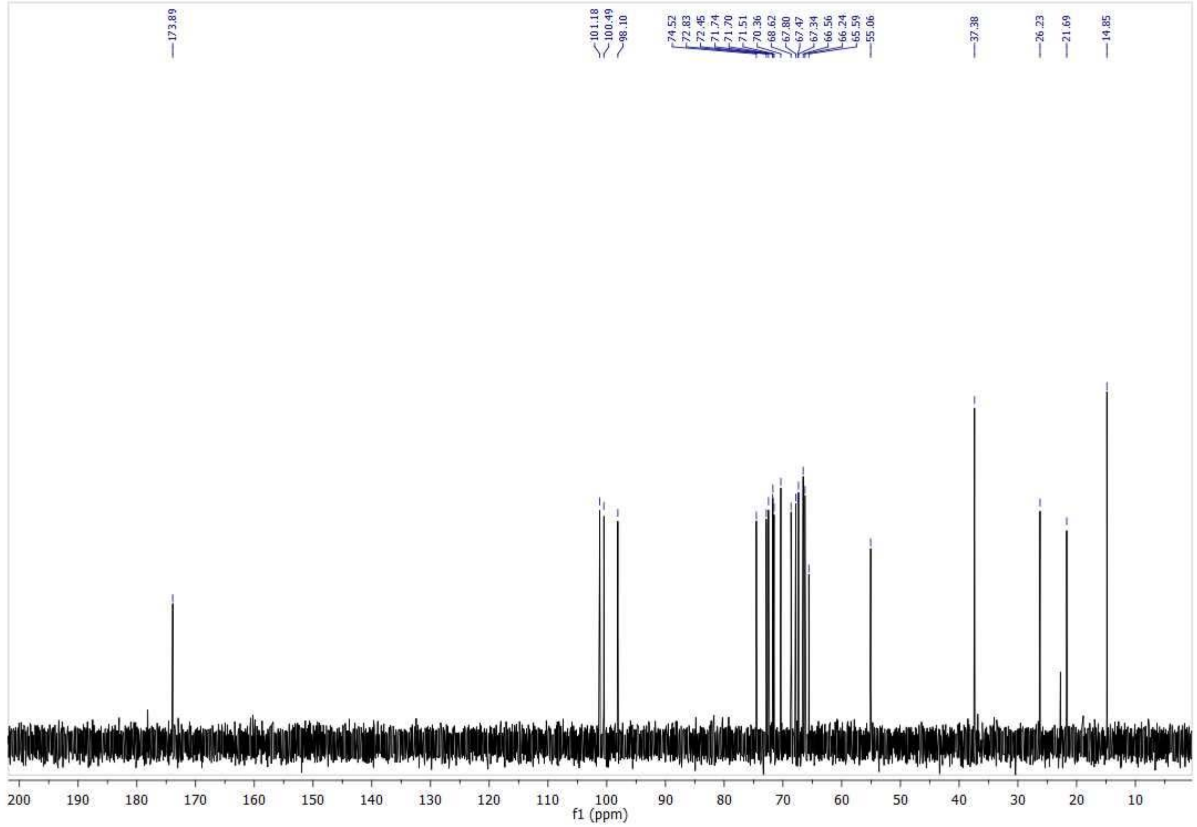

^{1}H and ^{13}C NMR spectra of 6-*O*-sulfo-Neu5Acα2–3Le^{x}βProN$_3$ (**1a**)

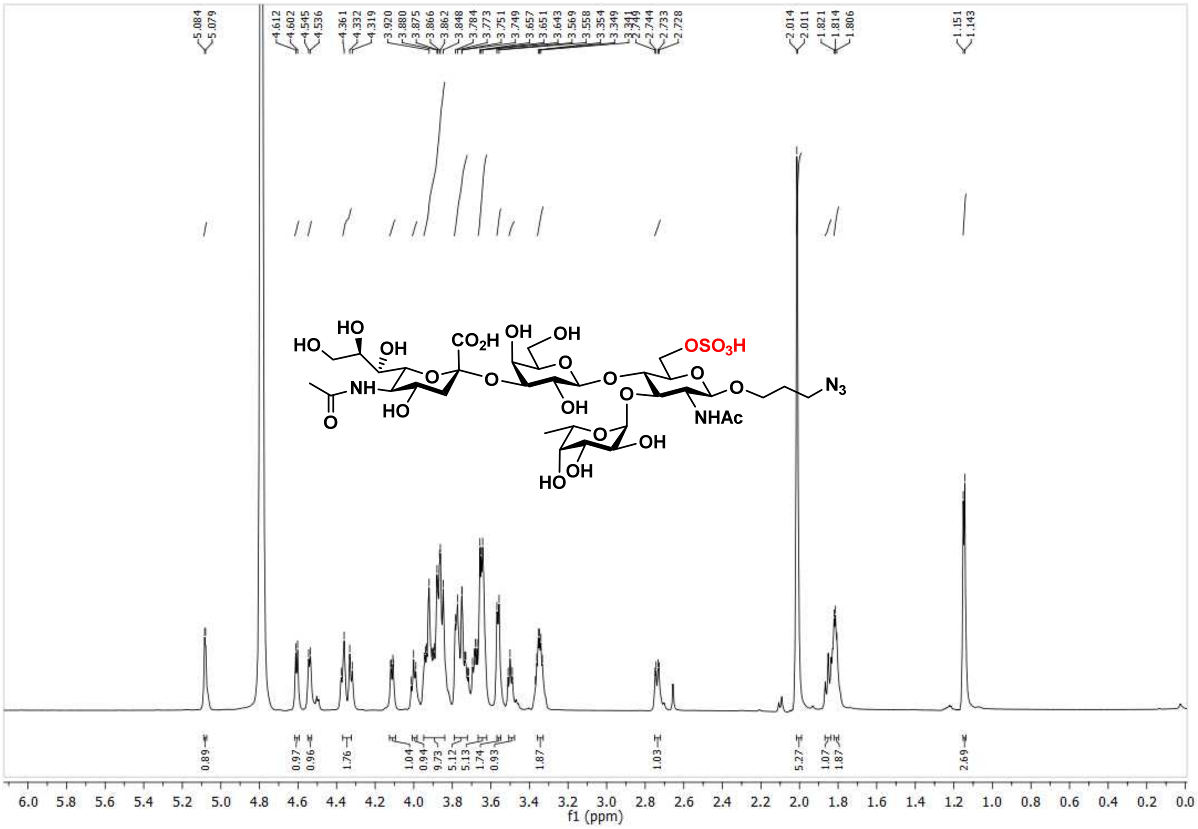

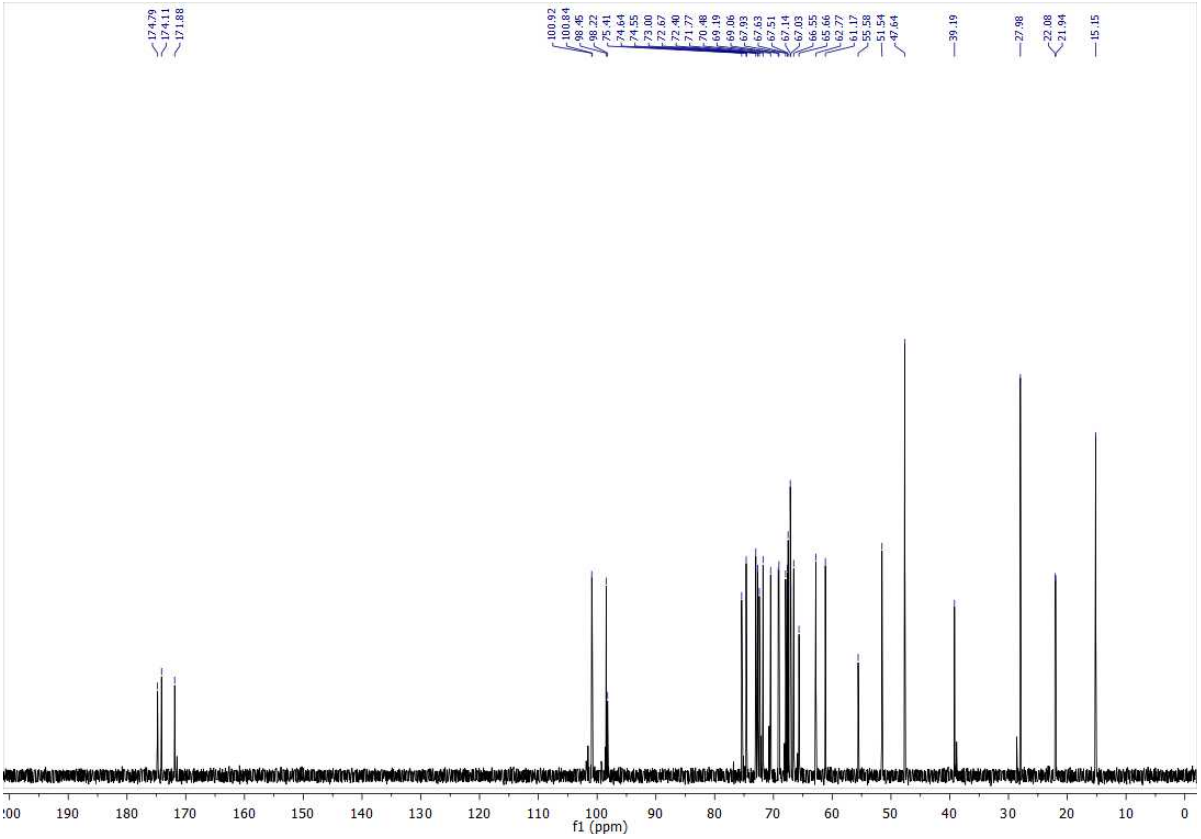

^{1}H and ^{13}C NMR spectra of 6-*O*-sulfo-Neu5Gcα2–3Le^{x}βProN$_3$ (**1b**)

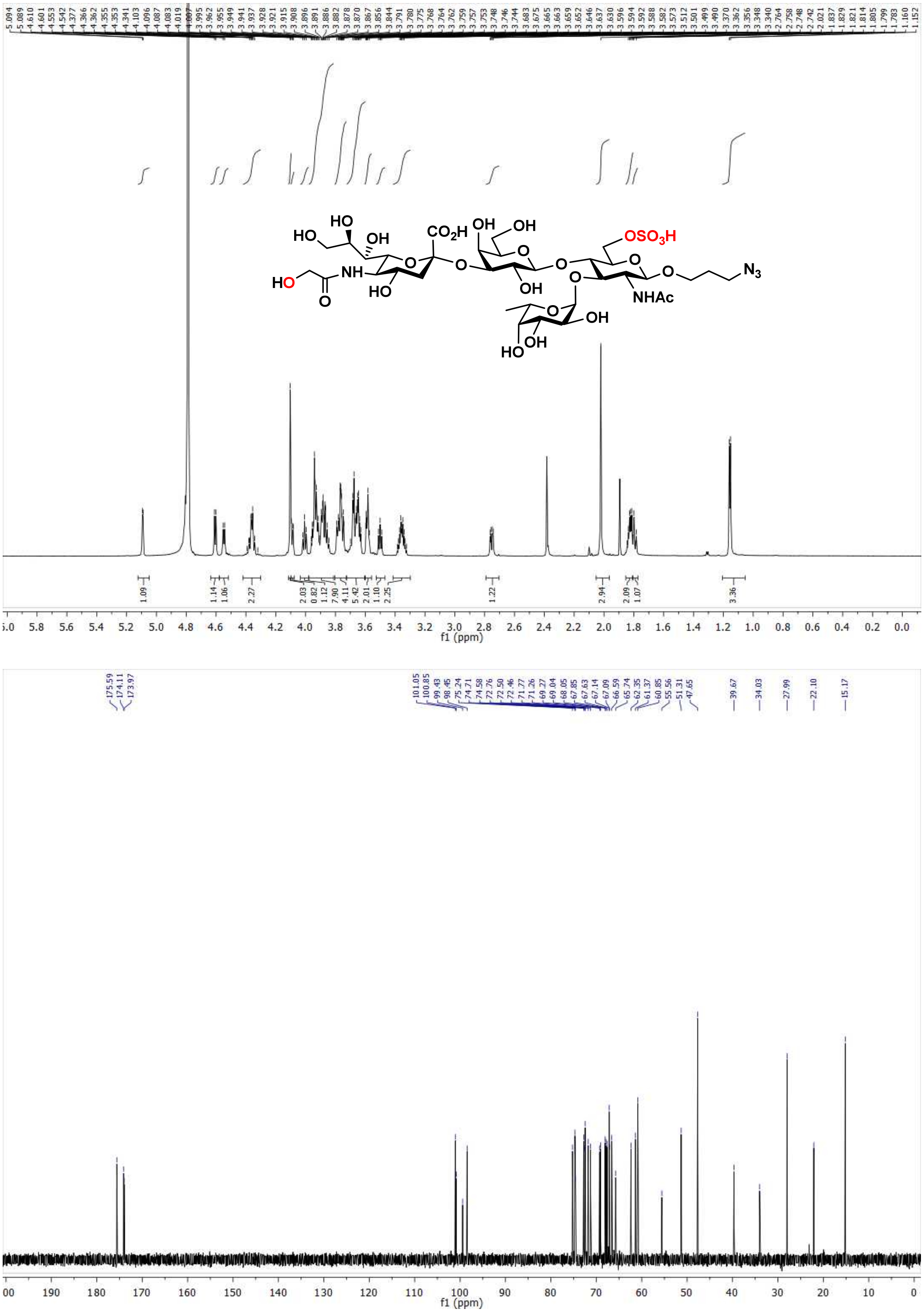

^{1}H and ^{13}C NMR spectra of 6'-*O*-sulfo-Neu5Acα2–3Le[x]βProNH$_2$ (**2a**)

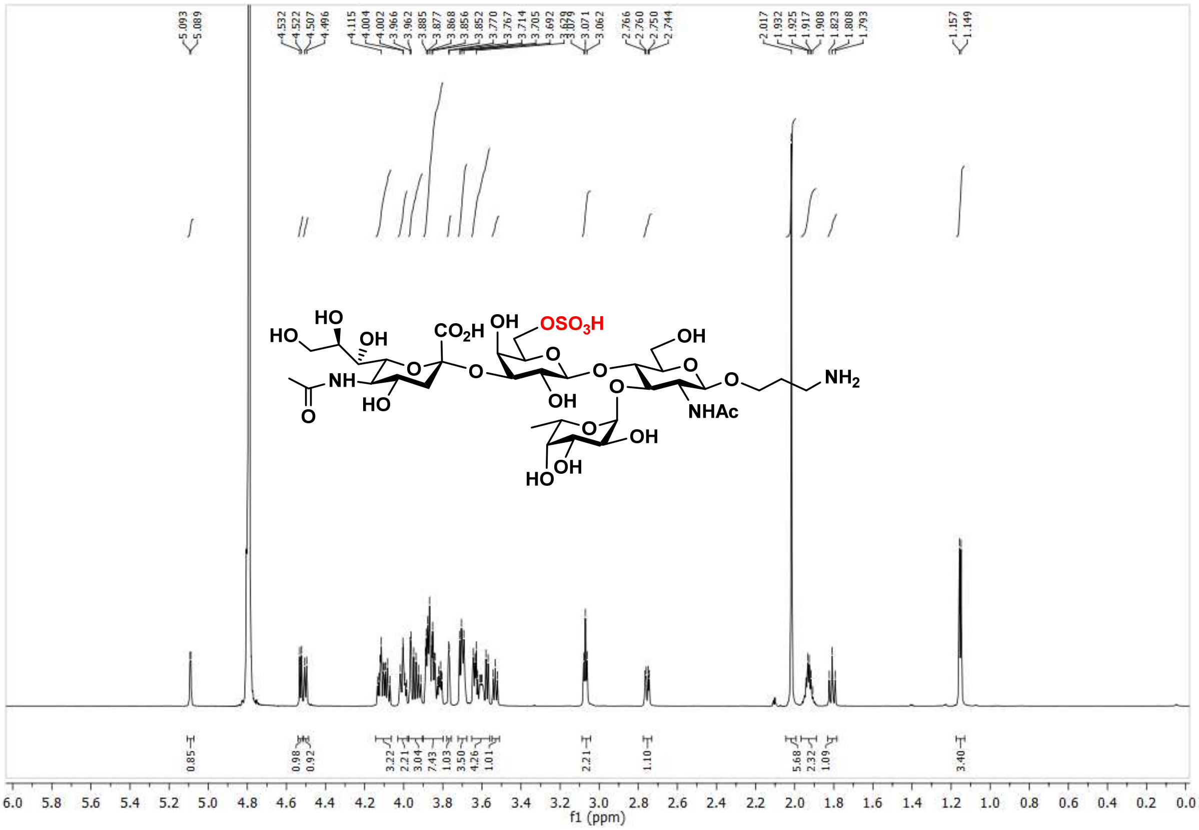

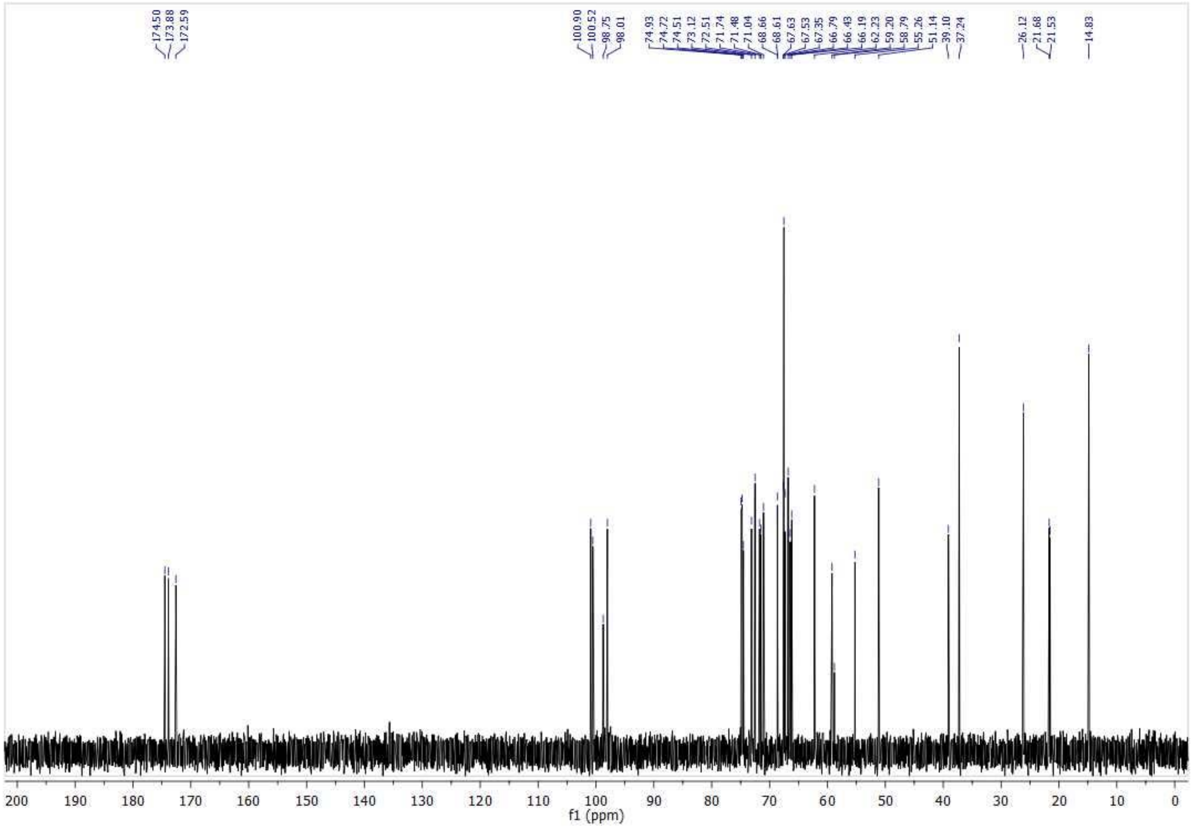

^{1}H and ^{13}C NMR spectra of 6'-*O*-sulfo-Neu5Gcα2–3Le^{x}βProNH$_2$ (**2b**)

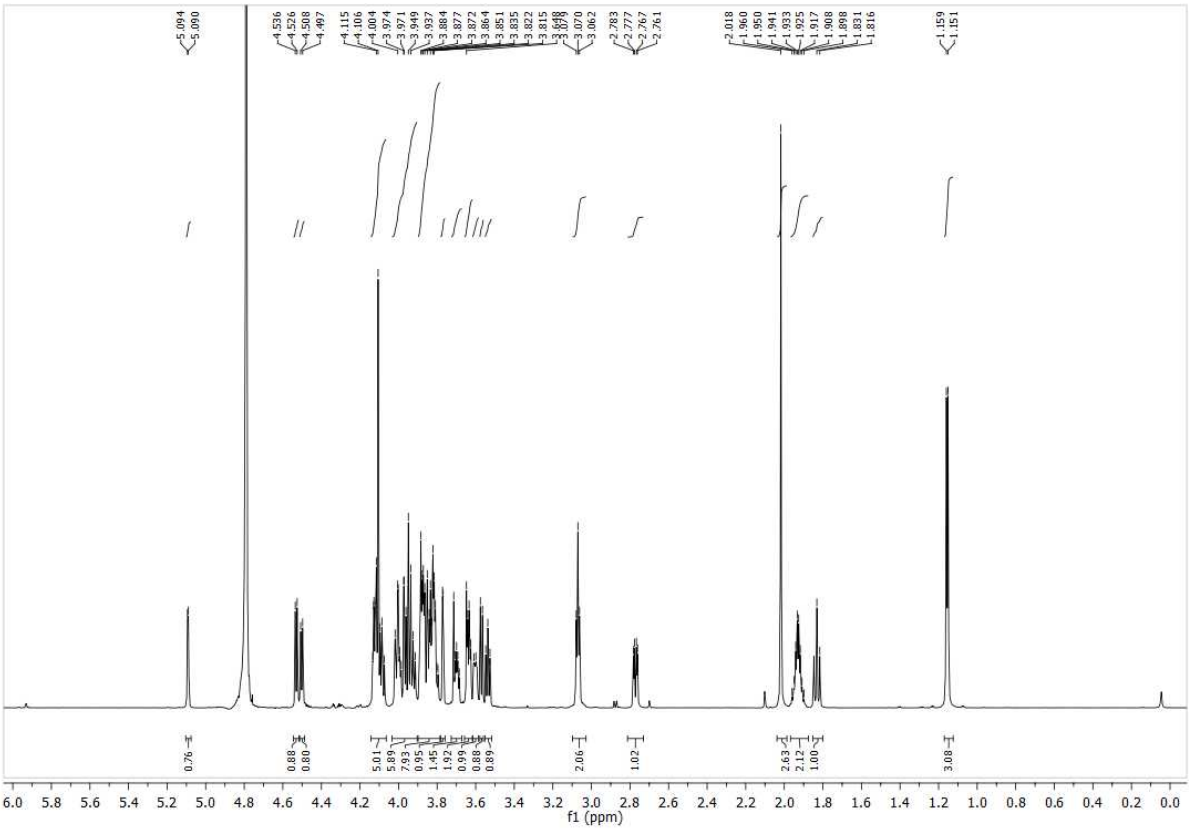

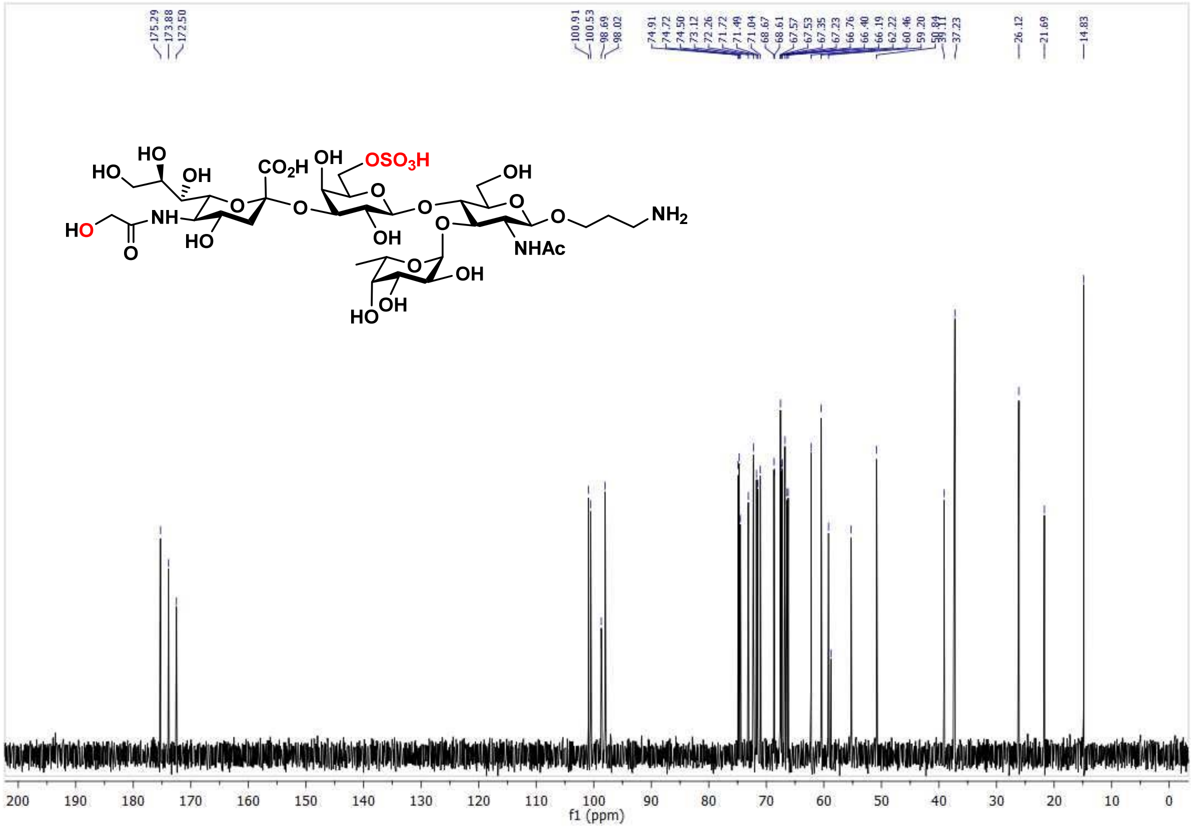

^{1}H and ^{13}C NMR spectra of 6,6'-di-*O*-sulfo-Neu5Acα2–3Le^{x}βProNH$_2$ (**3a**)

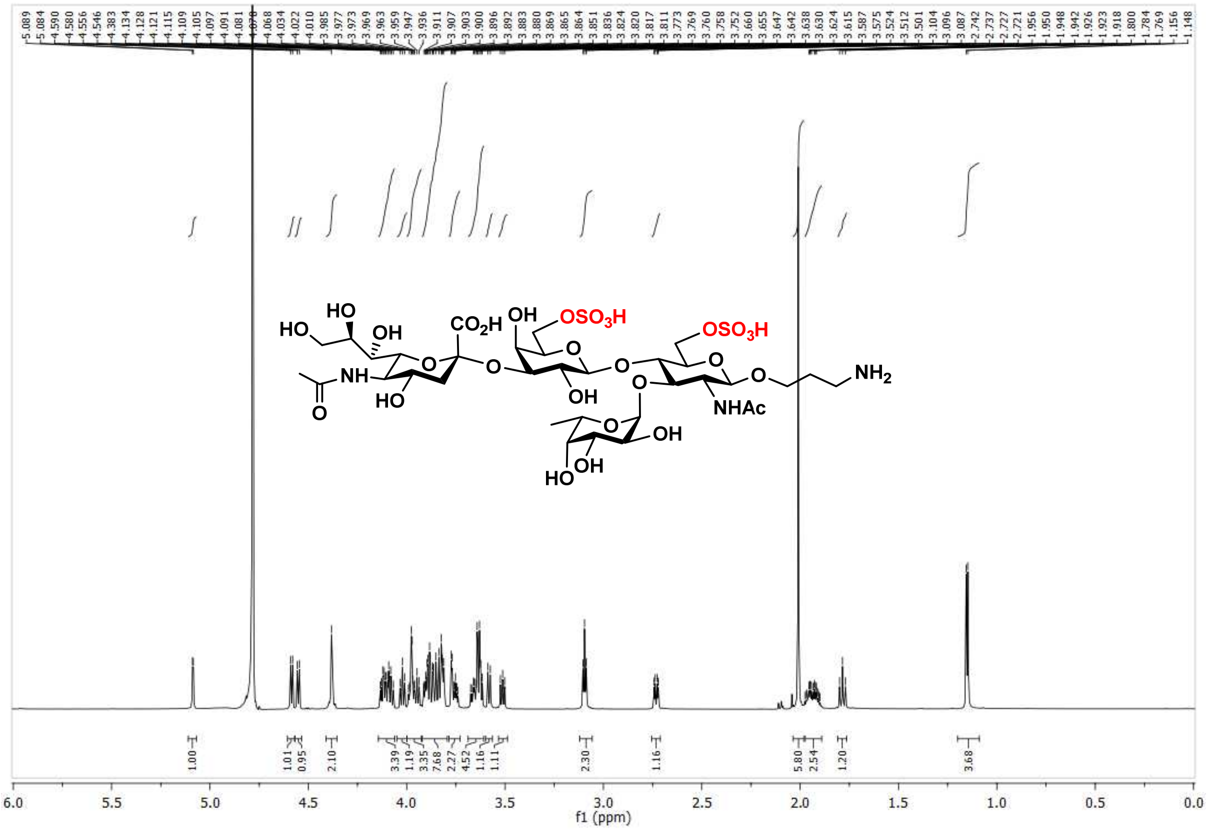

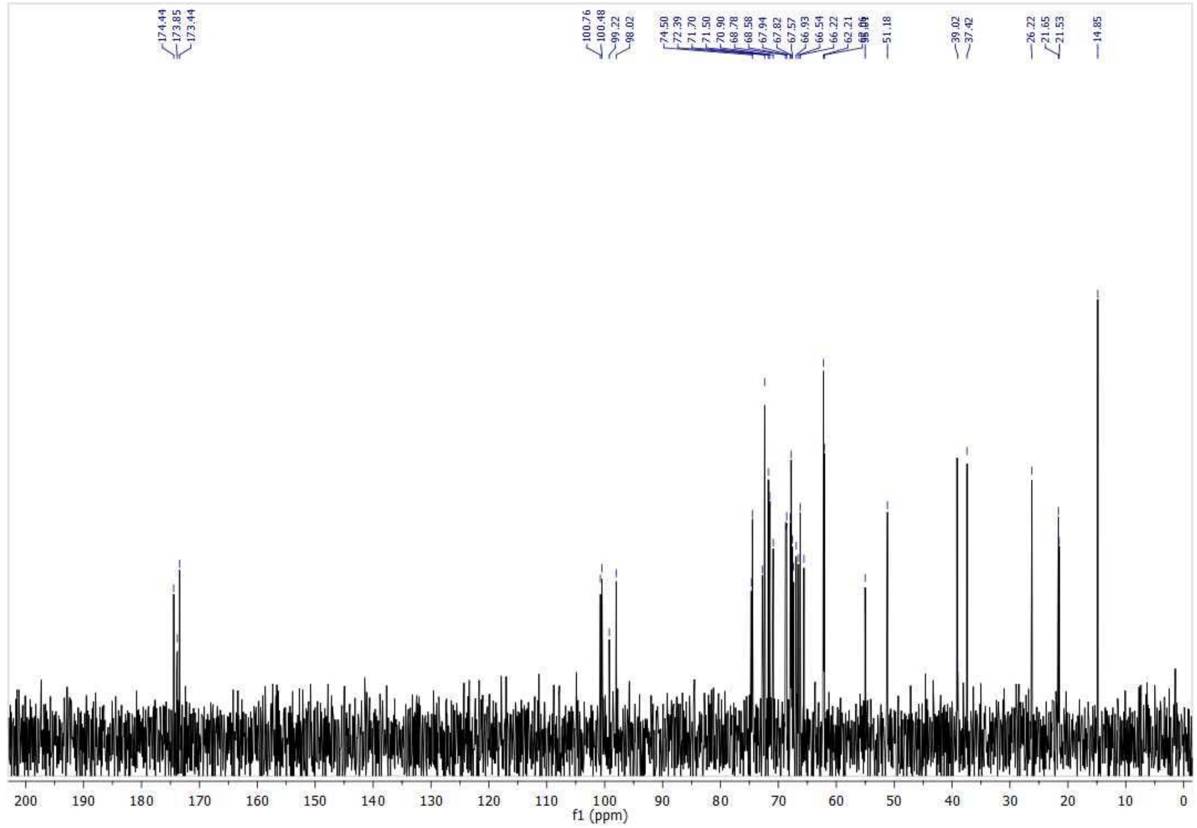

^{1}H and ^{13}C NMR spectra of 6,6'-di-*O*-sulfo-Neu5Gcα2–3Le[x]βProNH$_2$ (**3b)**

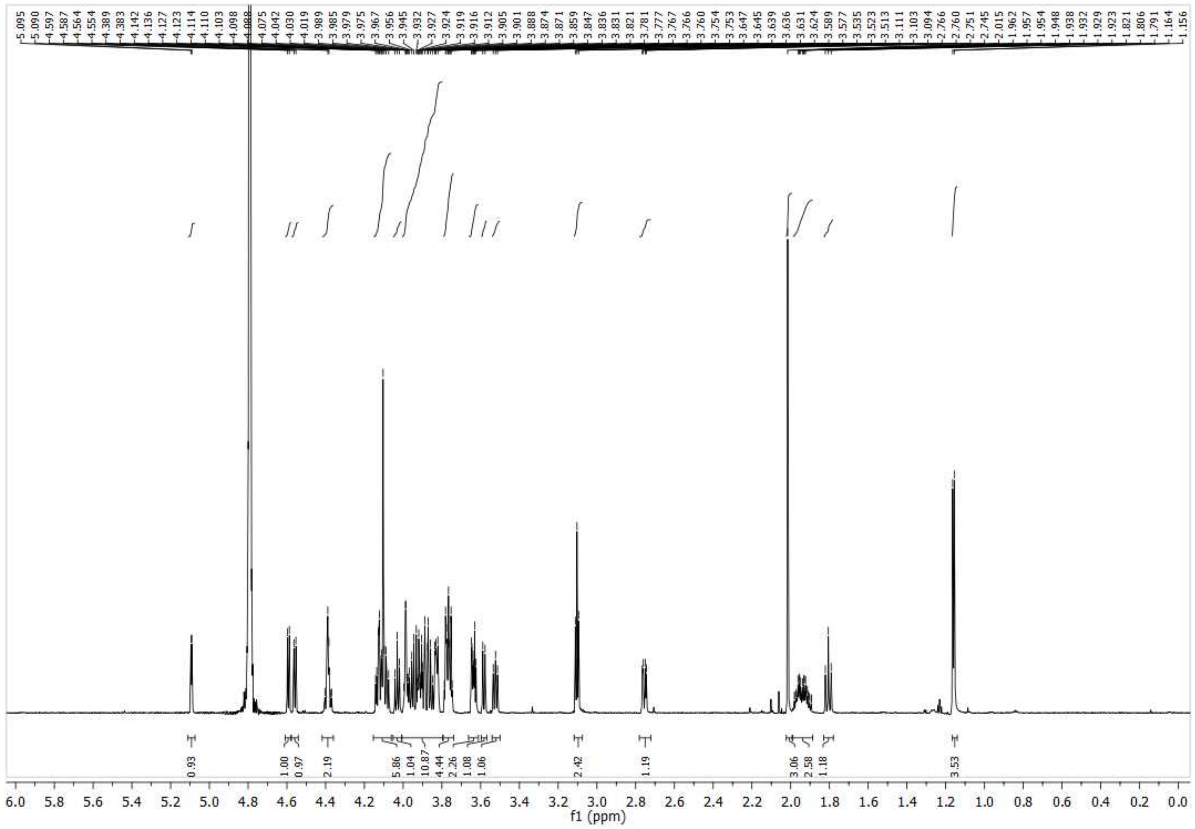

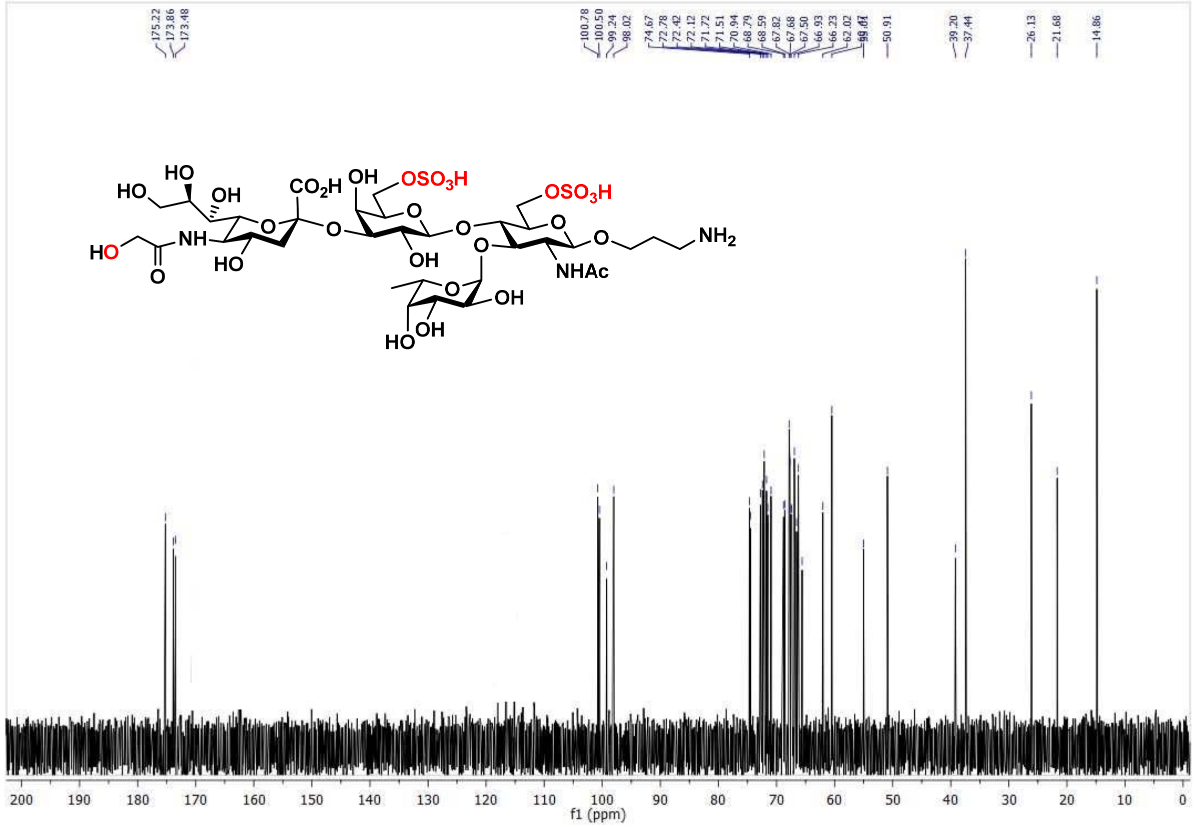

Supplement: Supplementary file 1 [file SC-007-C5SC04104J-s001.pdf]
